# Deep learning–based automatic segmentation of meningioma from T1-weighted contrast-enhanced MRI for preoperative meningioma differentiation using radiomic features

**DOI:** 10.1186/s12880-024-01218-3

**Published:** 2024-03-05

**Authors:** Liping Yang, Tianzuo Wang, Jinling Zhang, Shi Kang, Shichuan Xu, Kezheng Wang

**Affiliations:** 1 Department of PET-CT, Harbin Medical University Cancer Hospital, Harbin, 150001 China; 2 Medical Imaging Department, Changzheng Hospital of Harbin City, Harbin, China; 3 Medical Imaging Department, The Second Affiliated Hospital of Harbin Medical University, Harbin, China; 4 Medical Imaging Department, The Second Hospital of Heilongjiang Province, Harbin, China; 5 Department of Medical Instruments, Second Hospital of Harbin, Harbin, 150001 China

**Keywords:** Meningiomas, Magnetic resonance imaging, Radiomics, Deep learning, Segmentation

## Abstract

**Background:**

This study aimed to establish a dedicated deep-learning model (DLM) on routine magnetic resonance imaging (MRI) data to investigate DLM performance in automated detection and segmentation of meningiomas in comparison to manual segmentations. Another purpose of our work was to develop a radiomics model based on the radiomics features extracted from automatic segmentation to differentiate low- and high-grade meningiomas before surgery.

**Materials:**

A total of 326 patients with pathologically confirmed meningiomas were enrolled. Samples were randomly split with a 6:2:2 ratio to the training set, validation set, and test set. Volumetric regions of interest (VOIs) were manually drawn on each slice using the ITK-SNAP software. An automatic segmentation model based on SegResNet was developed for the meningioma segmentation. Segmentation performance was evaluated by dice coefficient and 95% Hausdorff distance. Intra class correlation (ICC) analysis was applied to assess the agreement between radiomic features from manual and automatic segmentations. Radiomics features derived from automatic segmentation were extracted by pyradiomics. After feature selection, a model for meningiomas grading was built.

**Results:**

The DLM detected meningiomas in all cases. For automatic segmentation, the mean dice coefficient and 95% Hausdorff distance were 0.881 (95% CI: 0.851–0.981) and 2.016 (95% CI:1.439–3.158) in the test set, respectively. Features extracted on manual and automatic segmentation are comparable: the average ICC value was 0.804 (range, 0.636–0.933). Features extracted on manual and automatic segmentation are comparable: the average ICC value was 0.804 (range, 0.636–0.933). For meningioma classification, the radiomics model based on automatic segmentation performed well in grading meningiomas, yielding a sensitivity, specificity, accuracy, and area under the curve (AUC) of 0.778 (95% CI: 0.701–0.856), 0.860 (95% CI: 0.722–0.908), 0.848 (95% CI: 0.715–0.903) and 0.842 (95% CI: 0.807–0.895) in the test set, respectively.

**Conclusions:**

The DLM yielded favorable automated detection and segmentation of meningioma and can help deploy radiomics for preoperative meningioma differentiation in clinical practice.

**Supplementary Information:**

The online version contains supplementary material available at 10.1186/s12880-024-01218-3.

## Introduction

Meningioma, originated from the cell of the middle layer of meninges and the arachnoid, is the most common histopathological type of adult central nervous system (CNS) tumor [1]. The 2021 World Health Organization (WHO) classification system of CNS tumors (5^th^ edition) has not substantially changed the management of meningioma patients, and the WHO grades still guide its treatment decisions, which affects the patients’ survival prognosis to a certain extent [2]. Compared with low-grade meningiomas (WHO grade I), high-grade meningiomas (WHO II/III grade) exhibit more aggressive biological behavior, a more pronounced tendency to recur, and a poorer clinical prognosis [3–5]. According to statistical reports, the 10-year survival rate of malignant (high-grade) meningiomas is only 60%, while for non-malignant (low-grade) meningiomas, it is approximately 83.4% [6]. In clinical practice, non-invasive and accurate identification of the grade of meningioma is of great significance.

Magnetic resonance imaging (MRI) technology has been widely used in the preoperative diagnosis of meningiomas due to its excellent soft tissue resolution [7]. Especially, the T1-weighted contrast-enhanced (T1W-CE) sequence could provide a large amount of blood supply information to display tumor tissue as clearly as possible [8]. However, it has to be admitted that the imaging manifestations of different grades of meningiomas are largely overlapped, leading to inaccurate grading and improper treatment measures [9]. So far, there is still a lack of a widely accepted method to accurately predict histopathological grading in clinical practice.

Recently, radiomics and deep learning (DL), two main categories of machine learning (ML), have rapidly developed into a research hotspot in medical image analysis, enabling the extraction of high-throughput quantitative imaging features from medical image [10–12]. It captures relationships between image voxels that may not be perceived by the naked eye of physicians-even experienced radiologists, which can contribute to the diagnostic and predictive accuracy of the disease. A previous study had proven that radiomics is a valid tool for grading meningioma, and it has outperformed the subjective diagnosis of experienced doctors [13]. Tumor segmentation is the first and major step in radiomics analysis, but the manual segmentation of tumor lesions is a laborious ordeal and time-consuming, and high inter- and intra-reader variability is not negligible [14]. It is worth considering whether the DL-based automatic segmentation in the radiomic analysis of meningioma differentiation can replace time-consuming manual segmentation. The neural network with a U-shape architecture is a promising tool for automatic segmentation even under the condition of limited sample size, as has been reported in glioma patients [15–17]. To the best of our knowledge, few studies attempted to develop an ML pipeline for meningiomas grading using automatic segmentation. Therefore, we decided to establish a dedicated deep-learning model (DLM) on routine MRI data to investigate DLM performance in automated detection and segmentation of meningiomas in comparison to manual segmentations. Another purpose of our work was to develop a radiomics model based on the radiomics features extracted from automatic segmentation to differentiate low- and high-grade meningiomas before surgery.

## Material and methods

### Study population

**Fig. 1:**
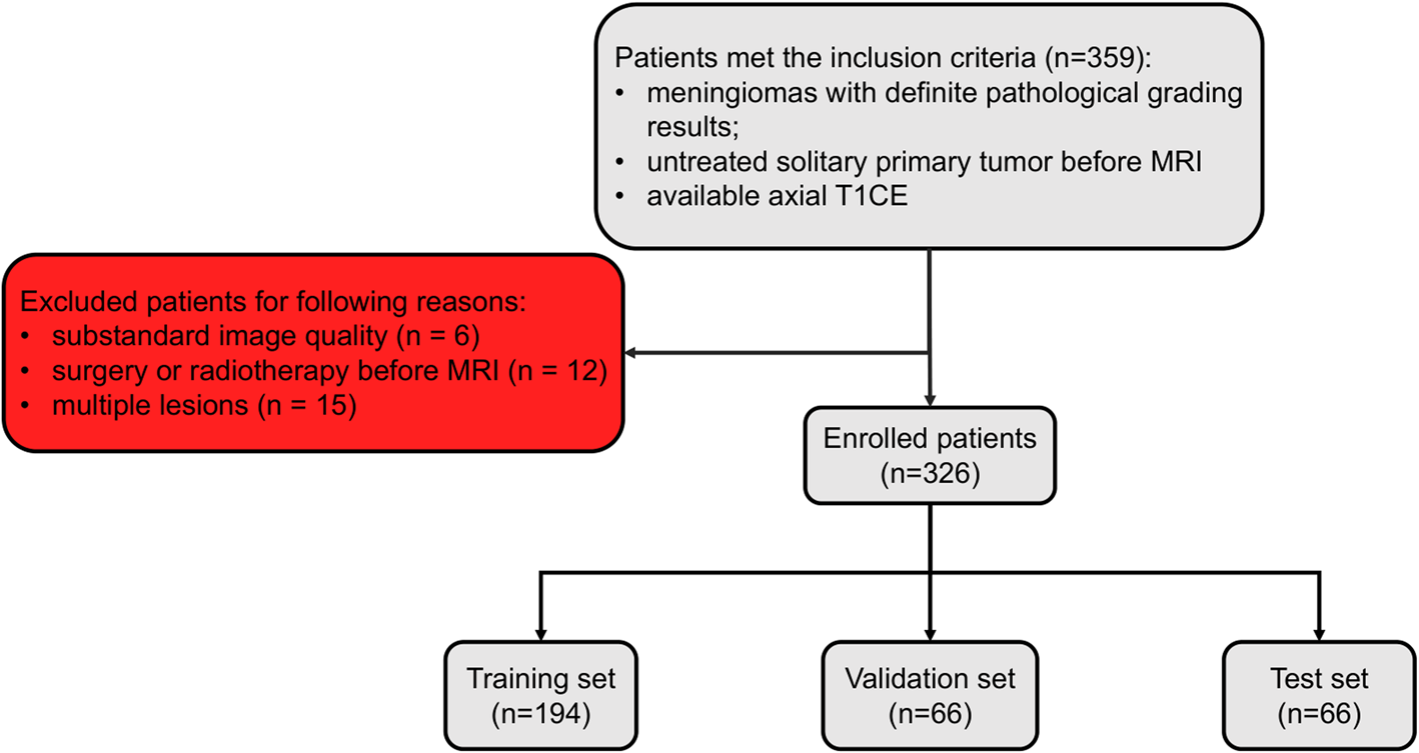
Flowchart of patient selection

**Fig. 2:**
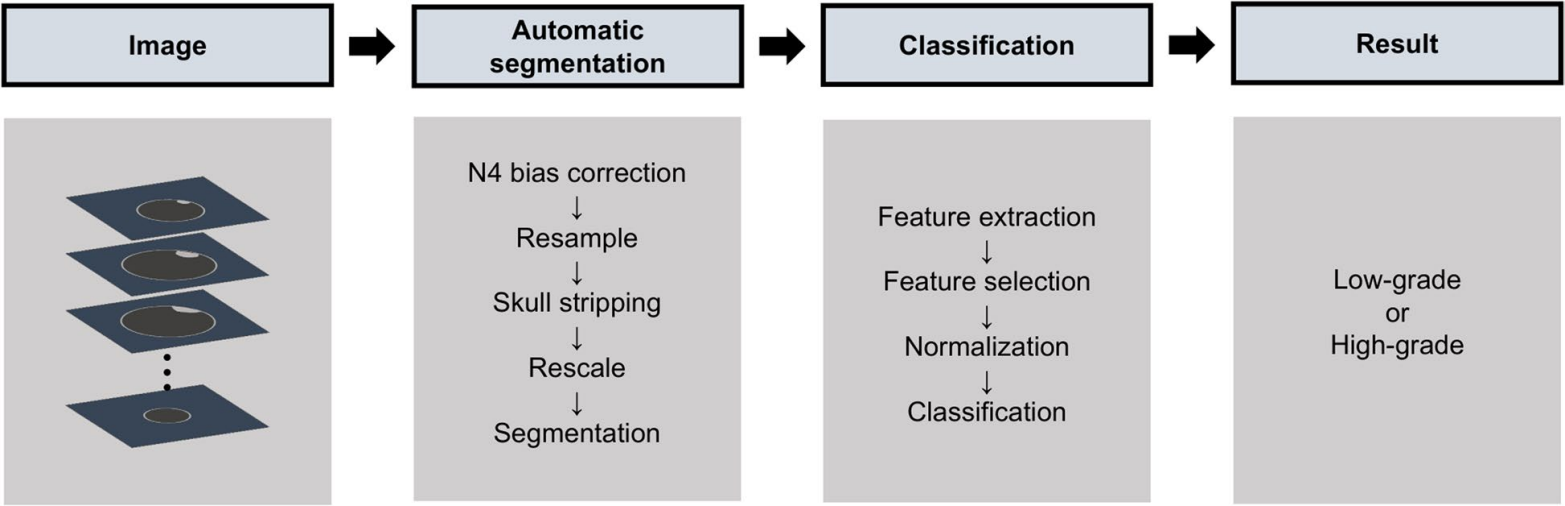
Flowchart of the ML pipeline

Ethical approval was obtained for this retrospective study, and the need for written informed consent was waived. Specific inclusion criteria were listed as follows: (1) patients diagnosed with meningioma by histopathology and with definite WHO grading [18]; (2) previously untreated solitary primary tumor before MRI scans; (3) available axial T1W-CE images; (4) satisfactory image quality and no artifacts for each patient. The exclusion criteria were as follows: (1) A history of relevant treatment before the preoperative MRI examinations; (2) multiple lesions; (3) Patients with metal foreign bodies or claustrophobia. The flowchart of patient selection is displayed in Fig. 1. The flowchart of ML pipeline is presented in Fig. 2.

### MRI acquisition

The examinations were performed by using three 3.0 T MRI scanners (GE Signa HDi, GE Signa HDxt, and GE Discovery MR750w). All MRI examinations were performed within two weeks before surgery. Detailed scanning parameters were: TR = 1665 ms, TE = 25 ms, FOV = 25 cm, slice thickness = 5 mm and matrix = 256 × 256. Each patient was given a dose of 0.1 mmol/kg of gadopentetate dimeglumine at a rate of 3.5 ml/s. Subsequently, the T1W-CE scanning was performed within 250 s.

### Image pre-processing

Before tumor segmentation, the image preprocessing step is first performed, including: (1) resampling to a 1 × 1 × 1 mm^3^ resolution; (2) N4 bias correction using SimpleITK software (version 2.0, https:// www. simpl eitk. org/), it could correct low frequency intensity non-uniformity present in MRI image data known as a bias or gain field; (3) skull stripping was performed using the multi-contrast brain STRipping (MONSTR); (4) resize to 256 × 256 × 16; (5) rescale (range 0–1).

### Manual segmentation

Volumetric regions of interest (VOIs) of training and validation sets were manually segmented by using dedicated ITK-SNAP software (version 3.8.0; www.itksnap.org). VOIs were delineated along the boundary of tumor lesions slice by slice on axial images. The adjacent invasion, necrosis, and peritumoral edema were excluded from the VOIs. Two radiologists (both of who have 15 years of experience in brain MRI interpretation), blinded to the pathological results, drew VOIs jointly. Next, an expert radiologist with 15 years of experience in brain oncology reviewed the results.

### Automatic segmentation

**Fig. 3:**
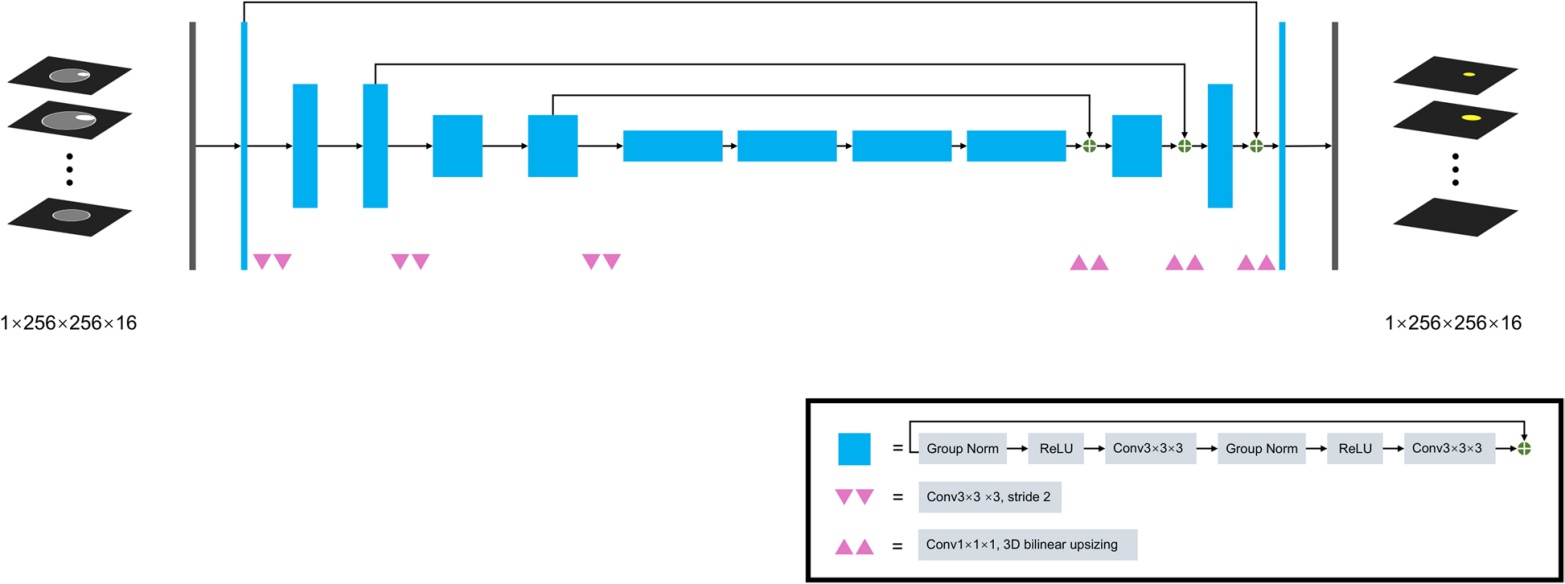
SegResNet architecture

SegResNet, a 3D U-net-like network with a ResNet-like block, was applied to develop the automatic segmentation model, whose code was available on GitHub (https://github.com/Project-MONAI/MONAI) [18]. The architecture of this algorithm is shown in Fig. 3. Manual segmentation is used as the reference standard, and the loss function we use is Dice Loss, which represents the overlap between the two groups and can reduce the impact of imbalanced data.

The equation is displayed as follows:

$$\mathrm{Dice\, Loss}=1-\frac{2|{\text{A}}\cap {\text{B}}|}{|{\text{A}}|+|{\text{B}}|}$$

where A ∩ B is the intersection of two sets, | | represents the number of elements in the set. The batch size was set as 7. We used an AdamW optimizer with an initial learning rate of 0.01 and weight decay of 0.00001. A cosine scheduler with warmup was utilized to dynamically adjust the learning rate according to the epoch. The total epoch and warmup steps were 400 and 40, respectively. The data of the training set were fed into the network to train the model. During training, we implemented data augmentation by randomly mirror flipping and rotating (degree range -45 to 45), which was able to improve the generalization ability. The random probability was 0.5. According to the mean of the Dice coefficient in the validation set, the best model was chosen for automatic segmentation. Our network was developed using the python package “Pytorch” and trained using RTX-2080Ti in the cloud computation platform “AI-Galaxy” (http://www.ai-galaxy.cn/) [19].

### Radiomics analysis

All of the study patients were categorized into low-grade (grade I) and high-grade (grades II and III) groups. Since there were relatively few high-grade meningiomas in this study, we integrated the training and validation sets for radiomics analysis. Radiomics features were extracted from by using the python package “Pyradiomics” (https://pyradiomics.Readthedocs.io/). The scheme of µ ± 3σ (µ: mean; standard deviation) was adapted to reduce noise disturbance [20]. A total of 1688 radiomics features were extracted from each VOIs, including seven categories: first-order, shape, gray-level cooccurrence matrix (GLCM), gray-level dependence matrix (GLDM), gray-level size zone matrix (GLSZM), gray-level run-length matrix (GLRLM), and neighboring gray-tone difference matrix (NGTDM). The filter we used included exponential, square, squareroot, gradient, lbp, logarithm and wavelet. To avoid adverse influence from the different value scales and outliers of the radiomics features, all of them were transformed using robust standardization by the median, 25^th^ percentiles, and 75^th^ percentiles of each feature in training data. The equation is listed as follows: we integrated

$${\text{value}}= \frac{{\text{x}}-{\text{median}}}{{\text{p}}75-{\text{p}}25},$$

where x represents the feature value, while p75 and p25 are the 75^th^ percentile and 25^th^ percentile, respectively.

The intra- and inter-class correlation coefficients (ICCs) was calculated to screen out the stable radiomics features. For sixty cases of meningiomas (30 low-grade and 30 high-grade) were selected randomly for ICC calculation. Two neuroradiologists (neuroradiologists A and B) extracted the radiomics features independently, and neuroradiologist A re-extracted features two weeks later. Finally, the radiomics features with both inter-ICC and intra-ICC are greater than 0.8 were chosen for further analysis. The optimal radiomics feature set was determined using a four-step feature selection method. Firstly, the features with zero variance were discarded. Secondly, the one-way variance of analysis (ANOVA) *P*-value between labels and features was calculated for classification tasks and the features with *P* > 0.05 were excluded. Next, the mutual information value of each feature was calculated by measuring the dependency between feature and target, and the features ranked in the top 100 were retained. Finally, the most effective combination of the radiomics features was selected using recursive feature elimination with tenfold cross-validation (RFECV) based on the AUC mean. Due to the low proportion of high-grade meningioma patients, the synthetic minority oversampling technique (SMOTE) was employed to obtain smoother data for training the model after the preliminary feature selection process in training set. The SMOTE technique is an effective oversampling method commonly employed in medical applications to address the issue of class-imbalanced data. It works by augmenting the number of data instances in the minority class through the generation of synthetic data points from its nearest neighbors using Euclidean distance [21, 22]. Furthermore, EasyEnsemble classifier was also applied to improve the predictive performance considering the imbalance of data distribution, this approach effectively solves the problem of unbalanced data types and reduces the loss of information due to undersampling. The classification models based on lightGBM and EasyEnsembleClassifier algorithm were developed using the optimal feature combination.

### Pipeline

When a preprocessed image was inputted into the DL-based segmentation model, the VOIs could be delineated automatically. Subsequently, a radiomics model for grading meningioma was developed using the radiomics features extracted from automatic segmentation. It was used to predict meningioma grade according to automatic segmentation images. For comparison, a radiomics model for grading meningioma was also constructed based on the radiomics features extracted from manual segmentation.

### Performance evaluation

For the automatic segmentation model, dice coefficient and 95% Hausdorff distance (95HD) were utilized to evaluate the predictive performance of the developed models. Besides, ICC analysis was applied to assess the agreement between radiomic features from manual and automatic segmentations in the test set. The diagnostic performance of the radiomics models was evaluated based on the receiver operating characteristics (ROC) curve. The sensitivity, specificity and accuracy were calculated in the training, validation, and test cohorts, respectively.

### Statistics

In this study, all the statistical analyses were achieved with the scikit-learn package in Python (version 3.8, https://www.python.org/) [23, 24]. The chi-squared test and ANOVA test were used to evaluate the difference among different sets for categorical and continuous variables, respectively. For automatic segmentation model, dice coefficient and 95% Hausdorff distance (95HD) were utilized to evaluate its predictive performance. Besides, ICC analysis was applied to assess the agreement between radiomics features extracted from manual segmentation and automatic segmentation in the test set. The ROC curve was used to evaluate the discriminative performance of the predictive model. The sensitivity, specificity and accuracy were calculated in the training, validation, and test cohorts, respectively. The calibration curve was applied to assess the agreement between the prediction results of the radiomics models and the actual clinical findings, and decision curve analysis (DCA) was used to validate the clinical usefulness of the radiomics models. Comparison of the different radiomics models based on manual segmentation and automatic segmentation using DeLong test. A two-sided *P* value < 0.01 was used as the criterion to indicate a statistically significant difference.

## Results

### Patients’ characteristics

A total of 326 patients who met inclusion criteria from January 2017 to February 2022 were consecutively collected in this retrospective study, composed of 93 males and 233 females (ranging from 34 to 72 years old, median 56). Forty-three patients were assigned to high-grade meningiomas, and 283 patients were confirmed with low-grade meningiomas. All patients were randomly divided into training set, validation set and test set in a 6:2:2 ratio. The training set consisted of 194 patients with meningiomas (170 low-grade, 24 high-grade), the validation set consisted of 66 patients with meningiomas (56 low-grade, 10 high-grade) and the test set consisted of 66 patients with meningiomas (57 low-grade, 9 high-grade). There was no significant difference in age, gender and WHO grade among different sets (both *P* values > 0.01). The independent test group was not used for feature selection and hyperparameter tuning. The baseline characteristics of all patients are listed in Table 1.

**Table 1:** The baseline characteristics of all patients

**Characteristic**	**Training set (*n* = 194)**	**Validation set** **(*n* = 66)**	**Test set** **(*n* = 66)**	**All patients** **(*n* = 326)**	***P* value**
Age, mean ± std	55.4 ± 9.7	53.9 ± 10.0	55.2 ± 9.5	55.0 ± 9.8	> 0.01
Gender, (%)					> 0.01
Male	59 (30.4%)	17 (25.8%)	17 (25.8%)	93 (28.5%)	
Female	135 (69.6%)	49 (74.2%)	49 (74.2%)	233 (71.5%)	
WHO grade, (%)					> 0.01
Low-grade	170 (87.6%)	56 (84.8%)	57 (84.4%)	283 (86.8%)	
High-grade	24 (12.4%)	10 (15.2%)	9 (13.6%)	43 (13.2%)	

### Segmentation performance

**Fig. 4:**
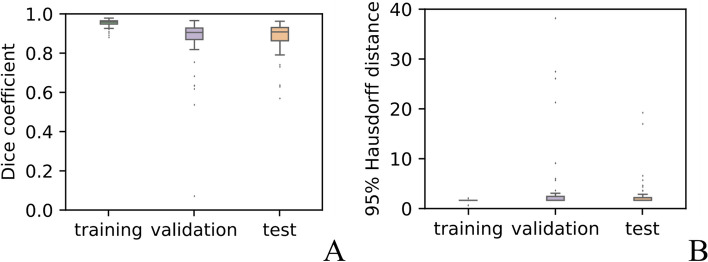
Boxplots for Dice coefficient and 95HD of each automatic segmentation

**Fig. 5:**
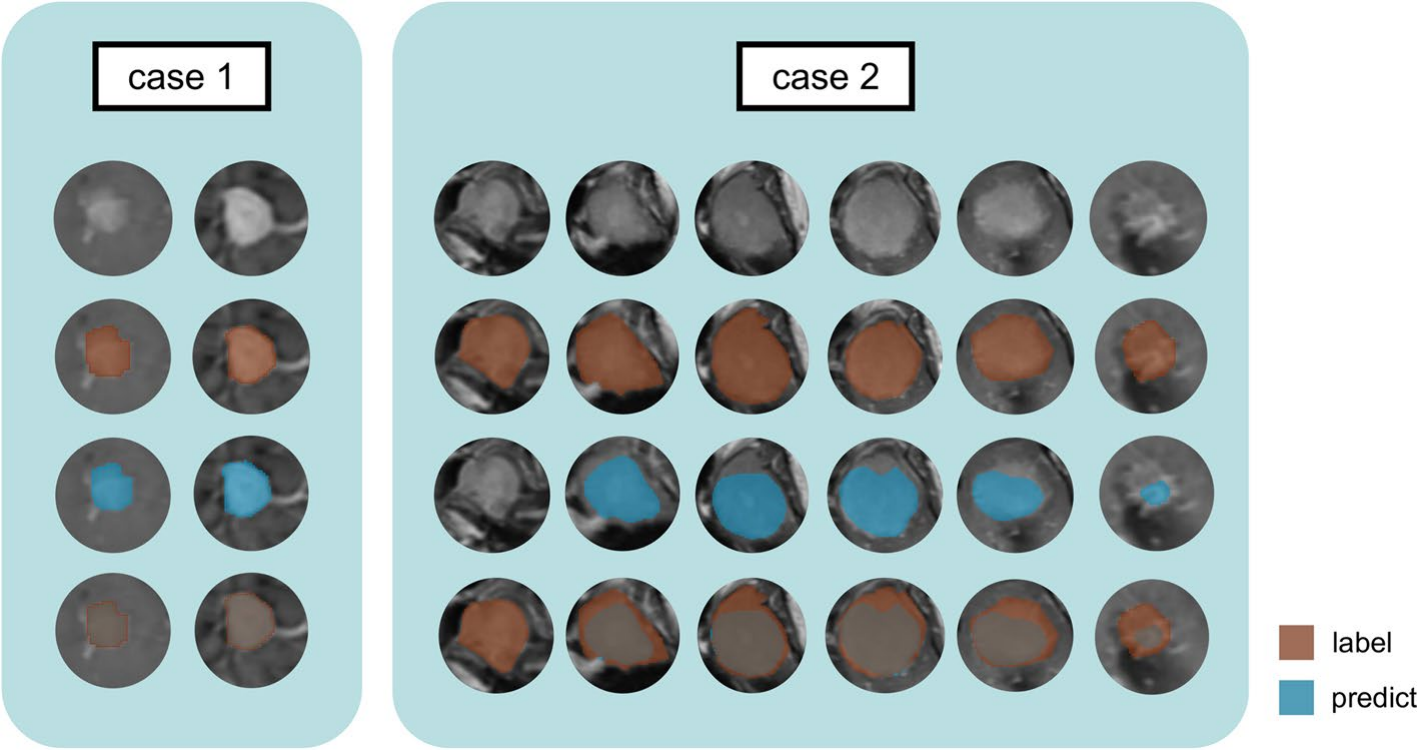
Two representative cases for DLM-based automatic segmentation

The DLM detected meningiomas in all cases. For automatic segmentation, the mean dice coefficient and 95% Hausdorff distance were 0.951 and 0.953 for the training set, respectively; 0.866 and 3.139 for the validation set, respectively; 0.881 and 2.016 for the test set, respectively. Boxplots for the Dice coefficient and 95HD of each automatic segmentation are shown in Fig. 4. Two representative cases for automatic segmentation were displayed in Fig. 5. In the first case, the meningioma was perfectly segmented, while the segmentation result in the second case was not ideal. Overall, the developed deep learning–based segmentation method enables automatic and accurate extraction of meningiomas in the vast majority of cases. Features extracted on manual and automatic segmentation are comparable: the average ICC value was 0.804 (range, 0.636–0.933), and the corresponding results are listed in Table 2. All ICC values were listed in Supplementary material 1.

**Table 2:** Corresponding ICCs of ten selected features between automatic segmentation and manual segmentation

Class	Feature	ICC
Shape	original_shape_Maximum2DDiameterSlice	0.933
	original_shape_MinorAxisLength	0.928
GLCM	wavelet-LLH_glcm_JointAverage	0.930
	wavelet-LLH_glcm_JointEnergy	0.838
	wavelet-LHL_glcm_ClusterTendency	0.910
GLSZM	wavelet-LHH_glszm_ZoneEntropy	0.665
	wavelet-HLL_glszm_ZoneEntropy	0.636
	wavelet-HHL_glszm_GrayLevelNonUniformity	0.703
	wavelet-LLL_glszm_SmallAreaHighGrayLevelEmphasis	0.694
	wavelet-LLL_glszm_HighGrayLevelZoneEmphasis	0.803

### Radiomics performance

**Fig. 6:**
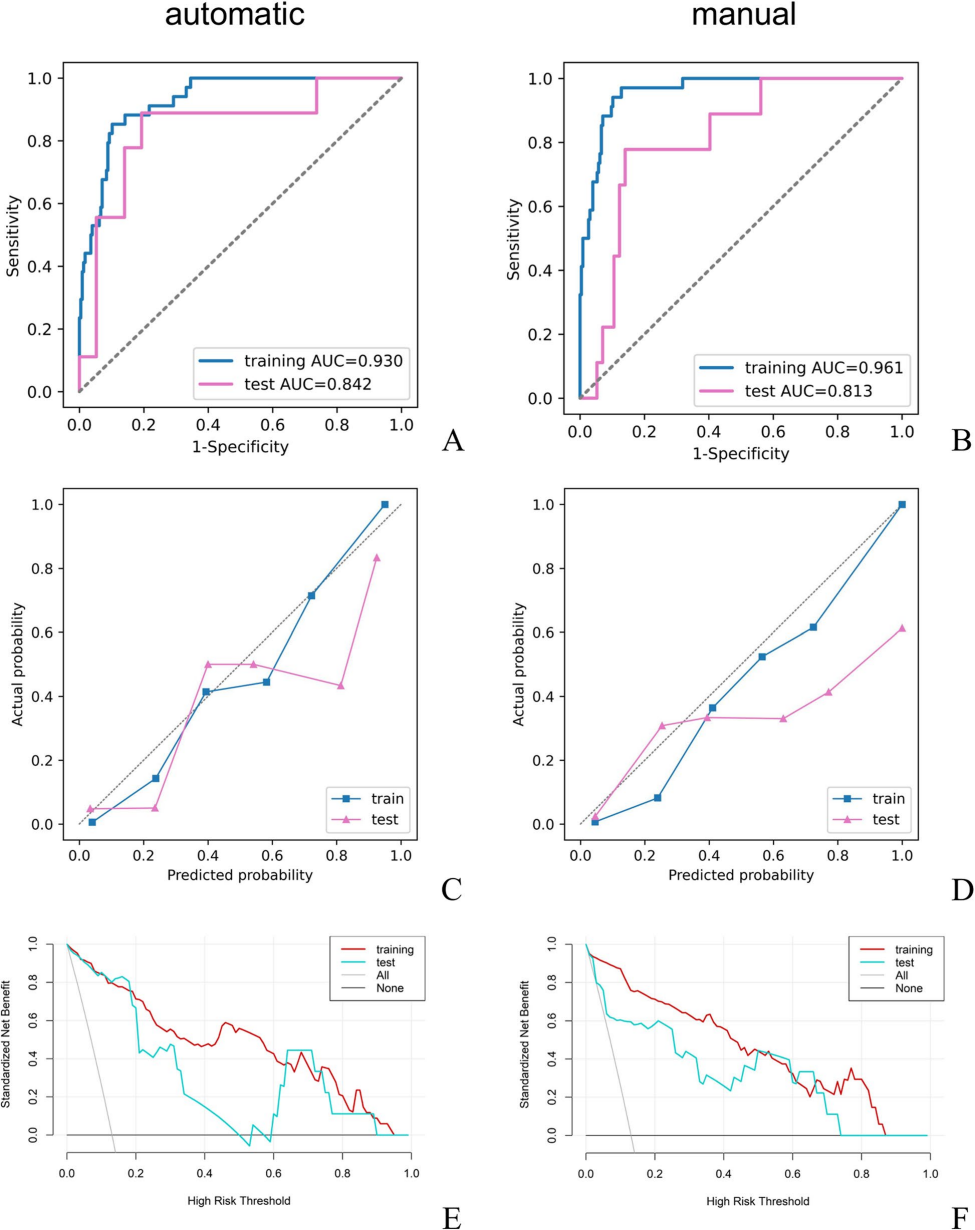
ROC and calibration curves of the radiomics model in the training and test sets. **A** ROC curve of radiomics model based on the radiomics features extracted from automatic segmentation; **B** ROC curve of radiomics model based on the radiomics features extracted from manual segmentation; **C** Calibration curve of radiomics model based on the radiomics features extracted from automatic segmentation; **D** Calibration curve of radiomics model based on the radiomics features extracted from manual segmentation. **E** Decision curve analysis of radiomics model based on the radiomics features extracted from automatic segmentation; **F** Decision curve analysis of radiomics model based on the radiomics features extracted from manual segmentation

In total, 1496 radiomics features demonstrated both inter-ICC and intra-ICC values exceeding 0.80 (listed in Supplementary material 2). Among these features, ten features were finally selected to construct a radiomics model for grading meningiomas. The detailed name of the selected features is shown in Table 2. For meningioma classification, the radiomics model based on the radiomics features extracted from automatic segmentation constructed performed well in grading meningiomas, yielding a sensitivity, specificity, accuracy, and AUC of 0.824 (95% CI: 0.738–0.910), 0.898 (95% CI: 0.760–0.944), 0.888 (95% CI: 0.753–0.921), and 0.930 (95% CI: 0.896–0.952) in the training set, respectively, while these indexes were 0.778 (95% CI: 0.701–0.856), 0.860 (95% CI: 0.722–0.908), 0.848 (95% CI: 0.715–0.903) and 0.842 (95% CI: 0.807–0.895) in the test set, respectively. The radiomics model based on the radiomics features extracted from manual segmentation had a sensitivity, specificity, accuracy, and AUC of 0.941 (95% CI: 0.845–0.969), 0.872 (95% CI: 0.755–0.939), 0.881 (95% CI: 0.750–0.913), and 0.961 (95% CI: 0.904–0.969) in the training set, respectively, while they were 0.778 (95% CI: 0.718–0.859), 0.842 (95% CI: 0.716–0.904), 0.833 (95% CI: 0.709–0.898) and 0.813 (95% CI: 0.799–0.862) in the test set, respectively. The performance of meningioma differentiation on the training and test sets was listed in Table 3, and the corresponding ROC curves of the radiomics models based on automatic segmentation and manual segmentation for meningioma grading were shown in Fig. 6A and B, respectively. The Delong test of radiomics model based on automatic segmentation with radiomics model based on manual segmentation detected no significant differences (*P* = 0.65). Calibration curves (Fig. 6C, D) showed that the predicted probabilities of the radiomics models based on automatic segmentation and manual segmentation were closely aligned with the actual clinical observation in the training set. The agreement between the prediction results of the radiomics models and the actual clinical findings was moderate. The decision curve demonstrated that the results predicted by our radiomics models exhibited favorable clinical usefulness.

**Table 3:** Performance of meningioma differentiation on the training and test sets

Dataset	Sensitivity (95% CI)	Specificity (95% CI)	Accuracy (95% CI)	AUC (95% CI)
Training set (automatic)	0.824 (0.738–0.910)	0.898 (0.760–0.944)	0.888 (0.753–0.921)	0.930 (0.896–0.952)
Test set (automatic)	0.778 (0.701–0.856)	0.860 (0.722–0.908)	0.848 (0.715–0.903)	0.842 (0.807–0.895)
Training set (manual)	0.941 (0.845–0.969)	0.872 (0.755–0.939)	0.881 (0.750–0.913)	0.961 (0.904–0.969)
Test set (manual)	0.778 (0.718–0.859)	0.842 (0.716–0.904)	0.833 (0.709–0.898)	0.813 (0.799–0.862)

## Discussion

The selection of therapeutic strategy and survival prognosis are extremely different for low- and high-grade meningiomas due to their distinct biological characteristics. Given this clinical context, an accurate prediction of meningioma grade has crucial role in guiding treatment decisions. In the present study, we developed a DL-based model for automatic meningioma segmentation in the radiomic analysis of meningioma grading, yielding an AUC of 0.930 and 0.842, respectively, in the training set and test set. Features extracted on manual and automatic segmentation are comparable, and there was a large overlap between these selected radiomics features for the manual and automatic segmentation approaches in the radiomic analysis of meningioma grading.

In the past decades, with the rapid development of ML and computer vision technology, medical image segmentation and classification have made great progress. So far, methods used to segment brain tumors mainly include conventional imaging algorithms, ML-based approaches, and techniques using DL networks. Currently, several (semi-)automatic methods for meningioma segmentation based on brain MR images have been developed. In a study conducted by Hsieh et al., meningiomas were segmented from non-contrast-enhanced MRI images using fuzzy clustering and the region-growing method [25]. Tsai et al. utilized the estimation maximization clustering technique to infer the location of meningiomas and peri-tumor oedemas from T2 axial images [26]. The above traditional approaches yielded moderate efficacy, and their accuracy cannot meet the requirements of clinical routine for meningioma segmentation. To improve performance and to develop an automated segmentation model, DL procedures have been used resulting in significant improvements, notably with the recent developments of convolutional neural networks (CNNs) [27–29]. To perform a pixel-wise segmentation, DL procedures can learn rich features and present a computation advantage over atlas-based strategies [30]. For instance, previous studies have demonstrated that using a DL-based approach to meningiomas greatly improved the segmentation accuracy compared with the traditional technique [31, 32]. Thus, the DL technique could have a big influence on encouraging outcomes on target lesion segmentation and classification, which enables an image-based diagnosis to be more automated. To the best of our knowledge, relatively few studies have been conducted regarding the fully automated detection and segmentation of meningiomas to date. In this study, a DLM was built for automatic segmentation of meningioma based on MRI images, the automatic segmentation model enabled accurate extraction of meningiomas and generate radiomics features that are highly consistent with those obtained using manual segmentation [33]. Furthermore, the radiomics model constructed on features from automatic segmentation can assist in accurately differentiating high-grade meningiomas from low-grade ones in clinical practice.

Compared with other routine sequences in clinical practice, T1W-CE images could show more vivid lesions and boundaries of meningiomas due to abundant blood supply, which facilitated the segment of the tumor to some degree [34]. Laukamp et al. built a dedicated meningioma DLM based on T1W-CE data and evaluated its performance for automated tumor segmentation. Of the 56 meningiomas in the validation group, 55 were detected by the DLM. In these patients, the comparison of the DLM and manual segmentations revealed average dice coefficients of 0.82 ± 0.12 for total lesion volume [31]. In the study by Kang et al., the Sorensen-Dice similarity coefficients of the U-Net for small meningiomas less than 1 cm3 were 0.769 and 0.780 with the internal validation set and external validation set, respectively [35]. We guess that imperfect automation and disappointing performance for small meningiomas of previous automated tools limit their use in routine clinical practice. Although our results demonstrated clinically applicable performance for meningiomas segmentation using SegResNet developed, our prediction performance still needs to be further improved. In the present study, we considered that several challenges still exist in automatic meningiomas segmentation algorithms in the following aspects: First of all, anatomical variations and different MRI equipment resulted in varying imaging data and inconsistent scanning parameters. In addition, the imaging manifestation of different grades and subtypes of meningiomas varies greatly. Finally, large amounts of training data are needed for deep convolutional neural networks to extract complex feature hierarchies through self-learning capabilities because that DLM must work with multiple processing layers and abstraction levels.

Recently, radiomics models based on MRI images have been developed for grading meningiomas in previous works. A meta-analysis summarized eight related radiomics studies, where the pooled AUC of studies employed a test group achieved 0.84 (95% CI: 0.78–0.90), suggesting that radiomics could serve as an effective tool in grading meningiomas [17]. In the present study, the radiomics model constructed on features from automatic segmentation exhibited favorable performance in meningiomas grading on the testing set, yielding a sensitivity, specificity, accuracy, and AUC of 0.778, 0.860, 0.848, and 0.842 in the test set, respectively. The achieved good performance and high reliability of the test set in the present study demonstrated the potential of applying radiomics to assist in accurately differentiating high-grade meningiomas from low-grade ones in clinical practice. The calibration curves for the test set are not good enough, possibly due to limited sample size and imbalanced data. Enlarged datasets are needed to further test the generalizability and clinical usefulness of the constructed ML pipeline. Of note, Verma and his colleagues firstly created a radiomics risk score using radiomic features obtained from different sub-compartments of the tumor habitat (enhancing tumor, peritumoral edema and non-enhancing tumor region, and tumor necrosis regions) to classify patients as low and high risk for poor progression-free survival in response to treatment. Preliminary findings revealed significant associations of prognostic radiomics features with disease-specific histologic attributes [36]. In our analysis, we mainly emphasized the importance of radiomics features derived from the tumor itself, but overlooked the morphologic associations of radiomics features obtained from peritumoral edema with the underlying pathophysiologic processes that drive tumor behavior. In subsequent analysis, we will incorporate radiomics features obtained from peritumoral edema to alleviate this impact.

Among the retained radiomics features, the first-order features describe the distribution of voxel intensities in images. The GLCM features quantify the second-order joint probabilities of images which quantifies the intensity distribution of the gray level at a given offset to extract information about tone homogeneity, linear connection, contrast, and boundaries adjacent to gray zones, as well as complicacy of distribution [37]. The GLSZM features describe gray-level runs in an image. Skewness, as one of the simple parameters, represents the asymmetric distribution of gray levels in the histogram that describes the heterogeneity of lesions [38]. The above features describe the patterns or spatial distribution of voxel intensities within the ROI, which serve as recognized parameters to capture tumor heterogeneity [39]. Indirectly, our findings confirmed that the selected features were all closely related to high-dimensional space information that can hardly be understood by naked-eye examination, which may potentially assist in the differential diagnosis.

Likewise, our study still had several limitations. Firstly, potential selection bias might exist because of the retrospective nature, and the conduct of prospective studies may alleviate this impact; Secondly, the VOIs were delineated along the boundary of meningiomas, suggesting that it might exclude the potential information of peritumoral edema; Thirdly, our data are not collected using scanners from one the same vendor; Fourth, the radiomics model is solely established on the axial T1W-CE images, other sequences, such as T2WI, FLAIR, DWI, ADC images, should be included in subsequent analysis; Finally, thorough validation of this radiomics model will ultimately require application to an external, multi-institutional dataset with a larger cohort of patients. We will add an external validation to provide more sufficient evidence for clinical application in the near future.

## Conclusions

In summary, the developed SegResNet-based segmentation model allowed for automatic and accurate meningioma segmentation from T1W-CE MRI images. The DLM with automatic segmentation demonstrated performance comparable to that of the model with manual segmentation. With respect to meningioma differentiation, our automatic segmentation approach will likely enable the efficient implementation of radiomics for grading meningiomas before surgery and facilitate its clinical application.

### Supplementary Information

**Additional file 1:** **Supplementary Material 1.** All ICCs between features extracted on manual and automatic Segmentation.

**Additional file 2:** **Supplementary Material 2.** All radiomics features with both inter-ICC and intra-ICC values exceeding 0.80.

## Data Availability

The datasets generated during and analyzed during the current study are not publicly available due to patient privacy concerns but are available from the corresponding author on reasonable request.

## Abbreviations

WHO: World Health Organization
DL: Deep Learning
ICC: Intra Class Correlation
DLM: Deep Learning Model
WHO: World Health Organization
MRI: Magnetic Resonance Imaging
T1W-CE: T1-weighted contrast-enhanced
ML: Machine Learning
VOIs: Volumetric Regions of Interest
ANOVA: One-Way Variance of Analysis
95HD: 95% Hausdorff Distance
ROC: Receiver Operating Characteristics
SMOTE: Synthetic Minority Oversampling Technique
AUC: Area Under the Curve
