# Supplementary material for: Deep learning–based automatic segmentation of meningioma from T1-weighted contrast-enhanced MRI for preoperative meningioma differentiation using radiomic features
Source: BMC Med Imaging. 2024 Mar 5;24:56. doi: 10.1186/s12880-024-01218-3 (PMC10916038; doi:10.1186/s12880-024-01218-3)
Supplement: Supplementary file 1 — Additional file 1: Supplementary Material 1. All ICCs between features extracted on manual and automatic Segmentation. [file 12880_2024_1218_MOESM1_ESM.docx]

Supplementaty Material 1 All ICCs between features extracted on manual and automatic segmentation

| Radiomics features | ICCs |
| --- | --- |
| original_shape_Elongation | 0.831958522 |
| original_shape_Flatness | 0.646086802 |
| original_shape_LeastAxisLength | 0.924127811 |
| original_shape_MajorAxisLength | 0.958504999 |
| original_shape_Maximum2DDiameterColumn | 0.953030642 |
| original_shape_Maximum2DDiameterRow | 0.905454738 |
| original_shape_Maximum2DDiameterSlice | 0.933309484 |
| original_shape_Maximum3DDiameter | 0.922398168 |
| original_shape_MeshVolume | 0.935597687 |
| original_shape_MinorAxisLength | 0.928115425 |
| original_shape_Sphericity | 0.847589774 |
| original_shape_SurfaceArea | 0.921404752 |
| original_shape_SurfaceVolumeRatio | 0.971641792 |
| original_shape_VoxelVolume | 0.93558336 |
| original_firstorder_ 10Percentile | 0.51377535 |
| original_firstorder_90Percentile | 0.998442895 |
| original_firstorder_Energy | 0.990912059 |
| original_firstorder_Entropy | 0.94363221 |
| original_firstorder_InterquartileRange | 0.920650066 |
| original_firstorder_Kurtosis | 0.360587431 |
| original_firstorder_Maximum | 0.971341093 |
| original_firstorder_MeanAbsoluteDeviation | 0.875588309 |
| original_firstorder_Mean | 0.964230274 |
| original_firstorder_Median | 0.980276569 |
| original_firstorder_Minimum | 0.409017011 |
| original_firstorder_Range | 0.803152364 |
| original_firstorder_RobustMeanAbsoluteDeviation | 0.794819069 |
| original_firstorder_RootMeanSquared | 0.981682853 |
| original_firstorder_Skewness | 0.444568939 |
| original_firstorder_TotalEnergy | 0.990912059 |
| original_firstorder_Uniformity | 0.964571877 |
| original_firstorder_Variance | 0.809711488 |
| original_glcm_Autocorrelation | 0.510672932 |
| original_glcm_ClusterProminence | 0.444505258 |
| original_glcm_ClusterShade | 0.754796131 |
| original_glcm_ClusterTendency | 0.929115724 |
| original_glcm_Contrast | 0.93530828 |
| original_glcm_Correlation | 0.658111732 |
| original_glcm_DifferenceAverage | 0.975402572 |
| original_glcm_DifferenceEntropy | 0.959022549 |
| original_glcm_DifferenceVariance | 0.929314178 |
| original_glcm_Id | 0.982531336 |
| original_glcm_Idm | 0.980113511 |
| original_glcm_Idmn | 0.911821145 |
| original_glcm_Idn | 0.969455314 |
| original_glcm_Imc1 | 0.675144217 |
| original_glcm_Imc2 | 0.840897005 |
| original_glcm_InverseVariance | 0.985896846 |
| original_glcm_JointAverage | 0.439857124 |
| original_glcm_JointEnergy | 0.976724606 |
| original_glcm_JointEntropy | 0.966770676 |
| original_glcm_MCC | 0.397478324 |
| original_glcm_MaximumProbability | 0.985370265 |
| original_glcm_SumAverage | 0.439857123 |
| original_glcm_SumEntropy | 0.965512584 |

| original_glcm_SumSquares | 0.940019025 |
| --- | --- |
| original_gldm_DependenceEntropy | 0.930036502 |
| original_gldm_DependenceNonUniformity | 0.928420498 |
| original_gldm_DependenceNonUniformityNormalized | 0.803384086 |
| original_gldm_DependenceVariance | 0.903397599 |
| original_gldm_GrayLevelNonUniformity | 0.959106089 |
| original_gldm_GrayLevelVariance | 0.925851275 |
| original_gldm_HighGrayLevelEmphasis | 0.497461737 |
| original_gldm_LargeDependenceEmphasis | 0.977414315 |
| original_gldm_LargeDependenceHighGrayLevelEmphasis | 0.630262544 |
| original_gldm_LargeDependenceLowGrayLevelEmphasis | 0.35577502 |
| original_gldm_LowGrayLevelEmphasis | 0.299834739 |
| original_gldm_SmallDependenceEmphasis | 0.942287658 |
| original_gldm_SmallDependenceHighGrayLevelEmphasis | 0.668126014 |
| original_gldm_SmallDependenceLowGrayLevelEmphasis | 0.843639659 |
| original_glszm_GrayLevelNonUniformity | 0.784685359 |
| original_glszm_GrayLevelNonUniformityNormalized | 0.51088271 |
| original_glszm_GrayLevelVariance | 0.449767898 |
| original_glszm_HighGrayLevelZoneEmphasis | 0.654262549 |
| original_glszm_LargeAreaEmphasis | 0.427967801 |
| original_glszm_LargeAreaHighGrayLevelEmphasis | 0.405944917 |
| original_glszm_LargeAreaLowGrayLevelEmphasis | 0.176066445 |
| original_glszm_LowGrayLevelZoneEmphasis | 0.705952986 |
| original_glszm_SizeZoneNonUniformity | 0.464596467 |
| original_glszm_SizeZoneNonUniformityNormalized | 0.694527988 |
| original_glszm_SmallAreaEmphasis | 0.542527323 |
| original_glszm_SmallAreaHighGrayLevelEmphasis | 0.854900839 |
| original_glszm_SmallAreaLowGrayLevelEmphasis | 0.370355591 |
| original_glszm_ZoneEntropy | 0.786159351 |
| original_glszm_ZonePercentage | 0.92415488 |
| original_glszm_ZoneVariance | 0.718967045 |
| original_glrlm_GrayLevelNonUniformity | 0.955743706 |
| original_glrlm_GrayLevelNonUniformityNormalized | 0.898912187 |
| original_glrlm_GrayLevelVariance | 0.850234294 |
| original_glrlm_HighGrayLevelRunEmphasis | 0.40690087 |
| original_glrlm_LongRunEmphasis | 0.969688697 |
| original_glrlm_LongRunHighGrayLevelEmphasis | 0.738316477 |
| original_glrlm_LongRunLowGrayLevelEmphasis | 0.508433302 |
| original_glrlm_LowGrayLevelRunEmphasis | 0.291978257 |
| original_glrlm_RunEntropy | 0.743995621 |
| original_glrlm_RunLengthNonUniformity | 0.94206756 |
| original_glrlm_RunLengthNonUniformityNormalized | 0.694980027 |
| original_glrlm_RunPercentage | 0.81406626 |
| original_glrlm_RunVariance | 0.924763252 |
| original_glrlm_ShortRunEmphasis | 0.849393735 |
| original_glrlm_ShortRunHighGrayLevelEmphasis | 0.618575422 |
| original_glrlm_ShortRunLowGrayLevelEmphasis | 0.435313829 |
| original_ngtdm_Busyness | 0.280301121 |
| original_ngtdm_Coarseness | 0.517088001 |
| original_ngtdm_Complexity | 0.743079495 |
| original_ngtdm_Contrast | 0.880775518 |
| original_ngtdm_Strength | 0.543371307 |
| exponential_firstorder_ 10Percentile | 0.501691567 |
| exponential_firstorder_90Percentile | 0.9932892 |
| exponential_firstorder_Energy | 0.984243213 |
| exponential_firstorder_Entropy | 0.995843946 |

| exponential_firstorder_InterquartileRange | 0.924792892 |
| --- | --- |
| exponential_firstorder_Kurtosis | 0.685474534 |
| exponential_firstorder_Maximum | 0.922536482 |
| exponential_firstorder_MeanAbsoluteDeviation | 0.97093648 |
| exponential_firstorder_Mean | 0.952681373 |
| exponential_firstorder_Median | 0.953885153 |
| exponential_firstorder_Minimum | 0.380369652 |
| exponential_firstorder_Range | 0.891798301 |
| exponential_firstorder_RobustMeanAbsoluteDeviation | 0.947617857 |
| exponential_firstorder_RootMeanSquared | 0.968921681 |
| exponential_firstorder_Skewness | 0.643681964 |
| exponential_firstorder_TotalEnergy | 0.984243213 |
| exponential_firstorder_Uniformity | 0.996441051 |
| exponential_firstorder_Variance | 0.983062831 |
| exponential_glcm_Autocorrelation | 0.996734558 |
| exponential_glcm_ClusterProminence | 0.996882687 |
| exponential_glcm_ClusterShade | 0.992066613 |
| exponential_glcm_ClusterTendency | 0.997500794 |
| exponential_glcm_Contrast | 0.995224417 |
| exponential_glcm_Correlation | 0.731713184 |
| exponential_glcm_DifferenceAverage | 0.995225903 |
| exponential_glcm_DifferenceEntropy | 0.991310276 |
| exponential_glcm_DifferenceVariance | 0.993379953 |
| exponential_glcm_Id | 0.995226395 |
| exponential_glcm_Idm | 0.995226199 |
| exponential_glcm_Idmn | 0.995289632 |
| exponential_glcm_Idn | 0.995260682 |
| exponential_glcm_Imc1 | 0.933811417 |
| exponential_glcm_Imc2 | 0.986999614 |
| exponential_glcm_InverseVariance | 0.995227192 |
| exponential_glcm_JointAverage | 0.99652431 |
| exponential_glcm_JointEnergy | 0.99679089 |
| exponential_glcm_JointEntropy | 0.995684016 |
| exponential_glcm_MCC | 0.689431862 |
| exponential_glcm_MaximumProbability | 0.997002131 |
| exponential_glcm_SumAverage | 0.99652431 |
| exponential_glcm_SumEntropy | 0.994640505 |
| exponential_glcm_SumSquares | 0.997427962 |
| exponential_gldm_DependenceEntropy | 0.903984726 |
| exponential_gldm_DependenceNonUniformity | 0.915352534 |
| exponential_gldm_DependenceNonUniformityNormalized | 0.782664173 |
| exponential_gldm_DependenceVariance | 0.855407375 |
| exponential_gldm_GrayLevelNonUniformity | 0.936195355 |
| exponential_gldm_GrayLevelVariance | 0.996441207 |
| exponential_gldm_HighGrayLevelEmphasis | 0.99236425 |
| exponential_gldm_LargeDependenceEmphasis | 0.974926698 |
| exponential_gldm_LargeDependenceHighGrayLevelEmphasis | 0.969607614 |
| exponential_gldm_LargeDependenceLowGrayLevelEmphasis | 0.976936727 |
| exponential_gldm_LowGrayLevelEmphasis | 0.992364152 |
| exponential_gldm_SmallDependenceEmphasis | 0.94082302 |
| exponential_gldm_SmallDependenceHighGrayLevelEmphasis | 0.915321813 |
| exponential_gldm_SmallDependenceLowGrayLevelEmphasis | 0.959497152 |
| exponential_glszm_GrayLevelNonUniformity | 0.980224029 |
| exponential_glszm_GrayLevelNonUniformityNormalized | 0.746536423 |
| exponential_glszm_GrayLevelVariance | 0.747389836 |
| exponential_glszm_HighGrayLevelZoneEmphasis | 0.859779772 |

| exponential_glszm_LargeAreaEmphasis | 0.824807158 |
| --- | --- |
| exponential_glszm_LargeAreaHighGrayLevelEmphasis | 0.824654572 |
| exponential_glszm_LargeAreaLowGrayLevelEmphasis | 0.824846029 |
| exponential_glszm_LowGrayLevelZoneEmphasis | 0.856848088 |
| exponential_glszm_SizeZoneNonUniformity | 0.962434999 |
| exponential_glszm_SizeZoneNonUniformityNormalized | 0.807300501 |
| exponential_glszm_SmallAreaEmphasis | 0.756389792 |
| exponential_glszm_SmallAreaHighGrayLevelEmphasis | 0.768333324 |
| exponential_glszm_SmallAreaLowGrayLevelEmphasis | 0.727898936 |
| exponential_glszm_ZoneEntropy | 0.878015569 |
| exponential_glszm_ZonePercentage | 0.942557678 |
| exponential_glszm_ZoneVariance | 0.466614818 |
| exponential_glrlm_GrayLevelNonUniformity | 0.920299009 |
| exponential_glrlm_GrayLevelNonUniformityNormalized | 0.987198512 |
| exponential_glrlm_GrayLevelVariance | 0.987212268 |
| exponential_glrlm_HighGrayLevelRunEmphasis | 0.988285913 |
| exponential_glrlm_LongRunEmphasis | 0.946389216 |
| exponential_glrlm_LongRunHighGrayLevelEmphasis | 0.943578801 |
| exponential_glrlm_LongRunLowGrayLevelEmphasis | 0.947119215 |
| exponential_glrlm_LowGrayLevelRunEmphasis | 0.988280907 |
| exponential_glrlm_RunEntropy | 0.748757868 |
| exponential_glrlm_RunLengthNonUniformity | 0.884437749 |
| exponential_glrlm_RunLengthNonUniformityNormalized | 0.66010626 |
| exponential_glrlm_RunPercentage | 0.785137136 |
| exponential_glrlm_RunVariance | 0.940476615 |
| exponential_glrlm_ShortRunEmphasis | 0.834114822 |
| exponential_glrlm_ShortRunHighGrayLevelEmphasis | 0.972352275 |
| exponential_glrlm_ShortRunLowGrayLevelEmphasis | 0.667061168 |
| exponential_ngtdm_Busyness | 0.817864396 |
| exponential_ngtdm_Coarseness | 0.756189243 |
| exponential_ngtdm_Complexity | 0.995024803 |
| exponential_ngtdm_Contrast | 0.991587329 |
| exponential_ngtdm_Strength | 0.53224217 |
| gradient_firstorder_ 10Percentile | 0.945318046 |
| gradient_firstorder_90Percentile | 0.99360908 |
| gradient_firstorder_Energy | 0.967850399 |
| gradient_firstorder_Entropy | 0.473181444 |
| gradient_firstorder_InterquartileRange | 0.96410461 |
| gradient_firstorder_Kurtosis | 0.741364783 |
| gradient_firstorder_Maximum | 0.910684978 |
| gradient_firstorder_MeanAbsoluteDeviation | 0.982867486 |
| gradient_firstorder_Mean | 0.991011455 |
| gradient_firstorder_Median | 0.982268874 |
| gradient_firstorder_Minimum | 0.920776697 |
| gradient_firstorder_Range | 0.913179192 |
| gradient_firstorder_RobustMeanAbsoluteDeviation | 0.975164242 |
| gradient_firstorder_RootMeanSquared | 0.991714611 |
| gradient_firstorder_Skewness | 0.857583179 |
| gradient_firstorder_TotalEnergy | 0.967850399 |
| gradient_firstorder_Uniformity | 0.444220713 |
| gradient_firstorder_Variance | 0.989311628 |
| gradient_glcm_Autocorrelation | 0.706370671 |
| gradient_glcm_ClusterProminence | 0.655640984 |
| gradient_glcm_ClusterShade | 0.685930507 |
| gradient_glcm_ClusterTendency | 0.702364345 |
| gradient_glcm_Contrast | 0.718764178 |

| gradient_glcm_Correlation | 0.703618282 |
| --- | --- |
| gradient_glcm_DifferenceAverage | 0.718764178 |
| gradient_glcm_DifferenceEntropy | 0.732266415 |
| gradient_glcm_DifferenceVariance | 0.719003943 |
| gradient_glcm_Id | 0.718764146 |
| gradient_glcm_Idm | 0.718764146 |
| gradient_glcm_Idmn | 0.718763888 |
| gradient_glcm_Idn | 0.71876384 |
| gradient_glcm_Imc1 | 0.725389746 |
| gradient_glcm_Imc2 | 0.540548453 |
| gradient_glcm_InverseVariance | 0.718764178 |
| gradient_glcm_JointAverage | 0.710588954 |
| gradient_glcm_JointEnergy | 0.71492259 |
| gradient_glcm_JointEntropy | 0.726381478 |
| gradient_glcm_MCC | 0.639965324 |
| gradient_glcm_MaximumProbability | 0.714735796 |
| gradient_glcm_SumAverage | 0.710589089 |
| gradient_glcm_SumEntropy | 0.726896893 |
| gradient_glcm_SumSquares | 0.710732854 |
| gradient_gldm_DependenceEntropy | 0.497024302 |
| gradient_gldm_DependenceNonUniformity | 0.912818544 |
| gradient_gldm_DependenceNonUniformityNormalized | 0.507583832 |
| gradient_gldm_DependenceVariance | 0.735015659 |
| gradient_gldm_GrayLevelNonUniformity | 0.935581341 |
| gradient_gldm_GrayLevelVariance | 0.444220553 |
| gradient_gldm_HighGrayLevelEmphasis | 0.443878486 |
| gradient_gldm_LargeDependenceEmphasis | 0.965217428 |
| gradient_gldm_LargeDependenceHighGrayLevelEmphasis | 0.965217904 |
| gradient_gldm_LargeDependenceLowGrayLevelEmphasis | 0.965217309 |
| gradient_gldm_LowGrayLevelEmphasis | 0.443878371 |
| gradient_gldm_SmallDependenceEmphasis | 0.967761327 |
| gradient_gldm_SmallDependenceHighGrayLevelEmphasis | 0.940760126 |
| gradient_gldm_SmallDependenceLowGrayLevelEmphasis | 0.969443986 |
| gradient_glszm_GrayLevelNonUniformity | 0.019722098 |
| gradient_glszm_GrayLevelNonUniformityNormalized | 0.653337406 |
| gradient_glszm_GrayLevelVariance | 0.653337406 |
| gradient_glszm_HighGrayLevelZoneEmphasis | 0.609039354 |
| gradient_glszm_LargeAreaEmphasis | 0.831516907 |
| gradient_glszm_LargeAreaHighGrayLevelEmphasis | 0.831516907 |
| gradient_glszm_LargeAreaLowGrayLevelEmphasis | 0.831516907 |
| gradient_glszm_LowGrayLevelZoneEmphasis | 0.609039354 |
| gradient_glszm_SizeZoneNonUniformity | 0.450953678 |
| gradient_glszm_SizeZoneNonUniformityNormalized | 0.450953678 |
| gradient_glszm_SmallAreaEmphasis | 0.512050098 |
| gradient_glszm_SmallAreaHighGrayLevelEmphasis | 0.51415861 |
| gradient_glszm_SmallAreaLowGrayLevelEmphasis | 0.48815034 |
| gradient_glszm_ZoneEntropy | 0.435892549 |
| gradient_glszm_ZonePercentage | 0.920245336 |
| gradient_glszm_ZoneVariance | 0.752234088 |
| gradient_glrlm_GrayLevelNonUniformity | 0.920979754 |
| gradient_glrlm_GrayLevelNonUniformityNormalized | 0.423355009 |
| gradient_glrlm_GrayLevelVariance | 0.423354986 |
| gradient_glrlm_HighGrayLevelRunEmphasis | 0.416348921 |
| gradient_glrlm_LongRunEmphasis | 0.935755219 |
| gradient_glrlm_LongRunHighGrayLevelEmphasis | 0.935754275 |
| gradient_glrlm_LongRunLowGrayLevelEmphasis | 0.935755455 |

| gradient_glrlm_LowGrayLevelRunEmphasis | 0.416348912 |
| --- | --- |
| gradient_glrlm_RunEntropy | 0.803239695 |
| gradient_glrlm_RunLengthNonUniformity | 0.808557458 |
| gradient_glrlm_RunLengthNonUniformityNormalized | 0.600629769 |
| gradient_glrlm_RunPercentage | 0.567889956 |
| gradient_glrlm_RunVariance | 0.927841949 |
| gradient_glrlm_ShortRunEmphasis | 0.396468342 |
| gradient_glrlm_ShortRunHighGrayLevelEmphasis | 0.39297985 |
| gradient_glrlm_ShortRunLowGrayLevelEmphasis | 0.39734595 |
| gradient_ngtdm_Busyness | 0.647750625 |
| gradient_ngtdm_Coarseness | 0.646098105 |
| gradient_ngtdm_Complexity | 0.489707819 |
| gradient_ngtdm_Contrast | 0.276478451 |
| gradient_ngtdm_Strength | 0.551511618 |
| lbp-2D_firstorder_ 10Percentile | 0.818453576 |
| lbp-2D_firstorder_90Percentile | 0.834167513 |
| lbp-2D_firstorder_Energy | 0.890509069 |
| lbp-2D_firstorder_Entropy | 0.935369412 |
| lbp-2D_firstorder_InterquartileRange | 0.784215035 |
| lbp-2D_firstorder_Kurtosis | 0.898789673 |
| lbp-2D_firstorder_Maximum | 0.890509069 |
| lbp-2D_firstorder_MeanAbsoluteDeviation | 0.913767779 |
| lbp-2D_firstorder_Mean | 0.750344786 |
| lbp-2D_firstorder_Median | 0.75347866 |
| lbp-2D_firstorder_Minimum | 0.94122066 |
| lbp-2D_firstorder_Range | 0.75156498 |
| lbp-2D_firstorder_RobustMeanAbsoluteDeviation | 0.835941201 |
| lbp-2D_firstorder_RootMeanSquared | 0.845883348 |
| lbp-2D_firstorder_Skewness | 0.906966369 |
| lbp-2D_firstorder_TotalEnergy | 0.890509069 |
| lbp-2D_firstorder_Uniformity | 0.933917029 |
| lbp-2D_firstorder_Variance | 0.935561863 |
| lbp-2D_glcm_Autocorrelation | 0.944679244 |
| lbp-2D_glcm_ClusterProminence | 0.611978506 |
| lbp-2D_glcm_ClusterShade | 0.847709276 |
| lbp-2D_glcm_ClusterTendency | 0.856030426 |
| lbp-2D_glcm_Contrast | 0.915266103 |
| lbp-2D_glcm_Correlation | 0.237486005 |
| lbp-2D_glcm_DifferenceAverage | 0.915266103 |
| lbp-2D_glcm_DifferenceEntropy | 0.958638162 |
| lbp-2D_glcm_DifferenceVariance | 0.95812849 |
| lbp-2D_glcm_Id | 0.915266102 |
| lbp-2D_glcm_Idm | 0.915266102 |
| lbp-2D_glcm_Idmn | 0.915266101 |
| lbp-2D_glcm_Idn | 0.915266103 |
| lbp-2D_glcm_Imc1 | 0.782475869 |
| lbp-2D_glcm_Imc2 | 0.884585501 |
| lbp-2D_glcm_InverseVariance | 0.915266103 |
| lbp-2D_glcm_JointAverage | 0.951279135 |
| lbp-2D_glcm_JointEnergy | 0.955278221 |
| lbp-2D_glcm_JointEntropy | 0.956919527 |
| lbp-2D_glcm_MCC | 0.89036481 |
| lbp-2D_glcm_MaximumProbability | 0.955204967 |
| lbp-2D_glcm_SumAverage | 0.951279135 |
| lbp-2D_glcm_SumEntropy | 0.930384136 |
| lbp-2D_glcm_SumSquares | 0.949682078 |

| lbp-2D_gldm_DependenceEntropy | 0.923437915 |
| --- | --- |
| lbp-2D_gldm_DependenceNonUniformity | 0.94151371 |
| lbp-2D_gldm_DependenceNonUniformityNormalized | 0.955090404 |
| lbp-2D_gldm_DependenceVariance | 0.937679572 |
| lbp-2D_gldm_GrayLevelNonUniformity | 0.945540887 |
| lbp-2D_gldm_GrayLevelVariance | 0.933917029 |
| lbp-2D_gldm_HighGrayLevelEmphasis | 0.922759788 |
| lbp-2D_gldm_LargeDependenceEmphasis | 0.957831762 |
| lbp-2D_gldm_LargeDependenceHighGrayLevelEmphasis | 0.922635503 |
| lbp-2D_gldm_LargeDependenceLowGrayLevelEmphasis | 0.957470369 |
| lbp-2D_gldm_LowGrayLevelEmphasis | 0.922759787 |
| lbp-2D_gldm_SmallDependenceEmphasis | 0.907535404 |
| lbp-2D_gldm_SmallDependenceHighGrayLevelEmphasis | 0.860905701 |
| lbp-2D_gldm_SmallDependenceLowGrayLevelEmphasis | 0.921088529 |
| lbp-2D_glszm_GrayLevelNonUniformity | 0.681186388 |
| lbp-2D_glszm_GrayLevelNonUniformityNormalized | 0.465020604 |
| lbp-2D_glszm_GrayLevelVariance | 0.465020604 |
| lbp-2D_glszm_HighGrayLevelZoneEmphasis | 0.336637509 |
| lbp-2D_glszm_LargeAreaEmphasis | 0.877571517 |
| lbp-2D_glszm_LargeAreaHighGrayLevelEmphasis | 0.841359664 |
| lbp-2D_glszm_LargeAreaLowGrayLevelEmphasis | 0.890766642 |
| lbp-2D_glszm_LowGrayLevelZoneEmphasis | 0.336637509 |
| lbp-2D_glszm_SizeZoneNonUniformity | 0.509651133 |
| lbp-2D_glszm_SizeZoneNonUniformityNormalized | 0.185608224 |
| lbp-2D_glszm_SmallAreaEmphasis | 0.027366739 |
| lbp-2D_glszm_SmallAreaHighGrayLevelEmphasis | 0.019275902 |
| lbp-2D_glszm_SmallAreaLowGrayLevelEmphasis | 0.091282278 |
| lbp-2D_glszm_ZoneEntropy | 0.377182288 |
| lbp-2D_glszm_ZonePercentage | 0.920840165 |
| lbp-2D_glszm_ZoneVariance | 0.891700781 |
| lbp-2D_glrlm_GrayLevelNonUniformity | 0.940393034 |
| lbp-2D_glrlm_GrayLevelNonUniformityNormalized | 0.940571573 |
| lbp-2D_glrlm_GrayLevelVariance | 0.940571576 |
| lbp-2D_glrlm_HighGrayLevelRunEmphasis | 0.917797238 |
| lbp-2D_glrlm_LongRunEmphasis | 0.691841207 |
| lbp-2D_glrlm_LongRunHighGrayLevelEmphasis | 0.358422229 |
| lbp-2D_glrlm_LongRunLowGrayLevelEmphasis | 0.754124651 |
| lbp-2D_glrlm_LowGrayLevelRunEmphasis | 0.917797238 |
| lbp-2D_glrlm_RunEntropy | 0.548740798 |
| lbp-2D_glrlm_RunLengthNonUniformity | 0.928191184 |
| lbp-2D_glrlm_RunLengthNonUniformityNormalized | 0.097959266 |
| lbp-2D_glrlm_RunPercentage | 0.31403142 |
| lbp-2D_glrlm_RunVariance | 0.73522079 |
| lbp-2D_glrlm_ShortRunEmphasis | 0.181887114 |
| lbp-2D_glrlm_ShortRunHighGrayLevelEmphasis | 0.668280247 |
| lbp-2D_glrlm_ShortRunLowGrayLevelEmphasis | 0.071854133 |
| lbp-2D_ngtdm_Busyness | 0.659639368 |
| lbp-2D_ngtdm_Coarseness | 0.920491177 |
| lbp-2D_ngtdm_Complexity | 0.943225367 |
| lbp-2D_ngtdm_Contrast | 0.947489247 |
| lbp-2D_ngtdm_Strength | 0.925264294 |
| lbp-3D-m1_firstorder_ 10Percentile | 0.959806948 |
| lbp-3D-m1_firstorder_90Percentile | 0.886827448 |
| lbp-3D-m1_firstorder_Energy | 0.924959503 |
| lbp-3D-m1_firstorder_Entropy | 0.907342922 |
| lbp-3D-m1_firstorder_InterquartileRange | 0.944897959 |

| lbp-3D-m1_firstorder_Kurtosis | 0.617724684 |
| --- | --- |
| lbp-3D-m1_firstorder_Maximum | 0.811848153 |
| lbp-3D-m1_firstorder_MeanAbsoluteDeviation | 0.927532583 |
| lbp-3D-m1_firstorder_Mean | 0.962918727 |
| lbp-3D-m1_firstorder_Median | 0.959598535 |
| lbp-3D-m1_firstorder_Minimum | 0.977438389 |
| lbp-3D-m1_firstorder_Range | 0.823114572 |
| lbp-3D-m1_firstorder_RobustMeanAbsoluteDeviation | 0.952781173 |
| lbp-3D-m1_firstorder_RootMeanSquared | 0.950840934 |
| lbp-3D-m1_firstorder_Skewness | 0.781634131 |
| lbp-3D-m1_firstorder_TotalEnergy | 0.924959503 |
| lbp-3D-m1_firstorder_Uniformity | 0.899254482 |
| lbp-3D-m1_firstorder_Variance | 0.927397902 |
| lbp-3D-m1_glcm_Autocorrelation | 0.964202695 |
| lbp-3D-m1_glcm_ClusterProminence | 0.832013371 |
| lbp-3D-m1_glcm_ClusterShade | 0.621147801 |
| lbp-3D-m1_glcm_ClusterTendency | 0.889708284 |
| lbp-3D-m1_glcm_Contrast | 0.90225894 |
| lbp-3D-m1_glcm_Correlation | 0.748048827 |
| lbp-3D-m1_glcm_DifferenceAverage | 0.859264333 |
| lbp-3D-m1_glcm_DifferenceEntropy | 0.940378522 |
| lbp-3D-m1_glcm_DifferenceVariance | 0.943986563 |
| lbp-3D-m1_glcm_Id | 0.825850673 |
| lbp-3D-m1_glcm_Idm | 0.832119147 |
| lbp-3D-m1_glcm_Idmn | 0.642998184 |
| lbp-3D-m1_glcm_Idn | 0.712653107 |
| lbp-3D-m1_glcm_Imc1 | 0.913332574 |
| lbp-3D-m1_glcm_Imc2 | 0.831299717 |
| lbp-3D-m1_glcm_InverseVariance | 0.940454697 |
| lbp-3D-m1_glcm_JointAverage | 0.950127187 |
| lbp-3D-m1_glcm_JointEnergy | 0.911874298 |
| lbp-3D-m1_glcm_JointEntropy | 0.943717056 |
| lbp-3D-m1_glcm_MCC | 0.85215452 |
| lbp-3D-m1_glcm_MaximumProbability | 0.929648146 |
| lbp-3D-m1_glcm_SumAverage | 0.950127188 |
| lbp-3D-m1_glcm_SumEntropy | 0.926444401 |
| lbp-3D-m1_glcm_SumSquares | 0.916269599 |
| lbp-3D-m1_gldm_DependenceEntropy | 0.96197298 |
| lbp-3D-m1_gldm_DependenceNonUniformity | 0.939888436 |
| lbp-3D-m1_gldm_DependenceNonUniformityNormalized | 0.948430167 |
| lbp-3D-m1_gldm_DependenceVariance | 0.867281781 |
| lbp-3D-m1_gldm_GrayLevelNonUniformity | 0.932825137 |
| lbp-3D-m1_gldm_GrayLevelVariance | 0.914292237 |
| lbp-3D-m1_gldm_HighGrayLevelEmphasis | 0.955798795 |
| lbp-3D-m1_gldm_LargeDependenceEmphasis | 0.899044664 |
| lbp-3D-m1_gldm_LargeDependenceHighGrayLevelEmphasis | 0.927288017 |
| lbp-3D-m1_gldm_LargeDependenceLowGrayLevelEmphasis | 0.966815392 |
| lbp-3D-m1_gldm_LowGrayLevelEmphasis | 0.973347154 |
| lbp-3D-m1_gldm_SmallDependenceEmphasis | 0.844785582 |
| lbp-3D-m1_gldm_SmallDependenceHighGrayLevelEmphasis | 0.778011483 |
| lbp-3D-m1_gldm_SmallDependenceLowGrayLevelEmphasis | 0.936058724 |
| lbp-3D-m1_glszm_GrayLevelNonUniformity | 0.905425011 |
| lbp-3D-m1_glszm_GrayLevelNonUniformityNormalized | 0.73328486 |
| lbp-3D-m1_glszm_GrayLevelVariance | 0.896104427 |
| lbp-3D-m1_glszm_HighGrayLevelZoneEmphasis | 0.829041436 |
| lbp-3D-m1_glszm_LargeAreaEmphasis | 0.940877246 |

| lbp-3D-m1_glszm_LargeAreaHighGrayLevelEmphasis | 0.939001692 |
| --- | --- |
| lbp-3D-m1_glszm_LargeAreaLowGrayLevelEmphasis | 0.927976108 |
| lbp-3D-m1_glszm_LowGrayLevelZoneEmphasis | 0.752604496 |
| lbp-3D-m1_glszm_SizeZoneNonUniformity | 0.876077399 |
| lbp-3D-m1_glszm_SizeZoneNonUniformityNormalized | 0.660185492 |
| lbp-3D-m1_glszm_SmallAreaEmphasis | 0.535516956 |
| lbp-3D-m1_glszm_SmallAreaHighGrayLevelEmphasis | 0.730831088 |
| lbp-3D-m1_glszm_SmallAreaLowGrayLevelEmphasis | 0.797446215 |
| lbp-3D-m1_glszm_ZoneEntropy | 0.896563002 |
| lbp-3D-m1_glszm_ZonePercentage | 0.808692132 |
| lbp-3D-m1_glszm_ZoneVariance | 0.942446159 |
| lbp-3D-m1_glrlm_GrayLevelNonUniformity | 0.937102437 |
| lbp-3D-m1_glrlm_GrayLevelNonUniformityNormalized | 0.955575779 |
| lbp-3D-m1_glrlm_GrayLevelVariance | 0.916819035 |
| lbp-3D-m1_glrlm_HighGrayLevelRunEmphasis | 0.936908227 |
| lbp-3D-m1_glrlm_LongRunEmphasis | 0.587356227 |
| lbp-3D-m1_glrlm_LongRunHighGrayLevelEmphasis | 0.819945519 |
| lbp-3D-m1_glrlm_LongRunLowGrayLevelEmphasis | 0.607013736 |
| lbp-3D-m1_glrlm_LowGrayLevelRunEmphasis | 0.963919619 |
| lbp-3D-m1_glrlm_RunEntropy | 0.707134046 |
| lbp-3D-m1_glrlm_RunLengthNonUniformity | 0.937929333 |
| lbp-3D-m1_glrlm_RunLengthNonUniformityNormalized | 0.397893456 |
| lbp-3D-m1_glrlm_RunPercentage | 0.429115959 |
| lbp-3D-m1_glrlm_RunVariance | 0.793627248 |
| lbp-3D-m1_glrlm_ShortRunEmphasis | 0.348781381 |
| lbp-3D-m1_glrlm_ShortRunHighGrayLevelEmphasis | 0.914216173 |
| lbp-3D-m1_glrlm_ShortRunLowGrayLevelEmphasis | 0.521747662 |
| lbp-3D-m1_ngtdm_Busyness | 0.922751566 |
| lbp-3D-m1_ngtdm_Coarseness | 0.776747001 |
| lbp-3D-m1_ngtdm_Complexity | 0.783833132 |
| lbp-3D-m1_ngtdm_Contrast | 0.675519804 |
| lbp-3D-m1_ngtdm_Strength | 0.708101348 |
| lbp-3D-m2_firstorder_ 10Percentile | 0.9680228 |
| lbp-3D-m2_firstorder_90Percentile | 0.667282851 |
| lbp-3D-m2_firstorder_Energy | 0.935263617 |
| lbp-3D-m2_firstorder_Entropy | 0.87001283 |
| lbp-3D-m2_firstorder_InterquartileRange | 0.961424082 |
| lbp-3D-m2_firstorder_Kurtosis | 0.438416795 |
| lbp-3D-m2_firstorder_Maximum | 0.951823089 |
| lbp-3D-m2_firstorder_MeanAbsoluteDeviation | 0.917544747 |
| lbp-3D-m2_firstorder_Mean | 0.973134532 |
| lbp-3D-m2_firstorder_Median | 0.966608293 |
| lbp-3D-m2_firstorder_Minimum | 0.994982394 |
| lbp-3D-m2_firstorder_Range | 0.968428492 |
| lbp-3D-m2_firstorder_RobustMeanAbsoluteDeviation | 0.95443867 |
| lbp-3D-m2_firstorder_RootMeanSquared | 0.959645012 |
| lbp-3D-m2_firstorder_Skewness | 0.921750277 |
| lbp-3D-m2_firstorder_TotalEnergy | 0.935263617 |
| lbp-3D-m2_firstorder_Uniformity | 0.880976776 |
| lbp-3D-m2_firstorder_Variance | 0.930865881 |
| lbp-3D-m2_glcm_Autocorrelation | 0.966557937 |
| lbp-3D-m2_glcm_ClusterProminence | 0.550913279 |
| lbp-3D-m2_glcm_ClusterShade | 0.766879818 |
| lbp-3D-m2_glcm_ClusterTendency | 0.742635711 |
| lbp-3D-m2_glcm_Contrast | 0.849147032 |
| lbp-3D-m2_glcm_Correlation | 0.763539089 |

| lbp-3D-m2_glcm_DifferenceAverage | 0.826362533 |
| --- | --- |
| lbp-3D-m2_glcm_DifferenceEntropy | 0.93971642 |
| lbp-3D-m2_glcm_DifferenceVariance | 0.9303606 |
| lbp-3D-m2_glcm_Id | 0.817963705 |
| lbp-3D-m2_glcm_Idm | 0.814122032 |
| lbp-3D-m2_glcm_Idmn | 0.841368994 |
| lbp-3D-m2_glcm_Idn | 0.820605749 |
| lbp-3D-m2_glcm_Imc1 | 0.916056615 |
| lbp-3D-m2_glcm_Imc2 | 0.877789858 |
| lbp-3D-m2_glcm_InverseVariance | 0.957357137 |
| lbp-3D-m2_glcm_JointAverage | 0.938851061 |
| lbp-3D-m2_glcm_JointEnergy | 0.91046142 |
| lbp-3D-m2_glcm_JointEntropy | 0.923797305 |
| lbp-3D-m2_glcm_MCC | 0.888994742 |
| lbp-3D-m2_glcm_MaximumProbability | 0.944532159 |
| lbp-3D-m2_glcm_SumAverage | 0.938851061 |
| lbp-3D-m2_glcm_SumEntropy | 0.909272634 |
| lbp-3D-m2_glcm_SumSquares | 0.867011152 |
| lbp-3D-m2_gldm_DependenceEntropy | 0.959638586 |
| lbp-3D-m2_gldm_DependenceNonUniformity | 0.93777874 |
| lbp-3D-m2_gldm_DependenceNonUniformityNormalized | 0.954495398 |
| lbp-3D-m2_gldm_DependenceVariance | 0.894818893 |
| lbp-3D-m2_gldm_GrayLevelNonUniformity | 0.932140174 |
| lbp-3D-m2_gldm_GrayLevelVariance | 0.922671767 |
| lbp-3D-m2_gldm_HighGrayLevelEmphasis | 0.970563951 |
| lbp-3D-m2_gldm_LargeDependenceEmphasis | 0.903530945 |
| lbp-3D-m2_gldm_LargeDependenceHighGrayLevelEmphasis | 0.920485591 |
| lbp-3D-m2_gldm_LargeDependenceLowGrayLevelEmphasis | 0.973856169 |
| lbp-3D-m2_gldm_LowGrayLevelEmphasis | 0.977290217 |
| lbp-3D-m2_gldm_SmallDependenceEmphasis | 0.928604806 |
| lbp-3D-m2_gldm_SmallDependenceHighGrayLevelEmphasis | 0.814304856 |
| lbp-3D-m2_gldm_SmallDependenceLowGrayLevelEmphasis | 0.934087364 |
| lbp-3D-m2_glszm_GrayLevelNonUniformity | 0.954715378 |
| lbp-3D-m2_glszm_GrayLevelNonUniformityNormalized | 0.583898407 |
| lbp-3D-m2_glszm_GrayLevelVariance | 0.643718089 |
| lbp-3D-m2_glszm_HighGrayLevelZoneEmphasis | 0.769198225 |
| lbp-3D-m2_glszm_LargeAreaEmphasis | 0.927028864 |
| lbp-3D-m2_glszm_LargeAreaHighGrayLevelEmphasis | 0.921879284 |
| lbp-3D-m2_glszm_LargeAreaLowGrayLevelEmphasis | 0.926377233 |
| lbp-3D-m2_glszm_LowGrayLevelZoneEmphasis | 0.904411138 |
| lbp-3D-m2_glszm_SizeZoneNonUniformity | 0.864391816 |
| lbp-3D-m2_glszm_SizeZoneNonUniformityNormalized | 0.59419682 |
| lbp-3D-m2_glszm_SmallAreaEmphasis | 0.699331788 |
| lbp-3D-m2_glszm_SmallAreaHighGrayLevelEmphasis | 0.412888345 |
| lbp-3D-m2_glszm_SmallAreaLowGrayLevelEmphasis | 0.885476082 |
| lbp-3D-m2_glszm_ZoneEntropy | 0.868915878 |
| lbp-3D-m2_glszm_ZonePercentage | 0.901715807 |
| lbp-3D-m2_glszm_ZoneVariance | 0.926511386 |
| lbp-3D-m2_glrlm_GrayLevelNonUniformity | 0.933678372 |
| lbp-3D-m2_glrlm_GrayLevelNonUniformityNormalized | 0.964014722 |
| lbp-3D-m2_glrlm_GrayLevelVariance | 0.937481709 |
| lbp-3D-m2_glrlm_HighGrayLevelRunEmphasis | 0.965554632 |
| lbp-3D-m2_glrlm_LongRunEmphasis | 0.566271547 |
| lbp-3D-m2_glrlm_LongRunHighGrayLevelEmphasis | 0.927626259 |
| lbp-3D-m2_glrlm_LongRunLowGrayLevelEmphasis | 0.575089509 |
| lbp-3D-m2_glrlm_LowGrayLevelRunEmphasis | 0.973073696 |

| lbp-3D-m2_glrlm_RunEntropy | 0.587929026 |
| --- | --- |
| lbp-3D-m2_glrlm_RunLengthNonUniformity | 0.936339125 |
| lbp-3D-m2_glrlm_RunLengthNonUniformityNormalized | 0.532657198 |
| lbp-3D-m2_glrlm_RunPercentage | 0.521814286 |
| lbp-3D-m2_glrlm_RunVariance | 0.780862076 |
| lbp-3D-m2_glrlm_ShortRunEmphasis | 0.362447966 |
| lbp-3D-m2_glrlm_ShortRunHighGrayLevelEmphasis | 0.930301599 |
| lbp-3D-m2_glrlm_ShortRunLowGrayLevelEmphasis | 0.626876546 |
| lbp-3D-m2_ngtdm_Busyness | 0.937820126 |
| lbp-3D-m2_ngtdm_Coarseness | 0.79881671 |
| lbp-3D-m2_ngtdm_Complexity | 0.921041414 |
| lbp-3D-m2_ngtdm_Contrast | 0.960745198 |
| lbp-3D-m2_ngtdm_Strength | 0.685934789 |
| lbp-3D-k_firstorder_ 10Percentile | 0.965378118 |
| lbp-3D-k_firstorder_90Percentile | 0.942444747 |
| lbp-3D-k_firstorder_Energy | 0.487890881 |
| lbp-3D-k_firstorder_Entropy | 0.929992356 |
| lbp-3D-k_firstorder_InterquartileRange | 0.956345025 |
| lbp-3D-k_firstorder_Kurtosis | 0.846851682 |
| lbp-3D-k_firstorder_Maximum | 0.708808522 |
| lbp-3D-k_firstorder_MeanAbsoluteDeviation | 0.83927499 |
| lbp-3D-k_firstorder_Mean | 0.960771672 |
| lbp-3D-k_firstorder_Median | 0.972996373 |
| lbp-3D-k_firstorder_Minimum | 0.963241735 |
| lbp-3D-k_firstorder_Range | 0.70564523 |
| lbp-3D-k_firstorder_RobustMeanAbsoluteDeviation | 0.943959996 |
| lbp-3D-k_firstorder_RootMeanSquared | 0.806131699 |
| lbp-3D-k_firstorder_Skewness | 0.860859051 |
| lbp-3D-k_firstorder_TotalEnergy | 0.487890881 |
| lbp-3D-k_firstorder_Uniformity | 0.935921953 |
| lbp-3D-k_firstorder_Variance | 0.451946617 |
| lbp-3D-k_glcm_Autocorrelation | 0.948050449 |
| lbp-3D-k_glcm_ClusterProminence | 0.191766654 |
| lbp-3D-k_glcm_ClusterShade | 0.533445664 |
| lbp-3D-k_glcm_ClusterTendency | 0.909663687 |
| lbp-3D-k_glcm_Contrast | 0.962553908 |
| lbp-3D-k_glcm_Correlation | 0.711349648 |
| lbp-3D-k_glcm_DifferenceAverage | 0.962266947 |
| lbp-3D-k_glcm_DifferenceEntropy | 0.958040555 |
| lbp-3D-k_glcm_DifferenceVariance | 0.960170001 |
| lbp-3D-k_glcm_Id | 0.962166017 |
| lbp-3D-k_glcm_Idm | 0.962206811 |
| lbp-3D-k_glcm_Idmn | 0.901717349 |
| lbp-3D-k_glcm_Idn | 0.946578181 |
| lbp-3D-k_glcm_Imc1 | 0.795653629 |
| lbp-3D-k_glcm_Imc2 | 0.861456593 |
| lbp-3D-k_glcm_InverseVariance | 0.961996163 |
| lbp-3D-k_glcm_JointAverage | 0.956562298 |
| lbp-3D-k_glcm_JointEnergy | 0.959279995 |
| lbp-3D-k_glcm_JointEntropy | 0.957763294 |
| lbp-3D-k_glcm_MCC | 0.683225094 |
| lbp-3D-k_glcm_MaximumProbability | 0.961030585 |
| lbp-3D-k_glcm_SumAverage | 0.956562298 |
| lbp-3D-k_glcm_SumEntropy | 0.955507844 |
| lbp-3D-k_glcm_SumSquares | 0.943129482 |
| lbp-3D-k_gldm_DependenceEntropy | 0.894679 |

| lbp-3D-k_gldm_DependenceNonUniformity | 0.936592166 |
| --- | --- |
| lbp-3D-k_gldm_DependenceNonUniformityNormalized | 0.862115813 |
| lbp-3D-k_gldm_DependenceVariance | 0.889380341 |
| lbp-3D-k_gldm_GrayLevelNonUniformity | 0.947879545 |
| lbp-3D-k_gldm_GrayLevelVariance | 0.85559917 |
| lbp-3D-k_gldm_HighGrayLevelEmphasis | 0.900678962 |
| lbp-3D-k_gldm_LargeDependenceEmphasis | 0.94776284 |
| lbp-3D-k_gldm_LargeDependenceHighGrayLevelEmphasis | 0.95176963 |
| lbp-3D-k_gldm_LargeDependenceLowGrayLevelEmphasis | 0.946788166 |
| lbp-3D-k_gldm_LowGrayLevelEmphasis | 0.932895527 |
| lbp-3D-k_gldm_SmallDependenceEmphasis | 0.870632411 |
| lbp-3D-k_gldm_SmallDependenceHighGrayLevelEmphasis | 0.768390186 |
| lbp-3D-k_gldm_SmallDependenceLowGrayLevelEmphasis | 0.955700134 |
| lbp-3D-k_glszm_GrayLevelNonUniformity | 0.934034213 |
| lbp-3D-k_glszm_GrayLevelNonUniformityNormalized | 0.749859509 |
| lbp-3D-k_glszm_GrayLevelVariance | 0.500441282 |
| lbp-3D-k_glszm_HighGrayLevelZoneEmphasis | 0.800397655 |
| lbp-3D-k_glszm_LargeAreaEmphasis | 0.93140565 |
| lbp-3D-k_glszm_LargeAreaHighGrayLevelEmphasis | 0.931140757 |
| lbp-3D-k_glszm_LargeAreaLowGrayLevelEmphasis | 0.931456636 |
| lbp-3D-k_glszm_LowGrayLevelZoneEmphasis | 0.850883408 |
| lbp-3D-k_glszm_SizeZoneNonUniformity | 0.849706775 |
| lbp-3D-k_glszm_SizeZoneNonUniformityNormalized | 0.724218464 |
| lbp-3D-k_glszm_SmallAreaEmphasis | 0.547766437 |
| lbp-3D-k_glszm_SmallAreaHighGrayLevelEmphasis | 0.624002463 |
| lbp-3D-k_glszm_SmallAreaLowGrayLevelEmphasis | 0.460077015 |
| lbp-3D-k_glszm_ZoneEntropy | 0.830024673 |
| lbp-3D-k_glszm_ZonePercentage | 0.836842393 |
| lbp-3D-k_glszm_ZoneVariance | 0.936268308 |
| lbp-3D-k_glrlm_GrayLevelNonUniformity | 0.945654811 |
| lbp-3D-k_glrlm_GrayLevelNonUniformityNormalized | 0.932595132 |
| lbp-3D-k_glrlm_GrayLevelVariance | 0.841016044 |
| lbp-3D-k_glrlm_HighGrayLevelRunEmphasis | 0.887526867 |
| lbp-3D-k_glrlm_LongRunEmphasis | 0.875124522 |
| lbp-3D-k_glrlm_LongRunHighGrayLevelEmphasis | 0.870662497 |
| lbp-3D-k_glrlm_LongRunLowGrayLevelEmphasis | 0.876220062 |
| lbp-3D-k_glrlm_LowGrayLevelRunEmphasis | 0.925901885 |
| lbp-3D-k_glrlm_RunEntropy | 0.780900483 |
| lbp-3D-k_glrlm_RunLengthNonUniformity | 0.840804931 |
| lbp-3D-k_glrlm_RunLengthNonUniformityNormalized | 0.400668541 |
| lbp-3D-k_glrlm_RunPercentage | 0.286456628 |
| lbp-3D-k_glrlm_RunVariance | 0.876305206 |
| lbp-3D-k_glrlm_ShortRunEmphasis | 0.497507037 |
| lbp-3D-k_glrlm_ShortRunHighGrayLevelEmphasis | 0.877207815 |
| lbp-3D-k_glrlm_ShortRunLowGrayLevelEmphasis | 0.098170341 |
| lbp-3D-k_ngtdm_Busyness | 0.972422185 |
| lbp-3D-k_ngtdm_Coarseness | 0.794952977 |
| lbp-3D-k_ngtdm_Complexity | 0.673110358 |
| lbp-3D-k_ngtdm_Contrast | 0.884703239 |
| lbp-3D-k_ngtdm_Strength | 0.543251634 |
| logarithm_firstorder_ 10Percentile | 0.597493163 |
| logarithm_firstorder_90Percentile | 0.99692024 |
| logarithm_firstorder_Energy | 0.984626809 |
| logarithm_firstorder_Entropy | 0.917583846 |
| logarithm_firstorder_InterquartileRange | 0.881297754 |
| logarithm_firstorder_Kurtosis | 0.257048589 |

| logarithm_firstorder_Maximum | 0.989118599 |
| --- | --- |
| logarithm_firstorder_MeanAbsoluteDeviation | 0.56461266 |
| logarithm_firstorder_Mean | 0.972923017 |
| logarithm_firstorder_Median | 0.991564804 |
| logarithm_firstorder_Minimum | 0.445788872 |
| logarithm_firstorder_Range | 0.661163647 |
| logarithm_firstorder_RobustMeanAbsoluteDeviation | 0.329745291 |
| logarithm_firstorder_RootMeanSquared | 0.988355457 |
| logarithm_firstorder_Skewness | 0.222183226 |
| logarithm_firstorder_TotalEnergy | 0.984626809 |
| logarithm_firstorder_Uniformity | 0.94073213 |
| logarithm_firstorder_Variance | 0.340983059 |
| logarithm_glcm_Autocorrelation | 0.600918691 |
| logarithm_glcm_ClusterProminence | 0.249458803 |
| logarithm_glcm_ClusterShade | 0.551024846 |
| logarithm_glcm_ClusterTendency | 0.861921671 |
| logarithm_glcm_Contrast | 0.824756274 |
| logarithm_glcm_Correlation | 0.558427997 |
| logarithm_glcm_DifferenceAverage | 0.912785543 |
| logarithm_glcm_DifferenceEntropy | 0.918556229 |
| logarithm_glcm_DifferenceVariance | 0.775500367 |
| logarithm_glcm_Id | 0.928058043 |
| logarithm_glcm_Idm | 0.923040423 |
| logarithm_glcm_Idmn | 0.819518698 |
| logarithm_glcm_Idn | 0.913935647 |
| logarithm_glcm_Imc1 | 0.604899043 |
| logarithm_glcm_Imc2 | 0.813940926 |
| logarithm_glcm_InverseVariance | 0.931836494 |
| logarithm_glcm_JointAverage | 0.567110122 |
| logarithm_glcm_JointEnergy | 0.941360969 |
| logarithm_glcm_JointEntropy | 0.931323076 |
| logarithm_glcm_MCC | 0.283315883 |
| logarithm_glcm_MaximumProbability | 0.949704217 |
| logarithm_glcm_SumAverage | 0.567110122 |
| logarithm_glcm_SumEntropy | 0.933476484 |
| logarithm_glcm_SumSquares | 0.848972832 |
| logarithm_gldm_DependenceEntropy | 0.894120153 |
| logarithm_gldm_DependenceNonUniformity | 0.909709495 |
| logarithm_gldm_DependenceNonUniformityNormalized | 0.787624458 |
| logarithm_gldm_DependenceVariance | 0.910009174 |
| logarithm_gldm_GrayLevelNonUniformity | 0.95065544 |
| logarithm_gldm_GrayLevelVariance | 0.843641646 |
| logarithm_gldm_HighGrayLevelEmphasis | 0.602639271 |
| logarithm_gldm_LargeDependenceEmphasis | 0.957651802 |
| logarithm_gldm_LargeDependenceHighGrayLevelEmphasis | 0.766151801 |
| logarithm_gldm_LargeDependenceLowGrayLevelEmphasis | 0.454618734 |
| logarithm_gldm_LowGrayLevelEmphasis | 0.413659071 |
| logarithm_gldm_SmallDependenceEmphasis | 0.923539491 |
| logarithm_gldm_SmallDependenceHighGrayLevelEmphasis | 0.610598224 |
| logarithm_gldm_SmallDependenceLowGrayLevelEmphasis | 0.754640626 |
| logarithm_glszm_GrayLevelNonUniformity | 0.792932736 |
| logarithm_glszm_GrayLevelNonUniformityNormalized | 0.325311901 |
| logarithm_glszm_GrayLevelVariance | 0.484907742 |
| logarithm_glszm_HighGrayLevelZoneEmphasis | 0.572998684 |
| logarithm_glszm_LargeAreaEmphasis | 0.592230245 |
| logarithm_glszm_LargeAreaHighGrayLevelEmphasis | 0.681371079 |

| logarithm_glszm_LargeAreaLowGrayLevelEmphasis | 0.170768422 |
| --- | --- |
| logarithm_glszm_LowGrayLevelZoneEmphasis | 0.545599934 |
| logarithm_glszm_SizeZoneNonUniformity | 0.625761471 |
| logarithm_glszm_SizeZoneNonUniformityNormalized | 0.615127202 |
| logarithm_glszm_SmallAreaEmphasis | 0.360091398 |
| logarithm_glszm_SmallAreaHighGrayLevelEmphasis | 0.589060429 |
| logarithm_glszm_SmallAreaLowGrayLevelEmphasis | 0.190211615 |
| logarithm_glszm_ZoneEntropy | 0.740397391 |
| logarithm_glszm_ZonePercentage | 0.93501164 |
| logarithm_glszm_ZoneVariance | 0.792267253 |
| logarithm_glrlm_GrayLevelNonUniformity | 0.958583714 |
| logarithm_glrlm_GrayLevelNonUniformityNormalized | 0.883763573 |
| logarithm_glrlm_GrayLevelVariance | 0.658006728 |
| logarithm_glrlm_HighGrayLevelRunEmphasis | 0.560046917 |
| logarithm_glrlm_LongRunEmphasis | 0.950950902 |
| logarithm_glrlm_LongRunHighGrayLevelEmphasis | 0.684103977 |
| logarithm_glrlm_LongRunLowGrayLevelEmphasis | 0.542616899 |
| logarithm_glrlm_LowGrayLevelRunEmphasis | 0.357297297 |
| logarithm_glrlm_RunEntropy | 0.782319329 |
| logarithm_glrlm_RunLengthNonUniformity | 0.940030001 |
| logarithm_glrlm_RunLengthNonUniformityNormalized | 0.610435942 |
| logarithm_glrlm_RunPercentage | 0.731283606 |
| logarithm_glrlm_RunVariance | 0.901666472 |
| logarithm_glrlm_ShortRunEmphasis | 0.76530778 |
| logarithm_glrlm_ShortRunHighGrayLevelEmphasis | 0.453697761 |
| logarithm_glrlm_ShortRunLowGrayLevelEmphasis | 0.346913449 |
| logarithm_ngtdm_Busyness | 0.055312598 |
| logarithm_ngtdm_Coarseness | 0.459834173 |
| logarithm_ngtdm_Complexity | 0.412935129 |
| logarithm_ngtdm_Contrast | 0.747915452 |
| logarithm_ngtdm_Strength | 0.429829623 |
| square_firstorder_ 10Percentile | 0.599209481 |
| square_firstorder_90Percentile | 0.991555253 |
| square_firstorder_Energy | 0.996847321 |
| square_firstorder_Entropy | 0.996348477 |
| square_firstorder_InterquartileRange | 0.931487691 |
| square_firstorder_Kurtosis | 0.681219219 |
| square_firstorder_Maximum | 0.905302198 |
| square_firstorder_MeanAbsoluteDeviation | 0.969979794 |
| square_firstorder_Mean | 0.949757329 |
| square_firstorder_Median | 0.95108376 |
| square_firstorder_Minimum | 0.349398104 |
| square_firstorder_Range | 0.894647801 |
| square_firstorder_RobustMeanAbsoluteDeviation | 0.95055508 |
| square_firstorder_RootMeanSquared | 0.97181245 |
| square_firstorder_Skewness | 0.830400279 |
| square_firstorder_TotalEnergy | 0.996847321 |
| square_firstorder_Uniformity | 0.996752987 |
| square_firstorder_Variance | 0.982596242 |
| square_glcm_Autocorrelation | 0.997090208 |
| square_glcm_ClusterProminence | 0.997578406 |
| square_glcm_ClusterShade | 0.994622646 |
| square_glcm_ClusterTendency | 0.997679287 |
| square_glcm_Contrast | 0.995402748 |
| square_glcm_Correlation | 0.727805395 |
| square_glcm_DifferenceAverage | 0.995404377 |

| square_glcm_DifferenceEntropy | 0.992057598 |
| --- | --- |
| square_glcm_DifferenceVariance | 0.993460613 |
| square_glcm_Id | 0.995404917 |
| square_glcm_Idm | 0.995404701 |
| square_glcm_Idmn | 0.995467805 |
| square_glcm_Idn | 0.995439196 |
| square_glcm_Imc1 | 0.940109722 |
| square_glcm_Imc2 | 0.987863087 |
| square_glcm_InverseVariance | 0.99540579 |
| square_glcm_JointAverage | 0.996814706 |
| square_glcm_JointEnergy | 0.997155873 |
| square_glcm_JointEntropy | 0.996176079 |
| square_glcm_MCC | 0.691857407 |
| square_glcm_MaximumProbability | 0.997446041 |
| square_glcm_SumAverage | 0.996814706 |
| square_glcm_SumEntropy | 0.994918555 |
| square_glcm_SumSquares | 0.997746643 |
| square_gldm_DependenceEntropy | 0.88756687 |
| square_gldm_DependenceNonUniformity | 0.915708216 |
| square_gldm_DependenceNonUniformityNormalized | 0.767128264 |
| square_gldm_DependenceVariance | 0.846940791 |
| square_gldm_GrayLevelNonUniformity | 0.936140656 |
| square_gldm_GrayLevelVariance | 0.996753222 |
| square_gldm_HighGrayLevelEmphasis | 0.99212506 |
| square_gldm_LargeDependenceEmphasis | 0.97396476 |
| square_gldm_LargeDependenceHighGrayLevelEmphasis | 0.969186313 |
| square_gldm_LargeDependenceLowGrayLevelEmphasis | 0.975550455 |
| square_gldm_LowGrayLevelEmphasis | 0.992124841 |
| square_gldm_SmallDependenceEmphasis | 0.943461841 |
| square_gldm_SmallDependenceHighGrayLevelEmphasis | 0.918516018 |
| square_gldm_SmallDependenceLowGrayLevelEmphasis | 0.96130251 |
| square_glszm_GrayLevelNonUniformity | 0.98228976 |
| square_glszm_GrayLevelNonUniformityNormalized | 0.749275063 |
| square_glszm_GrayLevelVariance | 0.75218653 |
| square_glszm_HighGrayLevelZoneEmphasis | 0.853175381 |
| square_glszm_LargeAreaEmphasis | 0.827069413 |
| square_glszm_LargeAreaHighGrayLevelEmphasis | 0.826983143 |
| square_glszm_LargeAreaLowGrayLevelEmphasis | 0.827091212 |
| square_glszm_LowGrayLevelZoneEmphasis | 0.849213709 |
| square_glszm_SizeZoneNonUniformity | 0.922906605 |
| square_glszm_SizeZoneNonUniformityNormalized | 0.814490374 |
| square_glszm_SmallAreaEmphasis | 0.688895324 |
| square_glszm_SmallAreaHighGrayLevelEmphasis | 0.69618143 |
| square_glszm_SmallAreaLowGrayLevelEmphasis | 0.622779273 |
| square_glszm_ZoneEntropy | 0.889600794 |
| square_glszm_ZonePercentage | 0.934000524 |
| square_glszm_ZoneVariance | 0.488845181 |
| square_glrlm_GrayLevelNonUniformity | 0.920501342 |
| square_glrlm_GrayLevelNonUniformityNormalized | 0.987895938 |
| square_glrlm_GrayLevelVariance | 0.987911952 |
| square_glrlm_HighGrayLevelRunEmphasis | 0.988701928 |
| square_glrlm_LongRunEmphasis | 0.946355761 |
| square_glrlm_LongRunHighGrayLevelEmphasis | 0.944014236 |
| square_glrlm_LongRunLowGrayLevelEmphasis | 0.946960154 |
| square_glrlm_LowGrayLevelRunEmphasis | 0.988695731 |
| square_glrlm_RunEntropy | 0.754252491 |

| square_glrlm_RunLengthNonUniformity | 0.862218751 |
| --- | --- |
| square_glrlm_RunLengthNonUniformityNormalized | 0.643614339 |
| square_glrlm_RunPercentage | 0.767936123 |
| square_glrlm_RunVariance | 0.937298021 |
| square_glrlm_ShortRunEmphasis | 0.82238632 |
| square_glrlm_ShortRunHighGrayLevelEmphasis | 0.97212559 |
| square_glrlm_ShortRunLowGrayLevelEmphasis | 0.651033103 |
| square_ngtdm_Busyness | 0.597247057 |
| square_ngtdm_Coarseness | 0.756189276 |
| square_ngtdm_Complexity | 0.9952999 |
| square_ngtdm_Contrast | 0.991900998 |
| square_ngtdm_Strength | 0.553304828 |
| squareroot_firstorder_ 10Percentile | 0.496238369 |
| squareroot_firstorder_90Percentile | 0.997883016 |
| squareroot_firstorder_Energy | 0.982623327 |
| squareroot_firstorder_Entropy | 0.923465517 |
| squareroot_firstorder_InterquartileRange | 0.909826538 |
| squareroot_firstorder_Kurtosis | 0.221750501 |
| squareroot_firstorder_Maximum | 0.988793976 |
| squareroot_firstorder_MeanAbsoluteDeviation | 0.549155619 |
| squareroot_firstorder_Mean | 0.968109984 |
| squareroot_firstorder_Median | 0.99183488 |
| squareroot_firstorder_Minimum | 0.421519061 |
| squareroot_firstorder_Range | 0.611027024 |
| squareroot_firstorder_RobustMeanAbsoluteDeviation | 0.316939252 |
| squareroot_firstorder_RootMeanSquared | 0.989142064 |
| squareroot_firstorder_Skewness | 0.17726267 |
| squareroot_firstorder_TotalEnergy | 0.982623327 |
| squareroot_firstorder_Uniformity | 0.946407171 |
| squareroot_firstorder_Variance | 0.319776681 |
| squareroot_glcm_Autocorrelation | 0.601977839 |
| squareroot_glcm_ClusterProminence | 0.265930189 |
| squareroot_glcm_ClusterShade | 0.565143365 |
| squareroot_glcm_ClusterTendency | 0.883389021 |
| squareroot_glcm_Contrast | 0.841341212 |
| squareroot_glcm_Correlation | 0.572191504 |
| squareroot_glcm_DifferenceAverage | 0.923084485 |
| squareroot_glcm_DifferenceEntropy | 0.920098369 |
| squareroot_glcm_DifferenceVariance | 0.789801152 |
| squareroot_glcm_Id | 0.936747404 |
| squareroot_glcm_Idm | 0.932315773 |
| squareroot_glcm_Idmn | 0.828423118 |
| squareroot_glcm_Idn | 0.922187729 |
| squareroot_glcm_Imc1 | 0.624606765 |
| squareroot_glcm_Imc2 | 0.809503068 |
| squareroot_glcm_InverseVariance | 0.939114408 |
| squareroot_glcm_JointAverage | 0.561946617 |
| squareroot_glcm_JointEnergy | 0.947691233 |
| squareroot_glcm_JointEntropy | 0.937902524 |
| squareroot_glcm_MCC | 0.291933588 |
| squareroot_glcm_MaximumProbability | 0.958964827 |
| squareroot_glcm_SumAverage | 0.561946617 |
| squareroot_glcm_SumEntropy | 0.940408013 |
| squareroot_glcm_SumSquares | 0.868738812 |
| squareroot_gldm_DependenceEntropy | 0.898908214 |
| squareroot_gldm_DependenceNonUniformity | 0.910373829 |

| squareroot_gldm_DependenceNonUniformityNormalized | 0.775332763 |
| --- | --- |
| squareroot_gldm_DependenceVariance | 0.913480969 |
| squareroot_gldm_GrayLevelNonUniformity | 0.951735933 |
| squareroot_gldm_GrayLevelVariance | 0.858746112 |
| squareroot_gldm_HighGrayLevelEmphasis | 0.602209377 |
| squareroot_gldm_LargeDependenceEmphasis | 0.959460533 |
| squareroot_gldm_LargeDependenceHighGrayLevelEmphasis | 0.761804092 |
| squareroot_gldm_LargeDependenceLowGrayLevelEmphasis | 0.439001716 |
| squareroot_gldm_LowGrayLevelEmphasis | 0.397929126 |
| squareroot_gldm_SmallDependenceEmphasis | 0.933566527 |
| squareroot_gldm_SmallDependenceHighGrayLevelEmphasis | 0.601695638 |
| squareroot_gldm_SmallDependenceLowGrayLevelEmphasis | 0.780977746 |
| squareroot_glszm_GrayLevelNonUniformity | 0.68198814 |
| squareroot_glszm_GrayLevelNonUniformityNormalized | 0.340077268 |
| squareroot_glszm_GrayLevelVariance | 0.462919484 |
| squareroot_glszm_HighGrayLevelZoneEmphasis | 0.566160954 |
| squareroot_glszm_LargeAreaEmphasis | 0.588859237 |
| squareroot_glszm_LargeAreaHighGrayLevelEmphasis | 0.671598599 |
| squareroot_glszm_LargeAreaLowGrayLevelEmphasis | 0.169777431 |
| squareroot_glszm_LowGrayLevelZoneEmphasis | 0.537548006 |
| squareroot_glszm_SizeZoneNonUniformity | 0.726024741 |
| squareroot_glszm_SizeZoneNonUniformityNormalized | 0.588792805 |
| squareroot_glszm_SmallAreaEmphasis | 0.443765873 |
| squareroot_glszm_SmallAreaHighGrayLevelEmphasis | 0.592641051 |
| squareroot_glszm_SmallAreaLowGrayLevelEmphasis | 0.151284397 |
| squareroot_glszm_ZoneEntropy | 0.725925607 |
| squareroot_glszm_ZonePercentage | 0.909214763 |
| squareroot_glszm_ZoneVariance | 0.790388848 |
| squareroot_glrlm_GrayLevelNonUniformity | 0.957808398 |
| squareroot_glrlm_GrayLevelNonUniformityNormalized | 0.888108644 |
| squareroot_glrlm_GrayLevelVariance | 0.679681939 |
| squareroot_glrlm_HighGrayLevelRunEmphasis | 0.54988715 |
| squareroot_glrlm_LongRunEmphasis | 0.952743381 |
| squareroot_glrlm_LongRunHighGrayLevelEmphasis | 0.674704602 |
| squareroot_glrlm_LongRunLowGrayLevelEmphasis | 0.536645654 |
| squareroot_glrlm_LowGrayLevelRunEmphasis | 0.346039296 |
| squareroot_glrlm_RunEntropy | 0.778566571 |
| squareroot_glrlm_RunLengthNonUniformity | 0.936260548 |
| squareroot_glrlm_RunLengthNonUniformityNormalized | 0.618291079 |
| squareroot_glrlm_RunPercentage | 0.741617977 |
| squareroot_glrlm_RunVariance | 0.904059559 |
| squareroot_glrlm_ShortRunEmphasis | 0.769859671 |
| squareroot_glrlm_ShortRunHighGrayLevelEmphasis | 0.468838893 |
| squareroot_glrlm_ShortRunLowGrayLevelEmphasis | 0.344722373 |
| squareroot_ngtdm_Busyness | 0.295582039 |
| squareroot_ngtdm_Coarseness | 0.459834154 |
| squareroot_ngtdm_Complexity | 0.416734112 |
| squareroot_ngtdm_Contrast | 0.756775211 |
| squareroot_ngtdm_Strength | 0.424043464 |
| wavelet-LLH_firstorder_ 10Percentile | 0.979594255 |
| wavelet-LLH_firstorder_90Percentile | 0.993884392 |
| wavelet-LLH_firstorder_Energy | 0.978639571 |
| wavelet-LLH_firstorder_Entropy | 0.958613246 |
| wavelet-LLH_firstorder_InterquartileRange | 0.991949628 |
| wavelet-LLH_firstorder_Kurtosis | 0.956062719 |
| wavelet-LLH_firstorder_Maximum | 0.963344403 |

| wavelet-LLH_firstorder_MeanAbsoluteDeviation | 0.992879835 |
| --- | --- |
| wavelet-LLH_firstorder_Mean | 0.926993104 |
| wavelet-LLH_firstorder_Median | 0.926585027 |
| wavelet-LLH_firstorder_Minimum | 0.94374609 |
| wavelet-LLH_firstorder_Range | 0.965627231 |
| wavelet-LLH_firstorder_RobustMeanAbsoluteDeviation | 0.991834204 |
| wavelet-LLH_firstorder_RootMeanSquared | 0.992421459 |
| wavelet-LLH_firstorder_Skewness | 0.870813427 |
| wavelet-LLH_firstorder_TotalEnergy | 0.978639571 |
| wavelet-LLH_firstorder_Uniformity | 0.928059915 |
| wavelet-LLH_firstorder_Variance | 0.993934105 |
| wavelet-LLH_glcm_Autocorrelation | 0.908988604 |
| wavelet-LLH_glcm_ClusterProminence | 0.991366429 |
| wavelet-LLH_glcm_ClusterShade | 0.945793297 |
| wavelet-LLH_glcm_ClusterTendency | 0.971916687 |
| wavelet-LLH_glcm_Contrast | 0.975619808 |
| wavelet-LLH_glcm_Correlation | 0.789141217 |
| wavelet-LLH_glcm_DifferenceAverage | 0.939563645 |
| wavelet-LLH_glcm_DifferenceEntropy | 0.938889694 |
| wavelet-LLH_glcm_DifferenceVariance | 0.894702694 |
| wavelet-LLH_glcm_Id | 0.880086407 |
| wavelet-LLH_glcm_Idm | 0.904587167 |
| wavelet-LLH_glcm_Idmn | 0.889377088 |
| wavelet-LLH_glcm_Idn | 0.86131541 |
| wavelet-LLH_glcm_Imc1 | 0.895375998 |
| wavelet-LLH_glcm_Imc2 | 0.881617213 |
| wavelet-LLH_glcm_InverseVariance | 0.907204146 |
| wavelet-LLH_glcm_JointAverage | 0.930193676 |
| wavelet-LLH_glcm_JointEnergy | 0.837960753 |
| wavelet-LLH_glcm_JointEntropy | 0.931688697 |
| wavelet-LLH_glcm_MCC | 0.855300369 |
| wavelet-LLH_glcm_MaximumProbability | 0.82971789 |
| wavelet-LLH_glcm_SumAverage | 0.930193676 |
| wavelet-LLH_glcm_SumEntropy | 0.903503004 |
| wavelet-LLH_glcm_SumSquares | 0.993014902 |
| wavelet-LLH_gldm_DependenceEntropy | 0.933030112 |
| wavelet-LLH_gldm_DependenceNonUniformity | 0.923982023 |
| wavelet-LLH_gldm_DependenceNonUniformityNormalized | 0.899908119 |
| wavelet-LLH_gldm_DependenceVariance | 0.917270245 |
| wavelet-LLH_gldm_GrayLevelNonUniformity | 0.932753791 |
| wavelet-LLH_gldm_GrayLevelVariance | 0.99373321 |
| wavelet-LLH_gldm_HighGrayLevelEmphasis | 0.919877587 |
| wavelet-LLH_gldm_LargeDependenceEmphasis | 0.941864904 |
| wavelet-LLH_gldm_LargeDependenceHighGrayLevelEmphasis | 0.941576702 |
| wavelet-LLH_gldm_LargeDependenceLowGrayLevelEmphasis | 0.918400158 |
| wavelet-LLH_gldm_LowGrayLevelEmphasis | 0.946013417 |
| wavelet-LLH_gldm_SmallDependenceEmphasis | 0.907608762 |
| wavelet-LLH_gldm_SmallDependenceHighGrayLevelEmphasis | 0.768409869 |
| wavelet-LLH_gldm_SmallDependenceLowGrayLevelEmphasis | 0.958561082 |
| wavelet-LLH_glszm_GrayLevelNonUniformity | 0.824411576 |
| wavelet-LLH_glszm_GrayLevelNonUniformityNormalized | 0.681002714 |
| wavelet-LLH_glszm_GrayLevelVariance | 0.887075961 |
| wavelet-LLH_glszm_HighGrayLevelZoneEmphasis | 0.874972484 |
| wavelet-LLH_glszm_LargeAreaEmphasis | 0.879348435 |
| wavelet-LLH_glszm_LargeAreaHighGrayLevelEmphasis | 0.936326121 |
| wavelet-LLH_glszm_LargeAreaLowGrayLevelEmphasis | 0.824852805 |

| wavelet-LLH_glszm_LowGrayLevelZoneEmphasis | 0.835505959 |
| --- | --- |
| wavelet-LLH_glszm_SizeZoneNonUniformity | 0.590358695 |
| wavelet-LLH_glszm_SizeZoneNonUniformityNormalized | 0.640100382 |
| wavelet-LLH_glszm_SmallAreaEmphasis | 0.484330555 |
| wavelet-LLH_glszm_SmallAreaHighGrayLevelEmphasis | 0.494293992 |
| wavelet-LLH_glszm_SmallAreaLowGrayLevelEmphasis | 0.770023972 |
| wavelet-LLH_glszm_ZoneEntropy | 0.894359469 |
| wavelet-LLH_glszm_ZonePercentage | 0.937554835 |
| wavelet-LLH_glszm_ZoneVariance | 0.87346371 |
| wavelet-LLH_glrlm_GrayLevelNonUniformity | 0.926881955 |
| wavelet-LLH_glrlm_GrayLevelNonUniformityNormalized | 0.9288099 |
| wavelet-LLH_glrlm_GrayLevelVariance | 0.990219143 |
| wavelet-LLH_glrlm_HighGrayLevelRunEmphasis | 0.921642842 |
| wavelet-LLH_glrlm_LongRunEmphasis | 0.920992249 |
| wavelet-LLH_glrlm_LongRunHighGrayLevelEmphasis | 0.968362322 |
| wavelet-LLH_glrlm_LongRunLowGrayLevelEmphasis | 0.91040683 |
| wavelet-LLH_glrlm_LowGrayLevelRunEmphasis | 0.944589166 |
| wavelet-LLH_glrlm_RunEntropy | 0.760945564 |
| wavelet-LLH_glrlm_RunLengthNonUniformity | 0.910402374 |
| wavelet-LLH_glrlm_RunLengthNonUniformityNormalized | 0.738807641 |
| wavelet-LLH_glrlm_RunPercentage | 0.604437813 |
| wavelet-LLH_glrlm_RunVariance | 0.936153933 |
| wavelet-LLH_glrlm_ShortRunEmphasis | 0.603855078 |
| wavelet-LLH_glrlm_ShortRunHighGrayLevelEmphasis | 0.897874514 |
| wavelet-LLH_glrlm_ShortRunLowGrayLevelEmphasis | 0.928813708 |
| wavelet-LLH_ngtdm_Busyness | 0.800093133 |
| wavelet-LLH_ngtdm_Coarseness | 0.923095226 |
| wavelet-LLH_ngtdm_Complexity | 0.904449302 |
| wavelet-LLH_ngtdm_Contrast | 0.945826873 |
| wavelet-LLH_ngtdm_Strength | 0.93977082 |
| wavelet-LHL_firstorder_ 10Percentile | 0.967447579 |
| wavelet-LHL_firstorder_90Percentile | 0.961713829 |
| wavelet-LHL_firstorder_Energy | 0.926274028 |
| wavelet-LHL_firstorder_Entropy | 0.930848021 |
| wavelet-LHL_firstorder_InterquartileRange | 0.989778021 |
| wavelet-LHL_firstorder_Kurtosis | 0.721299771 |
| wavelet-LHL_firstorder_Maximum | 0.812796329 |
| wavelet-LHL_firstorder_MeanAbsoluteDeviation | 0.970816043 |
| wavelet-LHL_firstorder_Mean | 0.878886399 |
| wavelet-LHL_firstorder_Median | 0.945983933 |
| wavelet-LHL_firstorder_Minimum | 0.866997631 |
| wavelet-LHL_firstorder_Range | 0.891787249 |
| wavelet-LHL_firstorder_RobustMeanAbsoluteDeviation | 0.989576204 |
| wavelet-LHL_firstorder_RootMeanSquared | 0.94410048 |
| wavelet-LHL_firstorder_Skewness | 0.683355541 |
| wavelet-LHL_firstorder_TotalEnergy | 0.926274028 |
| wavelet-LHL_firstorder_Uniformity | 0.933562694 |
| wavelet-LHL_firstorder_Variance | 0.923761414 |
| wavelet-LHL_glcm_Autocorrelation | 0.235967524 |
| wavelet-LHL_glcm_ClusterProminence | 0.896990805 |
| wavelet-LHL_glcm_ClusterShade | 0.961259583 |
| wavelet-LHL_glcm_ClusterTendency | 0.909790167 |
| wavelet-LHL_glcm_Contrast | 0.965720601 |
| wavelet-LHL_glcm_Correlation | 0.939411706 |
| wavelet-LHL_glcm_DifferenceAverage | 0.965714123 |
| wavelet-LHL_glcm_DifferenceEntropy | 0.920829338 |

| wavelet-LHL_glcm_DifferenceVariance | 0.922628461 |
| --- | --- |
| wavelet-LHL_glcm_Id | 0.96571191 |
| wavelet-LHL_glcm_Idm | 0.965712798 |
| wavelet-LHL_glcm_Idmn | 0.712762742 |
| wavelet-LHL_glcm_Idn | 0.889625386 |
| wavelet-LHL_glcm_Imc1 | 0.856253375 |
| wavelet-LHL_glcm_Imc2 | 0.856365485 |
| wavelet-LHL_glcm_InverseVariance | 0.965708259 |
| wavelet-LHL_glcm_JointAverage | 0.361990344 |
| wavelet-LHL_glcm_JointEnergy | 0.938280149 |
| wavelet-LHL_glcm_JointEntropy | 0.939269651 |
| wavelet-LHL_glcm_MCC | 0.866028855 |
| wavelet-LHL_glcm_MaximumProbability | 0.965088624 |
| wavelet-LHL_glcm_SumAverage | 0.361990344 |
| wavelet-LHL_glcm_SumEntropy | 0.914448435 |
| wavelet-LHL_glcm_SumSquares | 0.937920366 |
| wavelet-LHL_gldm_DependenceEntropy | 0.933716527 |
| wavelet-LHL_gldm_DependenceNonUniformity | 0.938718978 |
| wavelet-LHL_gldm_DependenceNonUniformityNormalized | 0.902948649 |
| wavelet-LHL_gldm_DependenceVariance | 0.907679472 |
| wavelet-LHL_gldm_GrayLevelNonUniformity | 0.935337832 |
| wavelet-LHL_gldm_GrayLevelVariance | 0.933568726 |
| wavelet-LHL_gldm_HighGrayLevelEmphasis | 0.233300266 |
| wavelet-LHL_gldm_LargeDependenceEmphasis | 0.962676247 |
| wavelet-LHL_gldm_LargeDependenceHighGrayLevelEmphasis | 0.749154479 |
| wavelet-LHL_gldm_LargeDependenceLowGrayLevelEmphasis | 0.907686551 |
| wavelet-LHL_gldm_LowGrayLevelEmphasis | 0.539183904 |
| wavelet-LHL_gldm_SmallDependenceEmphasis | 0.950803451 |
| wavelet-LHL_gldm_SmallDependenceHighGrayLevelEmphasis | 0.885664546 |
| wavelet-LHL_gldm_SmallDependenceLowGrayLevelEmphasis | 0.955137463 |
| wavelet-LHL_glszm_GrayLevelNonUniformity | 0.554063279 |
| wavelet-LHL_glszm_GrayLevelNonUniformityNormalized | 0.387340778 |
| wavelet-LHL_glszm_GrayLevelVariance | 0.395409539 |
| wavelet-LHL_glszm_HighGrayLevelZoneEmphasis | 0.405624406 |
| wavelet-LHL_glszm_LargeAreaEmphasis | 0.869416504 |
| wavelet-LHL_glszm_LargeAreaHighGrayLevelEmphasis | 0.85827051 |
| wavelet-LHL_glszm_LargeAreaLowGrayLevelEmphasis | 0.866399927 |
| wavelet-LHL_glszm_LowGrayLevelZoneEmphasis | 0.42183452 |
| wavelet-LHL_glszm_SizeZoneNonUniformity | 0.378559775 |
| wavelet-LHL_glszm_SizeZoneNonUniformityNormalized | 0.339181465 |
| wavelet-LHL_glszm_SmallAreaEmphasis | 0.442348351 |
| wavelet-LHL_glszm_SmallAreaHighGrayLevelEmphasis | 0.479261347 |
| wavelet-LHL_glszm_SmallAreaLowGrayLevelEmphasis | 0.305164035 |
| wavelet-LHL_glszm_ZoneEntropy | 0.592356331 |
| wavelet-LHL_glszm_ZonePercentage | 0.957784473 |
| wavelet-LHL_glszm_ZoneVariance | 0.895097627 |
| wavelet-LHL_glrlm_GrayLevelNonUniformity | 0.934216296 |
| wavelet-LHL_glrlm_GrayLevelNonUniformityNormalized | 0.917661227 |
| wavelet-LHL_glrlm_GrayLevelVariance | 0.917332549 |
| wavelet-LHL_glrlm_HighGrayLevelRunEmphasis | 0.09960058 |
| wavelet-LHL_glrlm_LongRunEmphasis | 0.724182853 |
| wavelet-LHL_glrlm_LongRunHighGrayLevelEmphasis | 0.580869629 |
| wavelet-LHL_glrlm_LongRunLowGrayLevelEmphasis | 0.715354884 |
| wavelet-LHL_glrlm_LowGrayLevelRunEmphasis | 0.287106724 |
| wavelet-LHL_glrlm_RunEntropy | 0.679935449 |
| wavelet-LHL_glrlm_RunLengthNonUniformity | 0.921204678 |

| wavelet-LHL_glrlm_RunLengthNonUniformityNormalized | 0.338569389 |
| --- | --- |
| wavelet-LHL_glrlm_RunPercentage | 0.462060683 |
| wavelet-LHL_glrlm_RunVariance | 0.773377492 |
| wavelet-LHL_glrlm_ShortRunEmphasis | 0.32922428 |
| wavelet-LHL_glrlm_ShortRunHighGrayLevelEmphasis | 0.074066591 |
| wavelet-LHL_glrlm_ShortRunLowGrayLevelEmphasis | 0.170152112 |
| wavelet-LHL_ngtdm_Busyness | 0.222951254 |
| wavelet-LHL_ngtdm_Coarseness | 0.962948705 |
| wavelet-LHL_ngtdm_Complexity | 0.149853204 |
| wavelet-LHL_ngtdm_Contrast | 0.724632226 |
| wavelet-LHL_ngtdm_Strength | 0.96284787 |
| wavelet-LHH_firstorder_ 10Percentile | 0.979407101 |
| wavelet-LHH_firstorder_90Percentile | 0.972384441 |
| wavelet-LHH_firstorder_Energy | 0.932266133 |
| wavelet-LHH_firstorder_Entropy | 0.857691394 |
| wavelet-LHH_firstorder_InterquartileRange | 0.991676151 |
| wavelet-LHH_firstorder_Kurtosis | 0.696930654 |
| wavelet-LHH_firstorder_Maximum | 0.916918152 |
| wavelet-LHH_firstorder_MeanAbsoluteDeviation | 0.982589467 |
| wavelet-LHH_firstorder_Mean | 0.597947382 |
| wavelet-LHH_firstorder_Median | 0.809893529 |
| wavelet-LHH_firstorder_Minimum | 0.856873403 |
| wavelet-LHH_firstorder_Range | 0.905675443 |
| wavelet-LHH_firstorder_RobustMeanAbsoluteDeviation | 0.993470505 |
| wavelet-LHH_firstorder_RootMeanSquared | 0.970009986 |
| wavelet-LHH_firstorder_Skewness | 0.722423134 |
| wavelet-LHH_firstorder_TotalEnergy | 0.932266133 |
| wavelet-LHH_firstorder_Uniformity | 0.858923283 |
| wavelet-LHH_firstorder_Variance | 0.963319984 |
| wavelet-LHH_glcm_Autocorrelation | 0.899897263 |
| wavelet-LHH_glcm_ClusterProminence | 0.645535539 |
| wavelet-LHH_glcm_ClusterShade | 0.929102107 |
| wavelet-LHH_glcm_ClusterTendency | 0.558363762 |
| wavelet-LHH_glcm_Contrast | 0.715540963 |
| wavelet-LHH_glcm_Correlation | 0.657813298 |
| wavelet-LHH_glcm_DifferenceAverage | 0.715540963 |
| wavelet-LHH_glcm_DifferenceEntropy | 0.862820765 |
| wavelet-LHH_glcm_DifferenceVariance | 0.860425126 |
| wavelet-LHH_glcm_Id | 0.715540958 |
| wavelet-LHH_glcm_Idm | 0.715540958 |
| wavelet-LHH_glcm_Idmn | 0.715540963 |
| wavelet-LHH_glcm_Idn | 0.715540967 |
| wavelet-LHH_glcm_Imc1 | 0.856468915 |
| wavelet-LHH_glcm_Imc2 | 0.820529625 |
| wavelet-LHH_glcm_InverseVariance | 0.715540963 |
| wavelet-LHH_glcm_JointAverage | 0.905763089 |
| wavelet-LHH_glcm_JointEnergy | 0.877978192 |
| wavelet-LHH_glcm_JointEntropy | 0.870178764 |
| wavelet-LHH_glcm_MCC | 0.827246115 |
| wavelet-LHH_glcm_MaximumProbability | 0.953089064 |
| wavelet-LHH_glcm_SumAverage | 0.905763088 |
| wavelet-LHH_glcm_SumEntropy | 0.581462446 |
| wavelet-LHH_glcm_SumSquares | 0.880374843 |
| wavelet-LHH_gldm_DependenceEntropy | 0.876631256 |
| wavelet-LHH_gldm_DependenceNonUniformity | 0.937575192 |
| wavelet-LHH_gldm_DependenceNonUniformityNormalized | 0.883294859 |

| wavelet-LHH_gldm_DependenceVariance | 0.783558702 |
| --- | --- |
| wavelet-LHH_gldm_GrayLevelNonUniformity | 0.935393552 |
| wavelet-LHH_gldm_GrayLevelVariance | 0.858923278 |
| wavelet-LHH_gldm_HighGrayLevelEmphasis | 0.849807303 |
| wavelet-LHH_gldm_LargeDependenceEmphasis | 0.962527646 |
| wavelet-LHH_gldm_LargeDependenceHighGrayLevelEmphasis | 0.969503229 |
| wavelet-LHH_gldm_LargeDependenceLowGrayLevelEmphasis | 0.949222221 |
| wavelet-LHH_gldm_LowGrayLevelEmphasis | 0.849807299 |
| wavelet-LHH_gldm_SmallDependenceEmphasis | 0.94742222 |
| wavelet-LHH_gldm_SmallDependenceHighGrayLevelEmphasis | 0.935639411 |
| wavelet-LHH_gldm_SmallDependenceLowGrayLevelEmphasis | 0.946713551 |
| wavelet-LHH_glszm_GrayLevelNonUniformity | 0.530107371 |
| wavelet-LHH_glszm_GrayLevelNonUniformityNormalized | 0.240395531 |
| wavelet-LHH_glszm_GrayLevelVariance | 0.240395532 |
| wavelet-LHH_glszm_HighGrayLevelZoneEmphasis | 0.218153563 |
| wavelet-LHH_glszm_LargeAreaEmphasis | 0.752669645 |
| wavelet-LHH_glszm_LargeAreaHighGrayLevelEmphasis | 0.753395685 |
| wavelet-LHH_glszm_LargeAreaLowGrayLevelEmphasis | 0.751947623 |
| wavelet-LHH_glszm_LowGrayLevelZoneEmphasis | 0.218153563 |
| wavelet-LHH_glszm_SizeZoneNonUniformity | 0.255925829 |
| wavelet-LHH_glszm_SizeZoneNonUniformityNormalized | 0.20356697 |
| wavelet-LHH_glszm_SmallAreaEmphasis | 0.080084427 |
| wavelet-LHH_glszm_SmallAreaHighGrayLevelEmphasis | 0.219837404 |
| wavelet-LHH_glszm_SmallAreaLowGrayLevelEmphasis | -0.049436168 |
| wavelet-LHH_glszm_ZoneEntropy | 0.365438435 |
| wavelet-LHH_glszm_ZonePercentage | 0.928044063 |
| wavelet-LHH_glszm_ZoneVariance | 0.52122195 |
| wavelet-LHH_glrlm_GrayLevelNonUniformity | 0.935919519 |
| wavelet-LHH_glrlm_GrayLevelNonUniformityNormalized | 0.488633294 |
| wavelet-LHH_glrlm_GrayLevelVariance | 0.488633132 |
| wavelet-LHH_glrlm_HighGrayLevelRunEmphasis | 0.543828262 |
| wavelet-LHH_glrlm_LongRunEmphasis | 0.615970219 |
| wavelet-LHH_glrlm_LongRunHighGrayLevelEmphasis | 0.656734426 |
| wavelet-LHH_glrlm_LongRunLowGrayLevelEmphasis | 0.60836172 |
| wavelet-LHH_glrlm_LowGrayLevelRunEmphasis | 0.543828256 |
| wavelet-LHH_glrlm_RunEntropy | 0.499636966 |
| wavelet-LHH_glrlm_RunLengthNonUniformity | 0.932770954 |
| wavelet-LHH_glrlm_RunLengthNonUniformityNormalized | 0.312890788 |
| wavelet-LHH_glrlm_RunPercentage | 0.40031047 |
| wavelet-LHH_glrlm_RunVariance | 0.696777559 |
| wavelet-LHH_glrlm_ShortRunEmphasis | 0.317565492 |
| wavelet-LHH_glrlm_ShortRunHighGrayLevelEmphasis | 0.505474208 |
| wavelet-LHH_glrlm_ShortRunLowGrayLevelEmphasis | 0.186816051 |
| wavelet-LHH_ngtdm_Busyness | 0.928693356 |
| wavelet-LHH_ngtdm_Coarseness | 0.971623864 |
| wavelet-LHH_ngtdm_Complexity | 0.944886185 |
| wavelet-LHH_ngtdm_Contrast | 0.948564332 |
| wavelet-LHH_ngtdm_Strength | 0.971909937 |
| wavelet-HLL_firstorder_ 10Percentile | 0.690194939 |
| wavelet-HLL_firstorder_90Percentile | 0.791493426 |
| wavelet-HLL_firstorder_Energy | 0.917147242 |
| wavelet-HLL_firstorder_Entropy | 0.888139511 |
| wavelet-HLL_firstorder_InterquartileRange | 0.928501004 |
| wavelet-HLL_firstorder_Kurtosis | 0.708196437 |
| wavelet-HLL_firstorder_Maximum | 0.751113633 |
| wavelet-HLL_firstorder_MeanAbsoluteDeviation | 0.853828245 |

| wavelet-HLL_firstorder_Mean | 0.817124381 |
| --- | --- |
| wavelet-HLL_firstorder_Median | 0.950271627 |
| wavelet-HLL_firstorder_Minimum | 0.792652253 |
| wavelet-HLL_firstorder_Range | 0.797251234 |
| wavelet-HLL_firstorder_RobustMeanAbsoluteDeviation | 0.877308058 |
| wavelet-HLL_firstorder_RootMeanSquared | 0.838809982 |
| wavelet-HLL_firstorder_Skewness | 0.612528144 |
| wavelet-HLL_firstorder_TotalEnergy | 0.917147242 |
| wavelet-HLL_firstorder_Uniformity | 0.893973932 |
| wavelet-HLL_firstorder_Variance | 0.604854489 |
| wavelet-HLL_glcm_Autocorrelation | 0.126915212 |
| wavelet-HLL_glcm_ClusterProminence | 0.828637541 |
| wavelet-HLL_glcm_ClusterShade | 0.956492978 |
| wavelet-HLL_glcm_ClusterTendency | 0.800731115 |
| wavelet-HLL_glcm_Contrast | 0.968458908 |
| wavelet-HLL_glcm_Correlation | 0.923863547 |
| wavelet-HLL_glcm_DifferenceAverage | 0.968461295 |
| wavelet-HLL_glcm_DifferenceEntropy | 0.953486834 |
| wavelet-HLL_glcm_DifferenceVariance | 0.953908789 |
| wavelet-HLL_glcm_Id | 0.968461841 |
| wavelet-HLL_glcm_Idm | 0.968461637 |
| wavelet-HLL_glcm_Idmn | 0.754175641 |
| wavelet-HLL_glcm_Idn | 0.905217318 |
| wavelet-HLL_glcm_Imc1 | 0.957626893 |
| wavelet-HLL_glcm_Imc2 | 0.968020444 |
| wavelet-HLL_glcm_InverseVariance | 0.96846246 |
| wavelet-HLL_glcm_JointAverage | 0.210980191 |
| wavelet-HLL_glcm_JointEnergy | 0.883856214 |
| wavelet-HLL_glcm_JointEntropy | 0.881420438 |
| wavelet-HLL_glcm_MCC | 0.967180334 |
| wavelet-HLL_glcm_MaximumProbability | 0.926692457 |
| wavelet-HLL_glcm_SumAverage | 0.210980191 |
| wavelet-HLL_glcm_SumEntropy | 0.833470813 |
| wavelet-HLL_glcm_SumSquares | 0.853405103 |
| wavelet-HLL_gldm_DependenceEntropy | 0.916617137 |
| wavelet-HLL_gldm_DependenceNonUniformity | 0.935571552 |
| wavelet-HLL_gldm_DependenceNonUniformityNormalized | 0.863038045 |
| wavelet-HLL_gldm_DependenceVariance | 0.887971018 |
| wavelet-HLL_gldm_GrayLevelNonUniformity | 0.935437309 |
| wavelet-HLL_gldm_GrayLevelVariance | 0.893978455 |
| wavelet-HLL_gldm_HighGrayLevelEmphasis | 0.125200818 |
| wavelet-HLL_gldm_LargeDependenceEmphasis | 0.962718376 |
| wavelet-HLL_gldm_LargeDependenceHighGrayLevelEmphasis | 0.565422984 |
| wavelet-HLL_gldm_LargeDependenceLowGrayLevelEmphasis | 0.750507969 |
| wavelet-HLL_gldm_LowGrayLevelEmphasis | 0.319016314 |
| wavelet-HLL_gldm_SmallDependenceEmphasis | 0.932958534 |
| wavelet-HLL_gldm_SmallDependenceHighGrayLevelEmphasis | 0.842919039 |
| wavelet-HLL_gldm_SmallDependenceLowGrayLevelEmphasis | 0.937765766 |
| wavelet-HLL_glszm_GrayLevelNonUniformity | 0.560263599 |
| wavelet-HLL_glszm_GrayLevelNonUniformityNormalized | 0.506334642 |
| wavelet-HLL_glszm_GrayLevelVariance | 0.480167644 |
| wavelet-HLL_glszm_HighGrayLevelZoneEmphasis | 0.054285298 |
| wavelet-HLL_glszm_LargeAreaEmphasis | 0.934775688 |
| wavelet-HLL_glszm_LargeAreaHighGrayLevelEmphasis | 0.937605278 |
| wavelet-HLL_glszm_LargeAreaLowGrayLevelEmphasis | 0.921187147 |
| wavelet-HLL_glszm_LowGrayLevelZoneEmphasis | 0.290214816 |

| wavelet-HLL_glszm_SizeZoneNonUniformity | 0.250522202 |
| --- | --- |
| wavelet-HLL_glszm_SizeZoneNonUniformityNormalized | 0.267595361 |
| wavelet-HLL_glszm_SmallAreaEmphasis | 0.008716947 |
| wavelet-HLL_glszm_SmallAreaHighGrayLevelEmphasis | 0.096481536 |
| wavelet-HLL_glszm_SmallAreaLowGrayLevelEmphasis | 0.108530323 |
| wavelet-HLL_glszm_ZoneEntropy | 0.505739397 |
| wavelet-HLL_glszm_ZonePercentage | 0.920140469 |
| wavelet-HLL_glszm_ZoneVariance | 0.944745402 |
| wavelet-HLL_glrlm_GrayLevelNonUniformity | 0.931845474 |
| wavelet-HLL_glrlm_GrayLevelNonUniformityNormalized | 0.820395901 |
| wavelet-HLL_glrlm_GrayLevelVariance | 0.820289807 |
| wavelet-HLL_glrlm_HighGrayLevelRunEmphasis | 0.054088656 |
| wavelet-HLL_glrlm_LongRunEmphasis | 0.752555115 |
| wavelet-HLL_glrlm_LongRunHighGrayLevelEmphasis | 0.399035973 |
| wavelet-HLL_glrlm_LongRunLowGrayLevelEmphasis | 0.728217125 |
| wavelet-HLL_glrlm_LowGrayLevelRunEmphasis | 0.170748433 |
| wavelet-HLL_glrlm_RunEntropy | 0.64830141 |
| wavelet-HLL_glrlm_RunLengthNonUniformity | 0.913646022 |
| wavelet-HLL_glrlm_RunLengthNonUniformityNormalized | 0.323776843 |
| wavelet-HLL_glrlm_RunPercentage | 0.42617983 |
| wavelet-HLL_glrlm_RunVariance | 0.783646716 |
| wavelet-HLL_glrlm_ShortRunEmphasis | 0.287481088 |
| wavelet-HLL_glrlm_ShortRunHighGrayLevelEmphasis | 0.026689256 |
| wavelet-HLL_glrlm_ShortRunLowGrayLevelEmphasis | 0.151013771 |
| wavelet-HLL_ngtdm_Busyness | 0.759603313 |
| wavelet-HLL_ngtdm_Coarseness | 0.945496515 |
| wavelet-HLL_ngtdm_Complexity | 0.121710444 |
| wavelet-HLL_ngtdm_Contrast | 0.751063932 |
| wavelet-HLL_ngtdm_Strength | 0.94365783 |
| wavelet-HLH_firstorder_ 10Percentile | 0.949210389 |
| wavelet-HLH_firstorder_90Percentile | 0.947040436 |
| wavelet-HLH_firstorder_Energy | 0.97021528 |
| wavelet-HLH_firstorder_Entropy | 0.869458771 |
| wavelet-HLH_firstorder_InterquartileRange | 0.981518361 |
| wavelet-HLH_firstorder_Kurtosis | 0.626437424 |
| wavelet-HLH_firstorder_Maximum | 0.877697501 |
| wavelet-HLH_firstorder_MeanAbsoluteDeviation | 0.974536361 |
| wavelet-HLH_firstorder_Mean | 0.801524869 |
| wavelet-HLH_firstorder_Median | 0.816160046 |
| wavelet-HLH_firstorder_Minimum | 0.854581458 |
| wavelet-HLH_firstorder_Range | 0.87650687 |
| wavelet-HLH_firstorder_RobustMeanAbsoluteDeviation | 0.968381114 |
| wavelet-HLH_firstorder_RootMeanSquared | 0.95923498 |
| wavelet-HLH_firstorder_Skewness | 0.747909872 |
| wavelet-HLH_firstorder_TotalEnergy | 0.97021528 |
| wavelet-HLH_firstorder_Uniformity | 0.873432595 |
| wavelet-HLH_firstorder_Variance | 0.962524008 |
| wavelet-HLH_glcm_Autocorrelation | 0.996831617 |
| wavelet-HLH_glcm_ClusterProminence | 0.754213775 |
| wavelet-HLH_glcm_ClusterShade | 0.966750701 |
| wavelet-HLH_glcm_ClusterTendency | 0.570649638 |
| wavelet-HLH_glcm_Contrast | 0.863665057 |
| wavelet-HLH_glcm_Correlation | 0.783722188 |
| wavelet-HLH_glcm_DifferenceAverage | 0.863665057 |
| wavelet-HLH_glcm_DifferenceEntropy | 0.954864851 |
| wavelet-HLH_glcm_DifferenceVariance | 0.953494253 |

| wavelet-HLH_glcm_Id | 0.863665039 |
| --- | --- |
| wavelet-HLH_glcm_Idm | 0.863665046 |
| wavelet-HLH_glcm_Idmn | 0.919847837 |
| wavelet-HLH_glcm_Idn | 0.883856084 |
| wavelet-HLH_glcm_Imc1 | 0.912571953 |
| wavelet-HLH_glcm_Imc2 | 0.947596165 |
| wavelet-HLH_glcm_InverseVariance | 0.863665002 |
| wavelet-HLH_glcm_JointAverage | 0.993733348 |
| wavelet-HLH_glcm_JointEnergy | 0.937548291 |
| wavelet-HLH_glcm_JointEntropy | 0.914693026 |
| wavelet-HLH_glcm_MCC | 0.950916988 |
| wavelet-HLH_glcm_MaximumProbability | 0.965258272 |
| wavelet-HLH_glcm_SumAverage | 0.993733348 |
| wavelet-HLH_glcm_SumEntropy | 0.742495292 |
| wavelet-HLH_glcm_SumSquares | 0.9163835 |
| wavelet-HLH_gldm_DependenceEntropy | 0.895479083 |
| wavelet-HLH_gldm_DependenceNonUniformity | 0.934102577 |
| wavelet-HLH_gldm_DependenceNonUniformityNormalized | 0.851548219 |
| wavelet-HLH_gldm_DependenceVariance | 0.822016397 |
| wavelet-HLH_gldm_GrayLevelNonUniformity | 0.935468077 |
| wavelet-HLH_gldm_GrayLevelVariance | 0.873435067 |
| wavelet-HLH_gldm_HighGrayLevelEmphasis | 0.995333225 |
| wavelet-HLH_gldm_LargeDependenceEmphasis | 0.959382314 |
| wavelet-HLH_gldm_LargeDependenceHighGrayLevelEmphasis | 0.977820695 |
| wavelet-HLH_gldm_LargeDependenceLowGrayLevelEmphasis | 0.959406627 |
| wavelet-HLH_gldm_LowGrayLevelEmphasis | 0.981152915 |
| wavelet-HLH_gldm_SmallDependenceEmphasis | 0.944453942 |
| wavelet-HLH_gldm_SmallDependenceHighGrayLevelEmphasis | 0.945280234 |
| wavelet-HLH_gldm_SmallDependenceLowGrayLevelEmphasis | 0.924730073 |
| wavelet-HLH_glszm_GrayLevelNonUniformity | 0.56668516 |
| wavelet-HLH_glszm_GrayLevelNonUniformityNormalized | 0.192213445 |
| wavelet-HLH_glszm_GrayLevelVariance | 0.622290902 |
| wavelet-HLH_glszm_HighGrayLevelZoneEmphasis | 0.157548175 |
| wavelet-HLH_glszm_LargeAreaEmphasis | 0.793356924 |
| wavelet-HLH_glszm_LargeAreaHighGrayLevelEmphasis | 0.79652791 |
| wavelet-HLH_glszm_LargeAreaLowGrayLevelEmphasis | 0.790878307 |
| wavelet-HLH_glszm_LowGrayLevelZoneEmphasis | 0.027337484 |
| wavelet-HLH_glszm_SizeZoneNonUniformity | 0.387687427 |
| wavelet-HLH_glszm_SizeZoneNonUniformityNormalized | 0.392159964 |
| wavelet-HLH_glszm_SmallAreaEmphasis | 0.279183332 |
| wavelet-HLH_glszm_SmallAreaHighGrayLevelEmphasis | 0.213741711 |
| wavelet-HLH_glszm_SmallAreaLowGrayLevelEmphasis | 0.098979053 |
| wavelet-HLH_glszm_ZoneEntropy | 0.501543313 |
| wavelet-HLH_glszm_ZonePercentage | 0.959318102 |
| wavelet-HLH_glszm_ZoneVariance | 0.56028646 |
| wavelet-HLH_glrlm_GrayLevelNonUniformity | 0.936274472 |
| wavelet-HLH_glrlm_GrayLevelNonUniformityNormalized | 0.264352255 |
| wavelet-HLH_glrlm_GrayLevelVariance | 0.269790101 |
| wavelet-HLH_glrlm_HighGrayLevelRunEmphasis | 0.995349451 |
| wavelet-HLH_glrlm_LongRunEmphasis | 0.772602727 |
| wavelet-HLH_glrlm_LongRunHighGrayLevelEmphasis | 0.885183013 |
| wavelet-HLH_glrlm_LongRunLowGrayLevelEmphasis | 0.8109941 |
| wavelet-HLH_glrlm_LowGrayLevelRunEmphasis | 0.978915576 |
| wavelet-HLH_glrlm_RunEntropy | 0.463678614 |
| wavelet-HLH_glrlm_RunLengthNonUniformity | 0.934152199 |
| wavelet-HLH_glrlm_RunLengthNonUniformityNormalized | 0.32735632 |

| wavelet-HLH_glrlm_RunPercentage | 0.380615285 |
| --- | --- |
| wavelet-HLH_glrlm_RunVariance | 0.858018369 |
| wavelet-HLH_glrlm_ShortRunEmphasis | 0.223946195 |
| wavelet-HLH_glrlm_ShortRunHighGrayLevelEmphasis | 0.949802164 |
| wavelet-HLH_glrlm_ShortRunLowGrayLevelEmphasis | 0.605955086 |
| wavelet-HLH_ngtdm_Busyness | 0.816190312 |
| wavelet-HLH_ngtdm_Coarseness | 0.951894821 |
| wavelet-HLH_ngtdm_Complexity | 0.997792498 |
| wavelet-HLH_ngtdm_Contrast | 0.966744226 |
| wavelet-HLH_ngtdm_Strength | 0.951190906 |
| wavelet-HHL_firstorder_ 10Percentile | 0.974617295 |
| wavelet-HHL_firstorder_90Percentile | 0.943002908 |
| wavelet-HHL_firstorder_Energy | 0.751252672 |
| wavelet-HHL_firstorder_Entropy | 0.496656707 |
| wavelet-HHL_firstorder_InterquartileRange | 0.991254284 |
| wavelet-HHL_firstorder_Kurtosis | 0.709471273 |
| wavelet-HHL_firstorder_Maximum | 0.790922357 |
| wavelet-HHL_firstorder_MeanAbsoluteDeviation | 0.935980176 |
| wavelet-HHL_firstorder_Mean | 0.49961103 |
| wavelet-HHL_firstorder_Median | 0.770495496 |
| wavelet-HHL_firstorder_Minimum | 0.756833898 |
| wavelet-HHL_firstorder_Range | 0.785517733 |
| wavelet-HHL_firstorder_RobustMeanAbsoluteDeviation | 0.987907909 |
| wavelet-HHL_firstorder_RootMeanSquared | 0.875697319 |
| wavelet-HHL_firstorder_Skewness | 0.399414193 |
| wavelet-HHL_firstorder_TotalEnergy | 0.751252672 |
| wavelet-HHL_firstorder_Uniformity | 0.496966562 |
| wavelet-HHL_firstorder_Variance | 0.805190574 |
| wavelet-HHL_glcm_Autocorrelation | 0.594341991 |
| wavelet-HHL_glcm_ClusterProminence | 0.958125571 |
| wavelet-HHL_glcm_ClusterShade | 0.442557974 |
| wavelet-HHL_glcm_ClusterTendency | 0.959226589 |
| wavelet-HHL_glcm_Contrast | 0.956311157 |
| wavelet-HHL_glcm_Correlation | 0.957830739 |
| wavelet-HHL_glcm_DifferenceAverage | 0.956311157 |
| wavelet-HHL_glcm_DifferenceEntropy | 0.875110894 |
| wavelet-HHL_glcm_DifferenceVariance | 0.888824482 |
| wavelet-HHL_glcm_Id | 0.956311156 |
| wavelet-HHL_glcm_Idm | 0.956311156 |
| wavelet-HHL_glcm_Idmn | 0.956311155 |
| wavelet-HHL_glcm_Idn | 0.956311154 |
| wavelet-HHL_glcm_Imc1 | 0.873919926 |
| wavelet-HHL_glcm_Imc2 | 0.918283252 |
| wavelet-HHL_glcm_InverseVariance | 0.956311157 |
| wavelet-HHL_glcm_JointAverage | 0.517378301 |
| wavelet-HHL_glcm_JointEnergy | 0.90922058 |
| wavelet-HHL_glcm_JointEntropy | 0.897978229 |
| wavelet-HHL_glcm_MCC | 0.918064833 |
| wavelet-HHL_glcm_MaximumProbability | 0.913127147 |
| wavelet-HHL_glcm_SumAverage | 0.517378295 |
| wavelet-HHL_glcm_SumEntropy | 0.965686692 |
| wavelet-HHL_glcm_SumSquares | 0.645756958 |
| wavelet-HHL_gldm_DependenceEntropy | 0.907587853 |
| wavelet-HHL_gldm_DependenceNonUniformity | 0.941978602 |
| wavelet-HHL_gldm_DependenceNonUniformityNormalized | 0.911405539 |
| wavelet-HHL_gldm_DependenceVariance | 0.872311096 |

| wavelet-HHL_gldm_GrayLevelNonUniformity | 0.935481368 |
| --- | --- |
| wavelet-HHL_gldm_GrayLevelVariance | 0.49696632 |
| wavelet-HHL_gldm_HighGrayLevelEmphasis | 0.604626542 |
| wavelet-HHL_gldm_LargeDependenceEmphasis | 0.963923937 |
| wavelet-HHL_gldm_LargeDependenceHighGrayLevelEmphasis | 0.960085831 |
| wavelet-HHL_gldm_LargeDependenceLowGrayLevelEmphasis | 0.964876444 |
| wavelet-HHL_gldm_LowGrayLevelEmphasis | 0.604626538 |
| wavelet-HHL_gldm_SmallDependenceEmphasis | 0.925208477 |
| wavelet-HHL_gldm_SmallDependenceHighGrayLevelEmphasis | 0.895901274 |
| wavelet-HHL_gldm_SmallDependenceLowGrayLevelEmphasis | 0.931937133 |
| wavelet-HHL_glszm_GrayLevelNonUniformity | 0.403295033 |
| wavelet-HHL_glszm_GrayLevelNonUniformityNormalized | 0.135373163 |
| wavelet-HHL_glszm_GrayLevelVariance | 0.135373162 |
| wavelet-HHL_glszm_HighGrayLevelZoneEmphasis | 0.341594295 |
| wavelet-HHL_glszm_LargeAreaEmphasis | 0.635556491 |
| wavelet-HHL_glszm_LargeAreaHighGrayLevelEmphasis | 0.634763491 |
| wavelet-HHL_glszm_LargeAreaLowGrayLevelEmphasis | 0.636307155 |
| wavelet-HHL_glszm_LowGrayLevelZoneEmphasis | 0.341594295 |
| wavelet-HHL_glszm_SizeZoneNonUniformity | 0.27734346 |
| wavelet-HHL_glszm_SizeZoneNonUniformityNormalized | 0.079296782 |
| wavelet-HHL_glszm_SmallAreaEmphasis | 0.180345769 |
| wavelet-HHL_glszm_SmallAreaHighGrayLevelEmphasis | 0.057328555 |
| wavelet-HHL_glszm_SmallAreaLowGrayLevelEmphasis | 0.358671163 |
| wavelet-HHL_glszm_ZoneEntropy | 0.309544592 |
| wavelet-HHL_glszm_ZonePercentage | 0.847340426 |
| wavelet-HHL_glszm_ZoneVariance | 0.806707836 |
| wavelet-HHL_glrlm_GrayLevelNonUniformity | 0.934934007 |
| wavelet-HHL_glrlm_GrayLevelNonUniformityNormalized | 0.469368881 |
| wavelet-HHL_glrlm_GrayLevelVariance | 0.469368368 |
| wavelet-HHL_glrlm_HighGrayLevelRunEmphasis | 0.640190705 |
| wavelet-HHL_glrlm_LongRunEmphasis | 0.654887658 |
| wavelet-HHL_glrlm_LongRunHighGrayLevelEmphasis | 0.6548044 |
| wavelet-HHL_glrlm_LongRunLowGrayLevelEmphasis | 0.645906519 |
| wavelet-HHL_glrlm_LowGrayLevelRunEmphasis | 0.640190708 |
| wavelet-HHL_glrlm_RunEntropy | 0.623117654 |
| wavelet-HHL_glrlm_RunLengthNonUniformity | 0.92725245 |
| wavelet-HHL_glrlm_RunLengthNonUniformityNormalized | 0.400600087 |
| wavelet-HHL_glrlm_RunPercentage | 0.503471414 |
| wavelet-HHL_glrlm_RunVariance | 0.687143317 |
| wavelet-HHL_glrlm_ShortRunEmphasis | 0.409406918 |
| wavelet-HHL_glrlm_ShortRunHighGrayLevelEmphasis | 0.448147628 |
| wavelet-HHL_glrlm_ShortRunLowGrayLevelEmphasis | 0.372374553 |
| wavelet-HHL_ngtdm_Busyness | 0.924406241 |
| wavelet-HHL_ngtdm_Coarseness | 0.967918118 |
| wavelet-HHL_ngtdm_Complexity | 0.965224476 |
| wavelet-HHL_ngtdm_Contrast | 0.962391651 |
| wavelet-HHL_ngtdm_Strength | 0.968000569 |
| wavelet-HHH_firstorder_ 10Percentile | 0.964261923 |
| wavelet-HHH_firstorder_90Percentile | 0.964326551 |
| wavelet-HHH_firstorder_Energy | 0.885410459 |
| wavelet-HHH_firstorder_Entropy | 0.438947578 |
| wavelet-HHH_firstorder_InterquartileRange | 0.975974675 |
| wavelet-HHH_firstorder_Kurtosis | 0.347112301 |
| wavelet-HHH_firstorder_Maximum | 0.857794312 |
| wavelet-HHH_firstorder_MeanAbsoluteDeviation | 0.974315454 |
| wavelet-HHH_firstorder_Mean | 0.062266789 |

| wavelet-HHH_firstorder_Median | 0.402712314 |
| --- | --- |
| wavelet-HHH_firstorder_Minimum | 0.854252308 |
| wavelet-HHH_firstorder_Range | 0.862962283 |
| wavelet-HHH_firstorder_RobustMeanAbsoluteDeviation | 0.975390227 |
| wavelet-HHH_firstorder_RootMeanSquared | 0.952143597 |
| wavelet-HHH_firstorder_Skewness | 0.198207313 |
| wavelet-HHH_firstorder_TotalEnergy | 0.885410459 |
| wavelet-HHH_firstorder_Uniformity | 0.438958168 |
| wavelet-HHH_firstorder_Variance | 0.954821993 |
| wavelet-HHH_glcm_Autocorrelation | 0.76450876 |
| wavelet-HHH_glcm_ClusterProminence | 0.954308518 |
| wavelet-HHH_glcm_ClusterShade | 0.741452482 |
| wavelet-HHH_glcm_ClusterTendency | 0.954747806 |
| wavelet-HHH_glcm_Contrast | 0.960167005 |
| wavelet-HHH_glcm_Correlation | 0.957551124 |
| wavelet-HHH_glcm_DifferenceAverage | 0.960167005 |
| wavelet-HHH_glcm_DifferenceEntropy | 0.911852623 |
| wavelet-HHH_glcm_DifferenceVariance | 0.904894483 |
| wavelet-HHH_glcm_Id | 0.960167002 |
| wavelet-HHH_glcm_Idm | 0.960167002 |
| wavelet-HHH_glcm_Idmn | 0.960167002 |
| wavelet-HHH_glcm_Idn | 0.960167006 |
| wavelet-HHH_glcm_Imc1 | 0.910635116 |
| wavelet-HHH_glcm_Imc2 | 0.928519392 |
| wavelet-HHH_glcm_InverseVariance | 0.960167005 |
| wavelet-HHH_glcm_JointAverage | 0.774986841 |
| wavelet-HHH_glcm_JointEnergy | 0.892651246 |
| wavelet-HHH_glcm_JointEntropy | 0.899883164 |
| wavelet-HHH_glcm_MCC | 0.928422451 |
| wavelet-HHH_glcm_MaximumProbability | 0.904421111 |
| wavelet-HHH_glcm_SumAverage | 0.774986828 |
| wavelet-HHH_glcm_SumEntropy | 0.932876855 |
| wavelet-HHH_glcm_SumSquares | 0.506618289 |
| wavelet-HHH_gldm_DependenceEntropy | 0.85779872 |
| wavelet-HHH_gldm_DependenceNonUniformity | 0.938226039 |
| wavelet-HHH_gldm_DependenceNonUniformityNormalized | 0.849381243 |
| wavelet-HHH_gldm_DependenceVariance | 0.813855348 |
| wavelet-HHH_gldm_GrayLevelNonUniformity | 0.935426587 |
| wavelet-HHH_gldm_GrayLevelVariance | 0.438958086 |
| wavelet-HHH_gldm_HighGrayLevelEmphasis | 0.592155692 |
| wavelet-HHH_gldm_LargeDependenceEmphasis | 0.967773926 |
| wavelet-HHH_gldm_LargeDependenceHighGrayLevelEmphasis | 0.968957552 |
| wavelet-HHH_gldm_LargeDependenceLowGrayLevelEmphasis | 0.964696419 |
| wavelet-HHH_gldm_LowGrayLevelEmphasis | 0.592155711 |
| wavelet-HHH_gldm_SmallDependenceEmphasis | 0.954669451 |
| wavelet-HHH_gldm_SmallDependenceHighGrayLevelEmphasis | 0.960770654 |
| wavelet-HHH_gldm_SmallDependenceLowGrayLevelEmphasis | 0.939269818 |
| wavelet-HHH_glszm_GrayLevelNonUniformity | 0.391650844 |
| wavelet-HHH_glszm_GrayLevelNonUniformityNormalized | 0.22091391 |
| wavelet-HHH_glszm_GrayLevelVariance | 0.22091391 |
| wavelet-HHH_glszm_HighGrayLevelZoneEmphasis | 0.237097401 |
| wavelet-HHH_glszm_LargeAreaEmphasis | 0.933370879 |
| wavelet-HHH_glszm_LargeAreaHighGrayLevelEmphasis | 0.936408108 |
| wavelet-HHH_glszm_LargeAreaLowGrayLevelEmphasis | 0.930165985 |
| wavelet-HHH_glszm_LowGrayLevelZoneEmphasis | 0.237097401 |
| wavelet-HHH_glszm_SizeZoneNonUniformity | 0.044854899 |

| wavelet-HHH_glszm_SizeZoneNonUniformityNormalized | 0.444533669 |
| --- | --- |
| wavelet-HHH_glszm_SmallAreaEmphasis | 0.131907506 |
| wavelet-HHH_glszm_SmallAreaHighGrayLevelEmphasis | 0.093108576 |
| wavelet-HHH_glszm_SmallAreaLowGrayLevelEmphasis | 0.176520456 |
| wavelet-HHH_glszm_ZoneEntropy | 0.515067383 |
| wavelet-HHH_glszm_ZonePercentage | 0.879225726 |
| wavelet-HHH_glszm_ZoneVariance | 0.834660424 |
| wavelet-HHH_glrlm_GrayLevelNonUniformity | 0.935583436 |
| wavelet-HHH_glrlm_GrayLevelNonUniformityNormalized | 0.494048891 |
| wavelet-HHH_glrlm_GrayLevelVariance | 0.494050087 |
| wavelet-HHH_glrlm_HighGrayLevelRunEmphasis | 0.232521098 |
| wavelet-HHH_glrlm_LongRunEmphasis | 0.603847913 |
| wavelet-HHH_glrlm_LongRunHighGrayLevelEmphasis | 0.56948994 |
| wavelet-HHH_glrlm_LongRunLowGrayLevelEmphasis | 0.639902354 |
| wavelet-HHH_glrlm_LowGrayLevelRunEmphasis | 0.232521117 |
| wavelet-HHH_glrlm_RunEntropy | 0.613060011 |
| wavelet-HHH_glrlm_RunLengthNonUniformity | 0.931524921 |
| wavelet-HHH_glrlm_RunLengthNonUniformityNormalized | 0.447555453 |
| wavelet-HHH_glrlm_RunPercentage | 0.516590711 |
| wavelet-HHH_glrlm_RunVariance | 0.678703769 |
| wavelet-HHH_glrlm_ShortRunEmphasis | 0.375057473 |
| wavelet-HHH_glrlm_ShortRunHighGrayLevelEmphasis | 0.449620917 |
| wavelet-HHH_glrlm_ShortRunLowGrayLevelEmphasis | 0.295133671 |
| wavelet-HHH_ngtdm_Busyness | 0.923077701 |
| wavelet-HHH_ngtdm_Coarseness | 0.964390899 |
| wavelet-HHH_ngtdm_Complexity | 0.894275219 |
| wavelet-HHH_ngtdm_Contrast | 0.892631198 |
| wavelet-HHH_ngtdm_Strength | 0.964428474 |
| wavelet-LLL_firstorder_ 10Percentile | 0.70069462 |
| wavelet-LLL_firstorder_90Percentile | 0.998705893 |
| wavelet-LLL_firstorder_Energy | 0.990812495 |
| wavelet-LLL_firstorder_Entropy | 0.881818326 |
| wavelet-LLL_firstorder_InterquartileRange | 0.934742879 |
| wavelet-LLL_firstorder_Kurtosis | 0.483563436 |
| wavelet-LLL_firstorder_Maximum | 0.997079354 |
| wavelet-LLL_firstorder_MeanAbsoluteDeviation | 0.88228299 |
| wavelet-LLL_firstorder_Mean | 0.969395969 |
| wavelet-LLL_firstorder_Median | 0.981007296 |
| wavelet-LLL_firstorder_Minimum | 0.442910661 |
| wavelet-LLL_firstorder_Range | 0.810001724 |
| wavelet-LLL_firstorder_RobustMeanAbsoluteDeviation | 0.916543761 |
| wavelet-LLL_firstorder_RootMeanSquared | 0.982799809 |
| wavelet-LLL_firstorder_Skewness | 0.635985685 |
| wavelet-LLL_firstorder_TotalEnergy | 0.990812495 |
| wavelet-LLL_firstorder_Uniformity | 0.891381244 |
| wavelet-LLL_firstorder_Variance | 0.836117391 |
| wavelet-LLL_glcm_Autocorrelation | 0.490718812 |
| wavelet-LLL_glcm_ClusterProminence | 0.599896575 |
| wavelet-LLL_glcm_ClusterShade | 0.444001986 |
| wavelet-LLL_glcm_ClusterTendency | 0.839227388 |
| wavelet-LLL_glcm_Contrast | 0.928934708 |
| wavelet-LLL_glcm_Correlation | 0.67529576 |
| wavelet-LLL_glcm_DifferenceAverage | 0.953704878 |
| wavelet-LLL_glcm_DifferenceEntropy | 0.889731013 |
| wavelet-LLL_glcm_DifferenceVariance | 0.837679372 |
| wavelet-LLL_glcm_Id | 0.957754336 |

| wavelet-LLL_glcm_Idm | 0.956643423 |
| --- | --- |
| wavelet-LLL_glcm_Idmn | 0.824964951 |
| wavelet-LLL_glcm_Idn | 0.919844861 |
| wavelet-LLL_glcm_Imc1 | 0.642963872 |
| wavelet-LLL_glcm_Imc2 | 0.748898514 |
| wavelet-LLL_glcm_InverseVariance | 0.953290903 |
| wavelet-LLL_glcm_JointAverage | 0.522480007 |
| wavelet-LLL_glcm_JointEnergy | 0.925687929 |
| wavelet-LLL_glcm_JointEntropy | 0.931520832 |
| wavelet-LLL_glcm_MCC | 0.575286474 |
| wavelet-LLL_glcm_MaximumProbability | 0.944566289 |
| wavelet-LLL_glcm_SumAverage | 0.522480007 |
| wavelet-LLL_glcm_SumEntropy | 0.908246642 |
| wavelet-LLL_glcm_SumSquares | 0.88660924 |
| wavelet-LLL_gldm_DependenceEntropy | 0.946668178 |
| wavelet-LLL_gldm_DependenceNonUniformity | 0.954845437 |
| wavelet-LLL_gldm_DependenceNonUniformityNormalized | 0.929689603 |
| wavelet-LLL_gldm_DependenceVariance | 0.936913547 |
| wavelet-LLL_gldm_GrayLevelNonUniformity | 0.975800098 |
| wavelet-LLL_gldm_GrayLevelVariance | 0.86301848 |
| wavelet-LLL_gldm_HighGrayLevelEmphasis | 0.47691112 |
| wavelet-LLL_gldm_LargeDependenceEmphasis | 0.989910596 |
| wavelet-LLL_gldm_LargeDependenceHighGrayLevelEmphasis | 0.617715139 |
| wavelet-LLL_gldm_LargeDependenceLowGrayLevelEmphasis | 0.603971027 |
| wavelet-LLL_gldm_LowGrayLevelEmphasis | 0.654881323 |
| wavelet-LLL_gldm_SmallDependenceEmphasis | 0.912155057 |
| wavelet-LLL_gldm_SmallDependenceHighGrayLevelEmphasis | 0.63949594 |
| wavelet-LLL_gldm_SmallDependenceLowGrayLevelEmphasis | 0.745000398 |
| wavelet-LLL_glszm_GrayLevelNonUniformity | 0.623131547 |
| wavelet-LLL_glszm_GrayLevelNonUniformityNormalized | 0.411825481 |
| wavelet-LLL_glszm_GrayLevelVariance | 0.781778947 |
| wavelet-LLL_glszm_HighGrayLevelZoneEmphasis | 0.429504061 |
| wavelet-LLL_glszm_LargeAreaEmphasis | 0.882695565 |
| wavelet-LLL_glszm_LargeAreaHighGrayLevelEmphasis | 0.510665603 |
| wavelet-LLL_glszm_LargeAreaLowGrayLevelEmphasis | 0.895698747 |
| wavelet-LLL_glszm_LowGrayLevelZoneEmphasis | 0.168467849 |
| wavelet-LLL_glszm_SizeZoneNonUniformity | 0.647839718 |
| wavelet-LLL_glszm_SizeZoneNonUniformityNormalized | 0.216306039 |
| wavelet-LLL_glszm_SmallAreaEmphasis | 0.093519738 |
| wavelet-LLL_glszm_SmallAreaHighGrayLevelEmphasis | 0.593547615 |
| wavelet-LLL_glszm_SmallAreaLowGrayLevelEmphasis | 0.1591115 |
| wavelet-LLL_glszm_ZoneEntropy | 0.703253053 |
| wavelet-LLL_glszm_ZonePercentage | 0.883742298 |
| wavelet-LLL_glszm_ZoneVariance | 0.892055723 |
| wavelet-LLL_glrlm_GrayLevelNonUniformity | 0.979648194 |
| wavelet-LLL_glrlm_GrayLevelNonUniformityNormalized | 0.696654714 |
| wavelet-LLL_glrlm_GrayLevelVariance | 0.810777423 |
| wavelet-LLL_glrlm_HighGrayLevelRunEmphasis | 0.456859572 |
| wavelet-LLL_glrlm_LongRunEmphasis | 0.969843514 |
| wavelet-LLL_glrlm_LongRunHighGrayLevelEmphasis | 0.714525697 |
| wavelet-LLL_glrlm_LongRunLowGrayLevelEmphasis | 0.609339448 |
| wavelet-LLL_glrlm_LowGrayLevelRunEmphasis | 0.56589613 |
| wavelet-LLL_glrlm_RunEntropy | 0.815127562 |
| wavelet-LLL_glrlm_RunLengthNonUniformity | 0.930902677 |
| wavelet-LLL_glrlm_RunLengthNonUniformityNormalized | 0.80729797 |
| wavelet-LLL_glrlm_RunPercentage | 0.829551518 |

| wavelet-LLL_glrlm_RunVariance | 0.957386747 |
| --- | --- |
| wavelet-LLL_glrlm_ShortRunEmphasis | 0.822788263 |
| wavelet-LLL_glrlm_ShortRunHighGrayLevelEmphasis | 0.598647663 |
| wavelet-LLL_glrlm_ShortRunLowGrayLevelEmphasis | 0.501674752 |
| wavelet-LLL_ngtdm_Busyness | 0.603384493 |
| wavelet-LLL_ngtdm_Coarseness | 0.10593232 |
| wavelet-LLL_ngtdm_Complexity | 0.8286486 |
| wavelet-LLL_ngtdm_Contrast | 0.761107225 |
| wavelet-LLL_ngtdm_Strength | 0.464038318 |
