# Supplementary material for: Deep learning–based automatic segmentation of meningioma from T1-weighted contrast-enhanced MRI for preoperative meningioma differentiation using radiomic features
Source: BMC Med Imaging. 2024 Mar 5;24:56. doi: 10.1186/s12880-024-01218-3 (PMC10916038; doi:10.1186/s12880-024-01218-3)
Supplement: Supplementary file 2 — Additional file 2: Supplementary Material 2. All radiomics features with both inter-ICC and intra-ICC values exceeding 0.80. [file 12880_2024_1218_MOESM2_ESM.docx]

Supplementaty Material 2 All radiomics features with both inter-ICC and intra-ICC values exceeding 0.80

Feature name

0 original_shape_Elongation

1 original_shape_Flatness

2 original_shape_LeastAxisLength

3 original_shape_MajorAxisLength

4 original_shape_Maximum2DDiameterColumn

5 original_shape_Maximum2DDiameterRow

6 original_shape_Maximum2DDiameterSlice

7 original_shape_Maximum3DDiameter

8 original_shape_MeshVolume

9 original_shape_MinorAxisLength

10 original_shape_Sphericity

11 original_shape_SurfaceArea

12 original_shape_SurfaceVolumeRatio

13 original_shape_VoxelVolume

14 original_firstorder_10Percentile

15 original_firstorder_90Percentile

16 original_firstorder_Energy

17 original_firstorder_Entropy

18 original_firstorder_InterquartileRange

19 original_firstorder_Kurtosis

20 original_firstorder_Maximum

21 original_firstorder_MeanAbsoluteDeviation

22 original_firstorder_Mean

23 original_firstorder_Median

24 original_firstorder_Minimum

25 original_firstorder_Range

26 original_firstorder_RobustMeanAbsoluteDeviation

27 original_firstorder_RootMeanSquared

28 original_firstorder_Skewness

29 original_firstorder_TotalEnergy

30 original_firstorder_Uniformity

31 original_firstorder_Variance

32 original_glcm_ClusterProminence

33 original_glcm_ClusterShade

34 original_glcm_ClusterTendency

35 original_glcm_Contrast

36 original_glcm_DifferenceAverage

37 original_glcm_DifferenceEntropy

38 original_glcm_DifferenceVariance

39 original_glcm_Id

40 original_glcm_Idm

41 original_glcm_Idmn

42 original_glcm_Idn

43 original_glcm_Imc1

44 original_glcm_Imc2

45 original_glcm_InverseVariance

46 original_glcm_JointEnergy

47 original_glcm_JointEntropy

48 original_glcm_MaximumProbability

49 original_glcm_SumEntropy

50 original_glcm_SumSquares

51 original_gldm_DependenceEntropy

52 original_gldm_DependenceNonUniformity

53 original_gldm_DependenceNonUniformityNormalized

54 original_gldm_DependenceVariance

55 original_gldm_GrayLevelNonUniformity

56 original_gldm_GrayLevelVariance

57 original_gldm_LargeDependenceEmphasis

58 original_gldm_SmallDependenceEmphasis

59 original_glszm_GrayLevelNonUniformity

60 original_glszm_HighGrayLevelZoneEmphasis

61 original_glszm_LargeAreaEmphasis

62 original_glszm_LargeAreaHighGrayLevelEmphasis

63 original_glszm_ZoneEntropy

64 original_glszm_ZonePercentage

65 original_glrlm_GrayLevelNonUniformity

66 original_glrlm_GrayLevelNonUniformityNormalized

67 original_glrlm_GrayLevelVariance

68 original_glrlm_LongRunEmphasis

69 original_glrlm_RunEntropy

70 original_glrlm_RunLengthNonUniformity

71 original_glrlm_RunLengthNonUniformityNormalized

72 original_glrlm_RunPercentage

73 original_glrlm_RunVariance

74 original_glrlm_ShortRunEmphasis

75 original_ngtdm_Busyness

76 original_ngtdm_Complexity

77 original_ngtdm_Contrast

78 exponential_firstorder_10Percentile

79 exponential_firstorder_90Percentile

80 exponential_firstorder_Energy

81 exponential_firstorder_Entropy

82 exponential_firstorder_InterquartileRange

83 exponential_firstorder_Kurtosis

84 exponential_firstorder_Maximum

85 exponential_firstorder_MeanAbsoluteDeviation

86 exponential_firstorder_Mean

87 exponential_firstorder_Median

88 exponential_firstorder_Range

89 exponential_firstorder_RobustMeanAbsoluteDeviation

90 exponential_firstorder_RootMeanSquared

91 exponential_firstorder_Skewness

92 exponential_firstorder_TotalEnergy

93 exponential_firstorder_Uniformity

94 exponential_firstorder_Variance

95 exponential_glcm_Autocorrelation

96 exponential_glcm_ClusterProminence

97 exponential_glcm_ClusterShade

98 exponential_glcm_ClusterTendency

99 exponential_glcm_Contrast

100 exponential_glcm_Correlation

101 exponential_glcm_DifferenceAverage

102 exponential_glcm_DifferenceEntropy

103 exponential_glcm_DifferenceVariance

104 exponential_glcm_Id

105 exponential_glcm_Idm

106 exponential_glcm_Idmn

107 exponential_glcm_Idn

108 exponential_glcm_Imc1

109 exponential_glcm_Imc2

110 exponential_glcm_InverseVariance

111 exponential_glcm_JointAverage

112 exponential_glcm_JointEnergy

113 exponential_glcm_JointEntropy

114 exponential_glcm_MCC

115 exponential_glcm_MaximumProbability

116 exponential_glcm_SumAverage

117 exponential_glcm_SumEntropy

118 exponential_glcm_SumSquares

119 exponential_gldm_DependenceEntropy

120 exponential_gldm_DependenceNonUniformity

121 exponential_gldm_DependenceNonUniformityNormalized

122 exponential_gldm_DependenceVariance

123 exponential_gldm_GrayLevelNonUniformity

124 exponential_gldm_GrayLevelVariance

125 exponential_gldm_HighGrayLevelEmphasis

126 exponential_gldm_LargeDependenceEmphasis

127 exponential_gldm_LargeDependenceHighGrayLevelEmphasis

128 exponential_gldm_LargeDependenceLowGrayLevelEmphasis

129 exponential_gldm_LowGrayLevelEmphasis

130 exponential_gldm_SmallDependenceEmphasis

131 exponential_gldm_SmallDependenceHighGrayLevelEmphasis

132 exponential_gldm_SmallDependenceLowGrayLevelEmphasis

133 exponential_glszm_GrayLevelNonUniformity

134 exponential_glszm_GrayLevelNonUniformityNormalized

135 exponential_glszm_GrayLevelVariance

136 exponential_glszm_HighGrayLevelZoneEmphasis

137 exponential_glszm_LargeAreaEmphasis

138 exponential_glszm_LargeAreaHighGrayLevelEmphasis

139 exponential_glszm_LargeAreaLowGrayLevelEmphasis

140 exponential_glszm_LowGrayLevelZoneEmphasis

141 exponential_glszm_SizeZoneNonUniformity

142 exponential_glszm_SizeZoneNonUniformityNormalized

143 exponential_glszm_SmallAreaHighGrayLevelEmphasis

144 exponential_glszm_ZoneEntropy

145 exponential_glszm_ZonePercentage

146 exponential_glrlm_GrayLevelNonUniformity

147 exponential_glrlm_GrayLevelNonUniformityNormalized

148 exponential_glrlm_GrayLevelVariance

149 exponential_glrlm_HighGrayLevelRunEmphasis

150 exponential_glrlm_LongRunEmphasis

151 exponential_glrlm_LongRunHighGrayLevelEmphasis

152 exponential_glrlm_LongRunLowGrayLevelEmphasis

153 exponential_glrlm_LowGrayLevelRunEmphasis

154 exponential_glrlm_RunEntropy

155 exponential_glrlm_RunLengthNonUniformity

156 exponential_glrlm_RunLengthNonUniformityNormalized

157 exponential_glrlm_RunPercentage

158 exponential_glrlm_RunVariance

159 exponential_glrlm_ShortRunEmphasis

160 exponential_glrlm_ShortRunHighGrayLevelEmphasis

161 exponential_glrlm_ShortRunLowGrayLevelEmphasis

162 exponential_ngtdm_Busyness

163 exponential_ngtdm_Coarseness

164 exponential_ngtdm_Complexity

165 exponential_ngtdm_Contrast

166 exponential_ngtdm_Strength

167 gradient_firstorder_10Percentile

168 gradient_firstorder_90Percentile

169 gradient_firstorder_Energy

170 gradient_firstorder_InterquartileRange

171 gradient_firstorder_Kurtosis

172 gradient_firstorder_Maximum

173 gradient_firstorder_MeanAbsoluteDeviation

174 gradient_firstorder_Mean

175 gradient_firstorder_Median

176 gradient_firstorder_Minimum

177 gradient_firstorder_Range

178 gradient_firstorder_RobustMeanAbsoluteDeviation

179 gradient_firstorder_RootMeanSquared

180 gradient_firstorder_Skewness

181 gradient_firstorder_TotalEnergy

182 gradient_firstorder_Uniformity

183 gradient_firstorder_Variance

184 gradient_glcm_Autocorrelation

185 gradient_glcm_ClusterProminence

186 gradient_glcm_ClusterShade

187 gradient_glcm_ClusterTendency

188 gradient_glcm_Contrast

189 gradient_glcm_Correlation

190 gradient_glcm_DifferenceAverage

191 gradient_glcm_DifferenceEntropy

192 gradient_glcm_DifferenceVariance

193 gradient_glcm_Id

194 gradient_glcm_Idm

195 gradient_glcm_Idmn

196 gradient_glcm_Idn

197 gradient_glcm_Imc1

198 gradient_glcm_Imc2

199 gradient_glcm_InverseVariance

200 gradient_glcm_JointAverage

201 gradient_glcm_JointEnergy

202 gradient_glcm_JointEntropy

203 gradient_glcm_MCC

204 gradient_glcm_MaximumProbability

205 gradient_glcm_SumAverage

206 gradient_glcm_SumEntropy

207 gradient_glcm_SumSquares

208 gradient_gldm_DependenceEntropy

209 gradient_gldm_DependenceNonUniformity

210 gradient_gldm_DependenceNonUniformityNormalized

211 gradient_gldm_DependenceVariance

212 gradient_gldm_GrayLevelNonUniformity

213 gradient_gldm_GrayLevelVariance

214 gradient_gldm_HighGrayLevelEmphasis

215 gradient_gldm_LargeDependenceEmphasis

216 gradient_gldm_LargeDependenceHighGrayLevelEmphasis

217 gradient_gldm_LargeDependenceLowGrayLevelEmphasis

218 gradient_gldm_LowGrayLevelEmphasis

219 gradient_gldm_SmallDependenceEmphasis

220 gradient_gldm_SmallDependenceHighGrayLevelEmphasis

221 gradient_gldm_SmallDependenceLowGrayLevelEmphasis

222 gradient_glszm_GrayLevelNonUniformityNormalized

223 gradient_glszm_GrayLevelVariance

224 gradient_glszm_HighGrayLevelZoneEmphasis

225 gradient_glszm_LargeAreaEmphasis

226 gradient_glszm_LargeAreaHighGrayLevelEmphasis

227 gradient_glszm_LargeAreaLowGrayLevelEmphasis

228 gradient_glszm_LowGrayLevelZoneEmphasis

229 gradient_glszm_SizeZoneNonUniformity

230 gradient_glszm_ZonePercentage

231 gradient_glrlm_GrayLevelNonUniformity

232 gradient_glrlm_GrayLevelNonUniformityNormalized

233 gradient_glrlm_GrayLevelVariance

234 gradient_glrlm_HighGrayLevelRunEmphasis

235 gradient_glrlm_LongRunEmphasis

236 gradient_glrlm_LongRunHighGrayLevelEmphasis

237 gradient_glrlm_LongRunLowGrayLevelEmphasis

238 gradient_glrlm_LowGrayLevelRunEmphasis

239 gradient_glrlm_RunEntropy

240 gradient_glrlm_RunLengthNonUniformity

241 gradient_glrlm_RunLengthNonUniformityNormalized

242 gradient_glrlm_RunPercentage

243 gradient_glrlm_RunVariance

244 gradient_glrlm_ShortRunEmphasis

245 gradient_glrlm_ShortRunHighGrayLevelEmphasis

246 gradient_glrlm_ShortRunLowGrayLevelEmphasis

247 gradient_ngtdm_Busyness

248 gradient_ngtdm_Coarseness

249 gradient_ngtdm_Complexity

250 gradient_ngtdm_Contrast

251 gradient_ngtdm_Strength

252 lbp-2D_firstorder_10Percentile

253 lbp-2D_firstorder_90Percentile

254 lbp-2D_firstorder_Energy

255 lbp-2D_firstorder_Entropy

256 lbp-2D_firstorder_InterquartileRange

257 lbp-2D_firstorder_Kurtosis

258 lbp-2D_firstorder_Maximum

259 lbp-2D_firstorder_MeanAbsoluteDeviation

260 lbp-2D_firstorder_Mean

261 lbp-2D_firstorder_Median

262 lbp-2D_firstorder_Minimum

263 lbp-2D_firstorder_Range

264 lbp-2D_firstorder_RobustMeanAbsoluteDeviation

265 lbp-2D_firstorder_RootMeanSquared

266 lbp-2D_firstorder_Skewness

267 lbp-2D_firstorder_TotalEnergy

268 lbp-2D_firstorder_Uniformity

269 lbp-2D_firstorder_Variance

270 lbp-2D_glcm_Autocorrelation

271 lbp-2D_glcm_ClusterProminence

272 lbp-2D_glcm_ClusterShade

273 lbp-2D_glcm_ClusterTendency

274 lbp-2D_glcm_Contrast

275 lbp-2D_glcm_Correlation

276 lbp-2D_glcm_DifferenceAverage

277 lbp-2D_glcm_DifferenceEntropy

278 lbp-2D_glcm_DifferenceVariance

279 lbp-2D_glcm_Id

280 lbp-2D_glcm_Idm

281 lbp-2D_glcm_Idmn

282 lbp-2D_glcm_Idn

283 lbp-2D_glcm_Imc1

284 lbp-2D_glcm_Imc2

285 lbp-2D_glcm_InverseVariance

286 lbp-2D_glcm_JointAverage

287 lbp-2D_glcm_JointEnergy

288 lbp-2D_glcm_JointEntropy

289 lbp-2D_glcm_MCC

290 lbp-2D_glcm_MaximumProbability

291 lbp-2D_glcm_SumAverage

292 lbp-2D_glcm_SumEntropy

293 lbp-2D_glcm_SumSquares

294 lbp-2D_gldm_DependenceEntropy

295 lbp-2D_gldm_DependenceNonUniformity

296 lbp-2D_gldm_DependenceNonUniformityNormalized

297 lbp-2D_gldm_DependenceVariance

298 lbp-2D_gldm_GrayLevelNonUniformity

299 lbp-2D_gldm_GrayLevelVariance

300 lbp-2D_gldm_HighGrayLevelEmphasis

301 lbp-2D_gldm_LargeDependenceEmphasis

302 lbp-2D_gldm_LargeDependenceHighGrayLevelEmphasis

303 lbp-2D_gldm_LargeDependenceLowGrayLevelEmphasis

304 lbp-2D_gldm_LowGrayLevelEmphasis

305 lbp-2D_gldm_SmallDependenceEmphasis

306 lbp-2D_gldm_SmallDependenceHighGrayLevelEmphasis

307 lbp-2D_gldm_SmallDependenceLowGrayLevelEmphasis

308 lbp-2D_glszm_GrayLevelNonUniformity

309 lbp-2D_glszm_LargeAreaEmphasis

310 lbp-2D_glszm_LargeAreaHighGrayLevelEmphasis

311 lbp-2D_glszm_LargeAreaLowGrayLevelEmphasis

312 lbp-2D_glszm_SizeZoneNonUniformity

313 lbp-2D_glszm_SizeZoneNonUniformityNormalized

314 lbp-2D_glszm_SmallAreaEmphasis

315 lbp-2D_glszm_SmallAreaHighGrayLevelEmphasis

316 lbp-2D_glszm_SmallAreaLowGrayLevelEmphasis

317 lbp-2D_glszm_ZonePercentage

318 lbp-2D_glszm_ZoneVariance

319 lbp-2D_glrlm_GrayLevelNonUniformity

320 lbp-2D_glrlm_GrayLevelNonUniformityNormalized

321 lbp-2D_glrlm_GrayLevelVariance

322 lbp-2D_glrlm_HighGrayLevelRunEmphasis

323 lbp-2D_glrlm_LongRunEmphasis

324 lbp-2D_glrlm_LongRunHighGrayLevelEmphasis

325 lbp-2D_glrlm_LongRunLowGrayLevelEmphasis

326 lbp-2D_glrlm_LowGrayLevelRunEmphasis

327 lbp-2D_glrlm_RunEntropy

328 lbp-2D_glrlm_RunLengthNonUniformity

329 lbp-2D_glrlm_RunLengthNonUniformityNormalized

330 lbp-2D_glrlm_RunPercentage

331 lbp-2D_glrlm_RunVariance

332 lbp-2D_glrlm_ShortRunEmphasis

333 lbp-2D_glrlm_ShortRunHighGrayLevelEmphasis

334 lbp-2D_glrlm_ShortRunLowGrayLevelEmphasis

335 lbp-2D_ngtdm_Busyness

336 lbp-2D_ngtdm_Coarseness

337 lbp-2D_ngtdm_Complexity

338 lbp-2D_ngtdm_Contrast

339 lbp-2D_ngtdm_Strength

340 lbp-3D-m1_firstorder_10Percentile

341 lbp-3D-m1_firstorder_90Percentile

342 lbp-3D-m1_firstorder_Energy

343 lbp-3D-m1_firstorder_Entropy

344 lbp-3D-m1_firstorder_InterquartileRange

345 lbp-3D-m1_firstorder_Kurtosis

346 lbp-3D-m1_firstorder_Maximum

347 lbp-3D-m1_firstorder_MeanAbsoluteDeviation

348 lbp-3D-m1_firstorder_Mean

349 lbp-3D-m1_firstorder_Median

350 lbp-3D-m1_firstorder_Minimum

351 lbp-3D-m1_firstorder_Range

352 lbp-3D-m1_firstorder_RobustMeanAbsoluteDeviation

353 lbp-3D-m1_firstorder_RootMeanSquared

354 lbp-3D-m1_firstorder_Skewness

355 lbp-3D-m1_firstorder_TotalEnergy

356 lbp-3D-m1_firstorder_Uniformity

357 lbp-3D-m1_firstorder_Variance

358 lbp-3D-m1_glcm_Autocorrelation

359 lbp-3D-m1_glcm_ClusterProminence

360 lbp-3D-m1_glcm_ClusterShade

361 lbp-3D-m1_glcm_ClusterTendency

362 lbp-3D-m1_glcm_Contrast

363 lbp-3D-m1_glcm_Correlation

364 lbp-3D-m1_glcm_DifferenceAverage

365 lbp-3D-m1_glcm_DifferenceEntropy

366 lbp-3D-m1_glcm_DifferenceVariance

367 lbp-3D-m1_glcm_Id

368 lbp-3D-m1_glcm_Idm

369 lbp-3D-m1_glcm_Idmn

370 lbp-3D-m1_glcm_Idn

371 lbp-3D-m1_glcm_Imc1

372 lbp-3D-m1_glcm_Imc2

373 lbp-3D-m1_glcm_InverseVariance

374 lbp-3D-m1_glcm_JointAverage

375 lbp-3D-m1_glcm_JointEnergy

376 lbp-3D-m1_glcm_JointEntropy

377 lbp-3D-m1_glcm_MCC

378 lbp-3D-m1_glcm_MaximumProbability

379 lbp-3D-m1_glcm_SumAverage

380 lbp-3D-m1_glcm_SumEntropy

381 lbp-3D-m1_glcm_SumSquares

382 lbp-3D-m1_gldm_DependenceEntropy

383 lbp-3D-m1_gldm_DependenceNonUniformity

384 lbp-3D-m1_gldm_DependenceNonUniformityNormalized

385 lbp-3D-m1_gldm_DependenceVariance

386 lbp-3D-m1_gldm_GrayLevelNonUniformity

387 lbp-3D-m1_gldm_GrayLevelVariance

388 lbp-3D-m1_gldm_HighGrayLevelEmphasis

389 lbp-3D-m1_gldm_LargeDependenceEmphasis

390 lbp-3D-m1_gldm_LargeDependenceHighGrayLevelEmphasis

391 lbp-3D-m1_gldm_LargeDependenceLowGrayLevelEmphasis

392 lbp-3D-m1_gldm_LowGrayLevelEmphasis

393 lbp-3D-m1_gldm_SmallDependenceEmphasis

394 lbp-3D-m1_gldm_SmallDependenceHighGrayLevelEmphasis

395 lbp-3D-m1_gldm_SmallDependenceLowGrayLevelEmphasis

396 lbp-3D-m1_glszm_GrayLevelNonUniformity

397 lbp-3D-m1_glszm_GrayLevelNonUniformityNormalized

398 lbp-3D-m1_glszm_GrayLevelVariance

399 lbp-3D-m1_glszm_HighGrayLevelZoneEmphasis

400 lbp-3D-m1_glszm_LargeAreaEmphasis

401 lbp-3D-m1_glszm_LargeAreaHighGrayLevelEmphasis

402 lbp-3D-m1_glszm_LargeAreaLowGrayLevelEmphasis

403 lbp-3D-m1_glszm_LowGrayLevelZoneEmphasis

404 lbp-3D-m1_glszm_SizeZoneNonUniformity

405 lbp-3D-m1_glszm_SizeZoneNonUniformityNormalized

406 lbp-3D-m1_glszm_SmallAreaEmphasis

407 lbp-3D-m1_glszm_SmallAreaHighGrayLevelEmphasis

408 lbp-3D-m1_glszm_SmallAreaLowGrayLevelEmphasis

409 lbp-3D-m1_glszm_ZoneEntropy

410 lbp-3D-m1_glszm_ZonePercentage

411 lbp-3D-m1_glszm_ZoneVariance

412 lbp-3D-m1_glrlm_GrayLevelNonUniformity

413 lbp-3D-m1_glrlm_GrayLevelNonUniformityNormalized

414 lbp-3D-m1_glrlm_GrayLevelVariance

415 lbp-3D-m1_glrlm_HighGrayLevelRunEmphasis

416 lbp-3D-m1_glrlm_LongRunEmphasis

417 lbp-3D-m1_glrlm_LongRunHighGrayLevelEmphasis

418 lbp-3D-m1_glrlm_LongRunLowGrayLevelEmphasis

419 lbp-3D-m1_glrlm_LowGrayLevelRunEmphasis

420 lbp-3D-m1_glrlm_RunEntropy

421 lbp-3D-m1_glrlm_RunLengthNonUniformity

422 lbp-3D-m1_glrlm_RunLengthNonUniformityNormalized

423 lbp-3D-m1_glrlm_RunPercentage

424 lbp-3D-m1_glrlm_RunVariance

425 lbp-3D-m1_glrlm_ShortRunEmphasis

426 lbp-3D-m1_glrlm_ShortRunHighGrayLevelEmphasis

427 lbp-3D-m1_glrlm_ShortRunLowGrayLevelEmphasis

428 lbp-3D-m1_ngtdm_Busyness

429 lbp-3D-m1_ngtdm_Coarseness

430 lbp-3D-m1_ngtdm_Complexity

431 lbp-3D-m1_ngtdm_Contrast

432 lbp-3D-m1_ngtdm_Strength

433 lbp-3D-m2_firstorder_10Percentile

434 lbp-3D-m2_firstorder_90Percentile

435 lbp-3D-m2_firstorder_Energy

436 lbp-3D-m2_firstorder_Entropy

437 lbp-3D-m2_firstorder_InterquartileRange

438 lbp-3D-m2_firstorder_Kurtosis

439 lbp-3D-m2_firstorder_Maximum

440 lbp-3D-m2_firstorder_MeanAbsoluteDeviation

441 lbp-3D-m2_firstorder_Mean

442 lbp-3D-m2_firstorder_Median

443 lbp-3D-m2_firstorder_Minimum

444 lbp-3D-m2_firstorder_Range

445 lbp-3D-m2_firstorder_RobustMeanAbsoluteDeviation

446 lbp-3D-m2_firstorder_RootMeanSquared

447 lbp-3D-m2_firstorder_Skewness

448 lbp-3D-m2_firstorder_TotalEnergy

449 lbp-3D-m2_firstorder_Uniformity

450 lbp-3D-m2_firstorder_Variance

451 lbp-3D-m2_glcm_Autocorrelation

452 lbp-3D-m2_glcm_ClusterProminence

453 lbp-3D-m2_glcm_ClusterShade

454 lbp-3D-m2_glcm_ClusterTendency

455 lbp-3D-m2_glcm_Contrast

456 lbp-3D-m2_glcm_Correlation

457 lbp-3D-m2_glcm_DifferenceAverage

458 lbp-3D-m2_glcm_DifferenceEntropy

459 lbp-3D-m2_glcm_DifferenceVariance

460 lbp-3D-m2_glcm_Id

461 lbp-3D-m2_glcm_Idm

462 lbp-3D-m2_glcm_Idmn

463 lbp-3D-m2_glcm_Idn

464 lbp-3D-m2_glcm_Imc1

465 lbp-3D-m2_glcm_Imc2

466 lbp-3D-m2_glcm_InverseVariance

467 lbp-3D-m2_glcm_JointAverage

468 lbp-3D-m2_glcm_JointEnergy

469 lbp-3D-m2_glcm_JointEntropy

470 lbp-3D-m2_glcm_MCC

471 lbp-3D-m2_glcm_MaximumProbability

472 lbp-3D-m2_glcm_SumAverage

473 lbp-3D-m2_glcm_SumEntropy

474 lbp-3D-m2_glcm_SumSquares

475 lbp-3D-m2_gldm_DependenceEntropy

476 lbp-3D-m2_gldm_DependenceNonUniformity

477 lbp-3D-m2_gldm_DependenceNonUniformityNormalized

478 lbp-3D-m2_gldm_DependenceVariance

479 lbp-3D-m2_gldm_GrayLevelNonUniformity

480 lbp-3D-m2_gldm_GrayLevelVariance

481 lbp-3D-m2_gldm_HighGrayLevelEmphasis

482 lbp-3D-m2_gldm_LargeDependenceEmphasis

483 lbp-3D-m2_gldm_LargeDependenceHighGrayLevelEmphasis

484 lbp-3D-m2_gldm_LargeDependenceLowGrayLevelEmphasis

485 lbp-3D-m2_gldm_LowGrayLevelEmphasis

486 lbp-3D-m2_gldm_SmallDependenceEmphasis

487 lbp-3D-m2_gldm_SmallDependenceHighGrayLevelEmphasis

488 lbp-3D-m2_gldm_SmallDependenceLowGrayLevelEmphasis

489 lbp-3D-m2_glszm_GrayLevelNonUniformity

490 lbp-3D-m2_glszm_GrayLevelNonUniformityNormalized

491 lbp-3D-m2_glszm_GrayLevelVariance

492 lbp-3D-m2_glszm_HighGrayLevelZoneEmphasis

493 lbp-3D-m2_glszm_LargeAreaEmphasis

494 lbp-3D-m2_glszm_LargeAreaHighGrayLevelEmphasis

495 lbp-3D-m2_glszm_LargeAreaLowGrayLevelEmphasis

496 lbp-3D-m2_glszm_LowGrayLevelZoneEmphasis

497 lbp-3D-m2_glszm_SizeZoneNonUniformity

498 lbp-3D-m2_glszm_SizeZoneNonUniformityNormalized

499 lbp-3D-m2_glszm_SmallAreaEmphasis

500 lbp-3D-m2_glszm_SmallAreaHighGrayLevelEmphasis

501 lbp-3D-m2_glszm_SmallAreaLowGrayLevelEmphasis

502 lbp-3D-m2_glszm_ZoneEntropy

503 lbp-3D-m2_glszm_ZonePercentage

504 lbp-3D-m2_glszm_ZoneVariance

505 lbp-3D-m2_glrlm_GrayLevelNonUniformity

506 lbp-3D-m2_glrlm_GrayLevelNonUniformityNormalized

507 lbp-3D-m2_glrlm_GrayLevelVariance

508 lbp-3D-m2_glrlm_HighGrayLevelRunEmphasis

509 lbp-3D-m2_glrlm_LongRunEmphasis

510 lbp-3D-m2_glrlm_LongRunHighGrayLevelEmphasis

511 lbp-3D-m2_glrlm_LongRunLowGrayLevelEmphasis

512 lbp-3D-m2_glrlm_LowGrayLevelRunEmphasis

513 lbp-3D-m2_glrlm_RunEntropy

514 lbp-3D-m2_glrlm_RunLengthNonUniformity

515 lbp-3D-m2_glrlm_RunLengthNonUniformityNormalized

516 lbp-3D-m2_glrlm_RunPercentage

517 lbp-3D-m2_glrlm_RunVariance

518 lbp-3D-m2_glrlm_ShortRunEmphasis

519 lbp-3D-m2_glrlm_ShortRunHighGrayLevelEmphasis

520 lbp-3D-m2_glrlm_ShortRunLowGrayLevelEmphasis

521 lbp-3D-m2_ngtdm_Busyness

522 lbp-3D-m2_ngtdm_Coarseness

523 lbp-3D-m2_ngtdm_Complexity

524 lbp-3D-m2_ngtdm_Contrast

525 lbp-3D-m2_ngtdm_Strength

526 lbp-3D-k_firstorder_10Percentile

527 lbp-3D-k_firstorder_90Percentile

528 lbp-3D-k_firstorder_Energy

529 lbp-3D-k_firstorder_Entropy

530 lbp-3D-k_firstorder_InterquartileRange

531 lbp-3D-k_firstorder_Kurtosis

532 lbp-3D-k_firstorder_Maximum

533 lbp-3D-k_firstorder_MeanAbsoluteDeviation

534 lbp-3D-k_firstorder_Mean

535 lbp-3D-k_firstorder_Median

536 lbp-3D-k_firstorder_Minimum

537 lbp-3D-k_firstorder_Range

538 lbp-3D-k_firstorder_RobustMeanAbsoluteDeviation

539 lbp-3D-k_firstorder_RootMeanSquared

540 lbp-3D-k_firstorder_Skewness

541 lbp-3D-k_firstorder_TotalEnergy

542 lbp-3D-k_firstorder_Uniformity

543 lbp-3D-k_firstorder_Variance

544 lbp-3D-k_glcm_Autocorrelation

545 lbp-3D-k_glcm_ClusterProminence

546 lbp-3D-k_glcm_ClusterShade

547 lbp-3D-k_glcm_ClusterTendency

548 lbp-3D-k_glcm_Contrast

549 lbp-3D-k_glcm_DifferenceAverage

550 lbp-3D-k_glcm_DifferenceEntropy

551 lbp-3D-k_glcm_DifferenceVariance

552 lbp-3D-k_glcm_Id

553 lbp-3D-k_glcm_Idm

554 lbp-3D-k_glcm_Idmn

555 lbp-3D-k_glcm_Idn

556 lbp-3D-k_glcm_Imc1

557 lbp-3D-k_glcm_Imc2

558 lbp-3D-k_glcm_InverseVariance

559 lbp-3D-k_glcm_JointAverage

560 lbp-3D-k_glcm_JointEnergy

561 lbp-3D-k_glcm_JointEntropy

562 lbp-3D-k_glcm_MaximumProbability

563 lbp-3D-k_glcm_SumAverage

564 lbp-3D-k_glcm_SumEntropy

565 lbp-3D-k_glcm_SumSquares

566 lbp-3D-k_gldm_DependenceEntropy

567 lbp-3D-k_gldm_DependenceNonUniformity

568 lbp-3D-k_gldm_DependenceNonUniformityNormalized

569 lbp-3D-k_gldm_DependenceVariance

570 lbp-3D-k_gldm_GrayLevelNonUniformity

571 lbp-3D-k_gldm_GrayLevelVariance

572 lbp-3D-k_gldm_HighGrayLevelEmphasis

573 lbp-3D-k_gldm_LargeDependenceEmphasis

574 lbp-3D-k_gldm_LargeDependenceHighGrayLevelEmphasis

575 lbp-3D-k_gldm_LargeDependenceLowGrayLevelEmphasis

576 lbp-3D-k_gldm_LowGrayLevelEmphasis

577 lbp-3D-k_gldm_SmallDependenceEmphasis

578 lbp-3D-k_gldm_SmallDependenceHighGrayLevelEmphasis

579 lbp-3D-k_gldm_SmallDependenceLowGrayLevelEmphasis

580 lbp-3D-k_glszm_GrayLevelNonUniformity

581 lbp-3D-k_glszm_HighGrayLevelZoneEmphasis

582 lbp-3D-k_glszm_LargeAreaEmphasis

583 lbp-3D-k_glszm_LargeAreaHighGrayLevelEmphasis

584 lbp-3D-k_glszm_LargeAreaLowGrayLevelEmphasis

585 lbp-3D-k_glszm_LowGrayLevelZoneEmphasis

586 lbp-3D-k_glszm_SizeZoneNonUniformity

587 lbp-3D-k_glszm_SizeZoneNonUniformityNormalized

588 lbp-3D-k_glszm_ZoneEntropy

589 lbp-3D-k_glszm_ZonePercentage

590 lbp-3D-k_glszm_ZoneVariance

591 lbp-3D-k_glrlm_GrayLevelNonUniformity

592 lbp-3D-k_glrlm_GrayLevelNonUniformityNormalized

593 lbp-3D-k_glrlm_GrayLevelVariance

594 lbp-3D-k_glrlm_HighGrayLevelRunEmphasis

595 lbp-3D-k_glrlm_LongRunEmphasis

596 lbp-3D-k_glrlm_LongRunHighGrayLevelEmphasis

597 lbp-3D-k_glrlm_LongRunLowGrayLevelEmphasis

598 lbp-3D-k_glrlm_LowGrayLevelRunEmphasis

599 lbp-3D-k_glrlm_RunEntropy

600 lbp-3D-k_glrlm_RunLengthNonUniformity

601 lbp-3D-k_glrlm_RunLengthNonUniformityNormalized

602 lbp-3D-k_glrlm_RunPercentage

603 lbp-3D-k_glrlm_RunVariance

604 lbp-3D-k_glrlm_ShortRunEmphasis

605 lbp-3D-k_glrlm_ShortRunHighGrayLevelEmphasis

606 lbp-3D-k_glrlm_ShortRunLowGrayLevelEmphasis

607 lbp-3D-k_ngtdm_Busyness

608 lbp-3D-k_ngtdm_Complexity

609 lbp-3D-k_ngtdm_Contrast

610 logarithm_firstorder_10Percentile

611 logarithm_firstorder_90Percentile

612 logarithm_firstorder_Energy

613 logarithm_firstorder_Entropy

614 logarithm_firstorder_InterquartileRange

615 logarithm_firstorder_Kurtosis

616 logarithm_firstorder_Maximum

617 logarithm_firstorder_MeanAbsoluteDeviation

618 logarithm_firstorder_Mean

619 logarithm_firstorder_Median

620 logarithm_firstorder_Minimum

621 logarithm_firstorder_Range

622 logarithm_firstorder_RobustMeanAbsoluteDeviation

623 logarithm_firstorder_RootMeanSquared

624 logarithm_firstorder_Skewness

625 logarithm_firstorder_TotalEnergy

626 logarithm_firstorder_Uniformity

627 logarithm_firstorder_Variance

628 logarithm_glcm_ClusterProminence

629 logarithm_glcm_ClusterShade

630 logarithm_glcm_ClusterTendency

631 logarithm_glcm_Contrast

632 logarithm_glcm_DifferenceAverage

633 logarithm_glcm_DifferenceEntropy

634 logarithm_glcm_DifferenceVariance

635 logarithm_glcm_Id

636 logarithm_glcm_Idm

637 logarithm_glcm_Idmn

638 logarithm_glcm_Idn

639 logarithm_glcm_Imc1

640 logarithm_glcm_Imc2

641 logarithm_glcm_InverseVariance

642 logarithm_glcm_JointEnergy

643 logarithm_glcm_JointEntropy

644 logarithm_glcm_MaximumProbability

645 logarithm_glcm_SumEntropy

646 logarithm_glcm_SumSquares

647 logarithm_gldm_DependenceEntropy

648 logarithm_gldm_DependenceNonUniformity

649 logarithm_gldm_DependenceNonUniformityNormalized

650 logarithm_gldm_DependenceVariance

651 logarithm_gldm_GrayLevelNonUniformity

652 logarithm_gldm_GrayLevelVariance

653 logarithm_gldm_LargeDependenceEmphasis

654 logarithm_gldm_SmallDependenceEmphasis

655 logarithm_glszm_GrayLevelNonUniformity

656 logarithm_glszm_HighGrayLevelZoneEmphasis

657 logarithm_glszm_LargeAreaEmphasis

658 logarithm_glszm_LargeAreaHighGrayLevelEmphasis

659 logarithm_glszm_ZoneEntropy

660 logarithm_glrlm_GrayLevelNonUniformity

661 logarithm_glrlm_GrayLevelNonUniformityNormalized

662 logarithm_glrlm_GrayLevelVariance

663 logarithm_glrlm_LongRunEmphasis

664 logarithm_glrlm_RunEntropy

665 logarithm_glrlm_RunLengthNonUniformity

666 logarithm_glrlm_RunLengthNonUniformityNormalized

667 logarithm_glrlm_RunPercentage

668 logarithm_glrlm_RunVariance

669 logarithm_glrlm_ShortRunEmphasis

670 logarithm_ngtdm_Busyness

671 logarithm_ngtdm_Complexity

672 logarithm_ngtdm_Contrast

673 square_firstorder_10Percentile

674 square_firstorder_90Percentile

675 square_firstorder_Energy

676 square_firstorder_Entropy

677 square_firstorder_InterquartileRange

678 square_firstorder_Kurtosis

679 square_firstorder_Maximum

680 square_firstorder_MeanAbsoluteDeviation

681 square_firstorder_Mean

682 square_firstorder_Median

683 square_firstorder_Range

684 square_firstorder_RobustMeanAbsoluteDeviation

685 square_firstorder_RootMeanSquared

686 square_firstorder_Skewness

687 square_firstorder_TotalEnergy

688 square_firstorder_Uniformity

689 square_firstorder_Variance

690 square_glcm_Autocorrelation

691 square_glcm_ClusterProminence

692 square_glcm_ClusterShade

693 square_glcm_ClusterTendency

694 square_glcm_Contrast

695 square_glcm_Correlation

696 square_glcm_DifferenceAverage

697 square_glcm_DifferenceEntropy

698 square_glcm_DifferenceVariance

699 square_glcm_Id

700 square_glcm_Idm

701 square_glcm_Idmn

702 square_glcm_Idn

703 square_glcm_Imc1

704 square_glcm_Imc2

705 square_glcm_InverseVariance

706 square_glcm_JointAverage

707 square_glcm_JointEnergy

708 square_glcm_JointEntropy

709 square_glcm_MCC

710 square_glcm_MaximumProbability

711 square_glcm_SumAverage

712 square_glcm_SumEntropy

713 square_glcm_SumSquares

714 square_gldm_DependenceEntropy

715 square_gldm_DependenceNonUniformity

716 square_gldm_DependenceNonUniformityNormalized

717 square_gldm_DependenceVariance

718 square_gldm_GrayLevelNonUniformity

719 square_gldm_GrayLevelVariance

720 square_gldm_HighGrayLevelEmphasis

721 square_gldm_LargeDependenceEmphasis

722 square_gldm_LargeDependenceHighGrayLevelEmphasis

723 square_gldm_LargeDependenceLowGrayLevelEmphasis

724 square_gldm_LowGrayLevelEmphasis

725 square_gldm_SmallDependenceEmphasis

726 square_gldm_SmallDependenceHighGrayLevelEmphasis

727 square_gldm_SmallDependenceLowGrayLevelEmphasis

728 square_glszm_GrayLevelNonUniformity

729 square_glszm_GrayLevelNonUniformityNormalized

730 square_glszm_GrayLevelVariance

731 square_glszm_HighGrayLevelZoneEmphasis

732 square_glszm_LargeAreaEmphasis

733 square_glszm_LargeAreaHighGrayLevelEmphasis

734 square_glszm_LargeAreaLowGrayLevelEmphasis

735 square_glszm_LowGrayLevelZoneEmphasis

736 square_glszm_SizeZoneNonUniformity

737 square_glszm_SizeZoneNonUniformityNormalized

738 square_glszm_SmallAreaHighGrayLevelEmphasis

739 square_glszm_ZoneEntropy

740 square_glszm_ZonePercentage

741 square_glrlm_GrayLevelNonUniformity

742 square_glrlm_GrayLevelNonUniformityNormalized

743 square_glrlm_GrayLevelVariance

744 square_glrlm_HighGrayLevelRunEmphasis

745 square_glrlm_LongRunEmphasis

746 square_glrlm_LongRunHighGrayLevelEmphasis

747 square_glrlm_LongRunLowGrayLevelEmphasis

748 square_glrlm_LowGrayLevelRunEmphasis

749 square_glrlm_RunEntropy

750 square_glrlm_RunLengthNonUniformity

751 square_glrlm_RunLengthNonUniformityNormalized

752 square_glrlm_RunPercentage

753 square_glrlm_RunVariance

754 square_glrlm_ShortRunEmphasis

755 square_glrlm_ShortRunHighGrayLevelEmphasis

756 square_glrlm_ShortRunLowGrayLevelEmphasis

757 square_ngtdm_Busyness

758 square_ngtdm_Coarseness

759 square_ngtdm_Complexity

760 square_ngtdm_Contrast

761 square_ngtdm_Strength

762 squareroot_firstorder_10Percentile

763 squareroot_firstorder_90Percentile

764 squareroot_firstorder_Energy

765 squareroot_firstorder_Entropy

766 squareroot_firstorder_InterquartileRange

767 squareroot_firstorder_Kurtosis

768 squareroot_firstorder_Maximum

769 squareroot_firstorder_MeanAbsoluteDeviation

770 squareroot_firstorder_Mean

771 squareroot_firstorder_Median

772 squareroot_firstorder_Range

773 squareroot_firstorder_RobustMeanAbsoluteDeviation

774 squareroot_firstorder_RootMeanSquared

775 squareroot_firstorder_Skewness

776 squareroot_firstorder_TotalEnergy

777 squareroot_firstorder_Uniformity

778 squareroot_firstorder_Variance

779 squareroot_glcm_ClusterProminence

780 squareroot_glcm_ClusterShade

781 squareroot_glcm_ClusterTendency

782 squareroot_glcm_Contrast

783 squareroot_glcm_DifferenceAverage

784 squareroot_glcm_DifferenceEntropy

785 squareroot_glcm_DifferenceVariance

786 squareroot_glcm_Id

787 squareroot_glcm_Idm

788 squareroot_glcm_Idmn

789 squareroot_glcm_Idn

790 squareroot_glcm_Imc1

791 squareroot_glcm_Imc2

792 squareroot_glcm_InverseVariance

793 squareroot_glcm_JointEnergy

794 squareroot_glcm_JointEntropy

795 squareroot_glcm_MaximumProbability

796 squareroot_glcm_SumEntropy

797 squareroot_glcm_SumSquares

798 squareroot_gldm_DependenceEntropy

799 squareroot_gldm_DependenceNonUniformity

800 squareroot_gldm_DependenceNonUniformityNormalized

801 squareroot_gldm_DependenceVariance

802 squareroot_gldm_GrayLevelNonUniformity

803 squareroot_gldm_GrayLevelVariance

804 squareroot_gldm_LargeDependenceEmphasis

805 squareroot_gldm_SmallDependenceEmphasis

806 squareroot_glszm_GrayLevelNonUniformity

807 squareroot_glszm_HighGrayLevelZoneEmphasis

808 squareroot_glszm_LargeAreaEmphasis

809 squareroot_glszm_LargeAreaHighGrayLevelEmphasis

810 squareroot_glszm_ZoneEntropy

811 squareroot_glrlm_GrayLevelNonUniformity

812 squareroot_glrlm_GrayLevelNonUniformityNormalized

813 squareroot_glrlm_GrayLevelVariance

814 squareroot_glrlm_LongRunEmphasis

815 squareroot_glrlm_RunEntropy

816 squareroot_glrlm_RunLengthNonUniformity

817 squareroot_glrlm_RunLengthNonUniformityNormalized

818 squareroot_glrlm_RunPercentage

819 squareroot_glrlm_RunVariance

820 squareroot_glrlm_ShortRunEmphasis

821 squareroot_ngtdm_Busyness

822 squareroot_ngtdm_Complexity

823 squareroot_ngtdm_Contrast

824 wavelet-LLH_firstorder_10Percentile

825 wavelet-LLH_firstorder_90Percentile

826 wavelet-LLH_firstorder_Energy

827 wavelet-LLH_firstorder_Entropy

828 wavelet-LLH_firstorder_InterquartileRange

829 wavelet-LLH_firstorder_Kurtosis

830 wavelet-LLH_firstorder_Maximum

831 wavelet-LLH_firstorder_MeanAbsoluteDeviation

832 wavelet-LLH_firstorder_Mean

833 wavelet-LLH_firstorder_Median

834 wavelet-LLH_firstorder_Minimum

835 wavelet-LLH_firstorder_Range

836 wavelet-LLH_firstorder_RobustMeanAbsoluteDeviation

837 wavelet-LLH_firstorder_RootMeanSquared

838 wavelet-LLH_firstorder_Skewness

839 wavelet-LLH_firstorder_TotalEnergy

840 wavelet-LLH_firstorder_Uniformity

841 wavelet-LLH_firstorder_Variance

842 wavelet-LLH_glcm_Autocorrelation

843 wavelet-LLH_glcm_ClusterProminence

844 wavelet-LLH_glcm_ClusterShade

845 wavelet-LLH_glcm_ClusterTendency

846 wavelet-LLH_glcm_Contrast

847 wavelet-LLH_glcm_Correlation

848 wavelet-LLH_glcm_DifferenceAverage

849 wavelet-LLH_glcm_DifferenceEntropy

850 wavelet-LLH_glcm_DifferenceVariance

851 wavelet-LLH_glcm_Id

852 wavelet-LLH_glcm_Idm

853 wavelet-LLH_glcm_Idmn

854 wavelet-LLH_glcm_Idn

855 wavelet-LLH_glcm_Imc1

856 wavelet-LLH_glcm_Imc2

857 wavelet-LLH_glcm_InverseVariance

858 wavelet-LLH_glcm_JointAverage

859 wavelet-LLH_glcm_JointEnergy

860 wavelet-LLH_glcm_JointEntropy

861 wavelet-LLH_glcm_MCC

862 wavelet-LLH_glcm_MaximumProbability

863 wavelet-LLH_glcm_SumAverage

864 wavelet-LLH_glcm_SumEntropy

865 wavelet-LLH_glcm_SumSquares

866 wavelet-LLH_gldm_DependenceEntropy

867 wavelet-LLH_gldm_DependenceNonUniformity

868 wavelet-LLH_gldm_DependenceNonUniformityNormalized

869 wavelet-LLH_gldm_DependenceVariance

870 wavelet-LLH_gldm_GrayLevelNonUniformity

871 wavelet-LLH_gldm_GrayLevelVariance

872 wavelet-LLH_gldm_HighGrayLevelEmphasis

873 wavelet-LLH_gldm_LargeDependenceEmphasis

874 wavelet-LLH_gldm_LargeDependenceHighGrayLevelEmphasis

875 wavelet-LLH_gldm_LargeDependenceLowGrayLevelEmphasis

876 wavelet-LLH_gldm_LowGrayLevelEmphasis

877 wavelet-LLH_gldm_SmallDependenceEmphasis

878 wavelet-LLH_gldm_SmallDependenceHighGrayLevelEmphasis

879 wavelet-LLH_gldm_SmallDependenceLowGrayLevelEmphasis

880 wavelet-LLH_glszm_GrayLevelNonUniformity

881 wavelet-LLH_glszm_GrayLevelNonUniformityNormalized

882 wavelet-LLH_glszm_GrayLevelVariance

883 wavelet-LLH_glszm_HighGrayLevelZoneEmphasis

884 wavelet-LLH_glszm_LargeAreaEmphasis

885 wavelet-LLH_glszm_LargeAreaHighGrayLevelEmphasis

886 wavelet-LLH_glszm_LargeAreaLowGrayLevelEmphasis

887 wavelet-LLH_glszm_LowGrayLevelZoneEmphasis

888 wavelet-LLH_glszm_SizeZoneNonUniformityNormalized

889 wavelet-LLH_glszm_SmallAreaHighGrayLevelEmphasis

890 wavelet-LLH_glszm_ZoneEntropy

891 wavelet-LLH_glszm_ZonePercentage

892 wavelet-LLH_glszm_ZoneVariance

893 wavelet-LLH_glrlm_GrayLevelNonUniformity

894 wavelet-LLH_glrlm_GrayLevelNonUniformityNormalized

895 wavelet-LLH_glrlm_GrayLevelVariance

896 wavelet-LLH_glrlm_HighGrayLevelRunEmphasis

897 wavelet-LLH_glrlm_LongRunEmphasis

898 wavelet-LLH_glrlm_LongRunHighGrayLevelEmphasis

899 wavelet-LLH_glrlm_LongRunLowGrayLevelEmphasis

900 wavelet-LLH_glrlm_LowGrayLevelRunEmphasis

901 wavelet-LLH_glrlm_RunEntropy

902 wavelet-LLH_glrlm_RunLengthNonUniformity

903 wavelet-LLH_glrlm_RunLengthNonUniformityNormalized

904 wavelet-LLH_glrlm_RunPercentage

905 wavelet-LLH_glrlm_RunVariance

906 wavelet-LLH_glrlm_ShortRunEmphasis

907 wavelet-LLH_glrlm_ShortRunHighGrayLevelEmphasis

908 wavelet-LLH_glrlm_ShortRunLowGrayLevelEmphasis

909 wavelet-LLH_ngtdm_Busyness

910 wavelet-LLH_ngtdm_Coarseness

911 wavelet-LLH_ngtdm_Complexity

912 wavelet-LLH_ngtdm_Contrast

913 wavelet-LLH_ngtdm_Strength

914 wavelet-LHL_firstorder_10Percentile

915 wavelet-LHL_firstorder_90Percentile

916 wavelet-LHL_firstorder_Energy

917 wavelet-LHL_firstorder_Entropy

918 wavelet-LHL_firstorder_InterquartileRange

919 wavelet-LHL_firstorder_Kurtosis

920 wavelet-LHL_firstorder_Maximum

921 wavelet-LHL_firstorder_MeanAbsoluteDeviation

922 wavelet-LHL_firstorder_Mean

923 wavelet-LHL_firstorder_Median

924 wavelet-LHL_firstorder_Minimum

925 wavelet-LHL_firstorder_Range

926 wavelet-LHL_firstorder_RobustMeanAbsoluteDeviation

927 wavelet-LHL_firstorder_RootMeanSquared

928 wavelet-LHL_firstorder_Skewness

929 wavelet-LHL_firstorder_TotalEnergy

930 wavelet-LHL_firstorder_Uniformity

931 wavelet-LHL_firstorder_Variance

932 wavelet-LHL_glcm_Autocorrelation

933 wavelet-LHL_glcm_ClusterProminence

934 wavelet-LHL_glcm_ClusterShade

935 wavelet-LHL_glcm_ClusterTendency

936 wavelet-LHL_glcm_Contrast

937 wavelet-LHL_glcm_Correlation

938 wavelet-LHL_glcm_DifferenceAverage

939 wavelet-LHL_glcm_DifferenceEntropy

940 wavelet-LHL_glcm_DifferenceVariance

941 wavelet-LHL_glcm_Id

942 wavelet-LHL_glcm_Idm

943 wavelet-LHL_glcm_Idmn

944 wavelet-LHL_glcm_Idn

945 wavelet-LHL_glcm_Imc1

946 wavelet-LHL_glcm_Imc2

947 wavelet-LHL_glcm_InverseVariance

948 wavelet-LHL_glcm_JointAverage

949 wavelet-LHL_glcm_JointEnergy

950 wavelet-LHL_glcm_JointEntropy

951 wavelet-LHL_glcm_MCC

952 wavelet-LHL_glcm_MaximumProbability

953 wavelet-LHL_glcm_SumAverage

954 wavelet-LHL_glcm_SumEntropy

955 wavelet-LHL_glcm_SumSquares

956 wavelet-LHL_gldm_DependenceEntropy

957 wavelet-LHL_gldm_DependenceNonUniformity

958 wavelet-LHL_gldm_DependenceNonUniformityNormalized

959 wavelet-LHL_gldm_DependenceVariance

960 wavelet-LHL_gldm_GrayLevelNonUniformity

961 wavelet-LHL_gldm_GrayLevelVariance

962 wavelet-LHL_gldm_HighGrayLevelEmphasis

963 wavelet-LHL_gldm_LargeDependenceEmphasis

964 wavelet-LHL_gldm_LargeDependenceHighGrayLevelEmphasis

965 wavelet-LHL_gldm_LargeDependenceLowGrayLevelEmphasis

966 wavelet-LHL_gldm_LowGrayLevelEmphasis

967 wavelet-LHL_gldm_SmallDependenceEmphasis

968 wavelet-LHL_gldm_SmallDependenceHighGrayLevelEmphasis

969 wavelet-LHL_gldm_SmallDependenceLowGrayLevelEmphasis

970 wavelet-LHL_glszm_GrayLevelNonUniformity

971 wavelet-LHL_glszm_GrayLevelNonUniformityNormalized

972 wavelet-LHL_glszm_GrayLevelVariance

973 wavelet-LHL_glszm_LargeAreaEmphasis

974 wavelet-LHL_glszm_LargeAreaHighGrayLevelEmphasis

975 wavelet-LHL_glszm_LargeAreaLowGrayLevelEmphasis

976 wavelet-LHL_glszm_ZoneEntropy

977 wavelet-LHL_glszm_ZonePercentage

978 wavelet-LHL_glszm_ZoneVariance

979 wavelet-LHL_glrlm_GrayLevelNonUniformity

980 wavelet-LHL_glrlm_GrayLevelNonUniformityNormalized

981 wavelet-LHL_glrlm_GrayLevelVariance

982 wavelet-LHL_glrlm_HighGrayLevelRunEmphasis

983 wavelet-LHL_glrlm_LongRunEmphasis

984 wavelet-LHL_glrlm_LongRunHighGrayLevelEmphasis

985 wavelet-LHL_glrlm_LongRunLowGrayLevelEmphasis

986 wavelet-LHL_glrlm_LowGrayLevelRunEmphasis

987 wavelet-LHL_glrlm_RunEntropy

988 wavelet-LHL_glrlm_RunLengthNonUniformity

989 wavelet-LHL_glrlm_RunLengthNonUniformityNormalized

990 wavelet-LHL_glrlm_RunPercentage

991 wavelet-LHL_glrlm_RunVariance

992 wavelet-LHL_glrlm_ShortRunEmphasis

993 wavelet-LHL_glrlm_ShortRunHighGrayLevelEmphasis

994 wavelet-LHL_glrlm_ShortRunLowGrayLevelEmphasis

995 wavelet-LHL_ngtdm_Busyness

996 wavelet-LHL_ngtdm_Coarseness

997 wavelet-LHL_ngtdm_Complexity

998 wavelet-LHL_ngtdm_Contrast

999 wavelet-LHL_ngtdm_Strength

1000 wavelet-LHH_firstorder_10Percentile

1001 wavelet-LHH_firstorder_90Percentile

1002 wavelet-LHH_firstorder_Energy

1003 wavelet-LHH_firstorder_InterquartileRange

1004 wavelet-LHH_firstorder_Kurtosis

1005 wavelet-LHH_firstorder_Maximum

1006 wavelet-LHH_firstorder_MeanAbsoluteDeviation

1007 wavelet-LHH_firstorder_Mean

1008 wavelet-LHH_firstorder_Median

1009 wavelet-LHH_firstorder_Minimum

1010 wavelet-LHH_firstorder_Range

1011 wavelet-LHH_firstorder_RobustMeanAbsoluteDeviation

1012 wavelet-LHH_firstorder_RootMeanSquared

1013 wavelet-LHH_firstorder_Skewness

1014 wavelet-LHH_firstorder_TotalEnergy

1015 wavelet-LHH_firstorder_Variance

1016 wavelet-LHH_glcm_Autocorrelation

1017 wavelet-LHH_glcm_ClusterProminence

1018 wavelet-LHH_glcm_ClusterShade

1019 wavelet-LHH_glcm_ClusterTendency

1020 wavelet-LHH_glcm_Contrast

1021 wavelet-LHH_glcm_Correlation

1022 wavelet-LHH_glcm_DifferenceAverage

1023 wavelet-LHH_glcm_DifferenceEntropy

1024 wavelet-LHH_glcm_DifferenceVariance

1025 wavelet-LHH_glcm_Id

1026 wavelet-LHH_glcm_Idm

1027 wavelet-LHH_glcm_Idmn

1028 wavelet-LHH_glcm_Idn

1029 wavelet-LHH_glcm_Imc1

1030 wavelet-LHH_glcm_Imc2

1031 wavelet-LHH_glcm_InverseVariance

1032 wavelet-LHH_glcm_JointAverage

1033 wavelet-LHH_glcm_JointEnergy

1034 wavelet-LHH_glcm_JointEntropy

1035 wavelet-LHH_glcm_MCC

1036 wavelet-LHH_glcm_MaximumProbability

1037 wavelet-LHH_glcm_SumAverage

1038 wavelet-LHH_glcm_SumEntropy

1039 wavelet-LHH_glcm_SumSquares

1040 wavelet-LHH_gldm_DependenceEntropy

1041 wavelet-LHH_gldm_DependenceNonUniformity

1042 wavelet-LHH_gldm_DependenceNonUniformityNormalized

1043 wavelet-LHH_gldm_DependenceVariance

1044 wavelet-LHH_gldm_GrayLevelNonUniformity

1045 wavelet-LHH_gldm_HighGrayLevelEmphasis

1046 wavelet-LHH_gldm_LargeDependenceEmphasis

1047 wavelet-LHH_gldm_LargeDependenceHighGrayLevelEmphasis

1048 wavelet-LHH_gldm_LargeDependenceLowGrayLevelEmphasis

1049 wavelet-LHH_gldm_LowGrayLevelEmphasis

1050 wavelet-LHH_gldm_SmallDependenceEmphasis

1051 wavelet-LHH_gldm_SmallDependenceHighGrayLevelEmphasis

1052 wavelet-LHH_gldm_SmallDependenceLowGrayLevelEmphasis

1053 wavelet-LHH_glszm_GrayLevelNonUniformity

1054 wavelet-LHH_glszm_LargeAreaEmphasis

1055 wavelet-LHH_glszm_LargeAreaHighGrayLevelEmphasis

1056 wavelet-LHH_glszm_LargeAreaLowGrayLevelEmphasis

1057 wavelet-LHH_glszm_SizeZoneNonUniformityNormalized

1058 wavelet-LHH_glszm_ZoneEntropy

1059 wavelet-LHH_glszm_ZonePercentage

1060 wavelet-LHH_glszm_ZoneVariance

1061 wavelet-LHH_glrlm_GrayLevelNonUniformity

1062 wavelet-LHH_glrlm_GrayLevelNonUniformityNormalized

1063 wavelet-LHH_glrlm_GrayLevelVariance

1064 wavelet-LHH_glrlm_HighGrayLevelRunEmphasis

1065 wavelet-LHH_glrlm_LongRunEmphasis

1066 wavelet-LHH_glrlm_LongRunHighGrayLevelEmphasis

1067 wavelet-LHH_glrlm_LongRunLowGrayLevelEmphasis

1068 wavelet-LHH_glrlm_LowGrayLevelRunEmphasis

1069 wavelet-LHH_glrlm_RunEntropy

1070 wavelet-LHH_glrlm_RunLengthNonUniformity

1071 wavelet-LHH_glrlm_RunLengthNonUniformityNormalized

1072 wavelet-LHH_glrlm_RunPercentage

1073 wavelet-LHH_glrlm_RunVariance

1074 wavelet-LHH_glrlm_ShortRunEmphasis

1075 wavelet-LHH_glrlm_ShortRunHighGrayLevelEmphasis

1076 wavelet-LHH_glrlm_ShortRunLowGrayLevelEmphasis

1077 wavelet-LHH_ngtdm_Busyness

1078 wavelet-LHH_ngtdm_Coarseness

1079 wavelet-LHH_ngtdm_Complexity

1080 wavelet-LHH_ngtdm_Contrast

1081 wavelet-LHH_ngtdm_Strength

1082 wavelet-HLL_firstorder_10Percentile

1083 wavelet-HLL_firstorder_90Percentile

1084 wavelet-HLL_firstorder_Energy

1085 wavelet-HLL_firstorder_Entropy

1086 wavelet-HLL_firstorder_InterquartileRange

1087 wavelet-HLL_firstorder_Kurtosis

1088 wavelet-HLL_firstorder_Maximum

1089 wavelet-HLL_firstorder_MeanAbsoluteDeviation

1090 wavelet-HLL_firstorder_Mean

1091 wavelet-HLL_firstorder_Median

1092 wavelet-HLL_firstorder_Minimum

1093 wavelet-HLL_firstorder_Range

1094 wavelet-HLL_firstorder_RobustMeanAbsoluteDeviation

1095 wavelet-HLL_firstorder_RootMeanSquared

1096 wavelet-HLL_firstorder_Skewness

1097 wavelet-HLL_firstorder_TotalEnergy

1098 wavelet-HLL_firstorder_Uniformity

1099 wavelet-HLL_firstorder_Variance

1100 wavelet-HLL_glcm_Autocorrelation

1101 wavelet-HLL_glcm_ClusterProminence

1102 wavelet-HLL_glcm_ClusterShade

1103 wavelet-HLL_glcm_ClusterTendency

1104 wavelet-HLL_glcm_Contrast

1105 wavelet-HLL_glcm_Correlation

1106 wavelet-HLL_glcm_DifferenceAverage

1107 wavelet-HLL_glcm_DifferenceEntropy

1108 wavelet-HLL_glcm_DifferenceVariance

1109 wavelet-HLL_glcm_Id

1110 wavelet-HLL_glcm_Idm

1111 wavelet-HLL_glcm_Idmn

1112 wavelet-HLL_glcm_Idn

1113 wavelet-HLL_glcm_Imc1

1114 wavelet-HLL_glcm_Imc2

1115 wavelet-HLL_glcm_InverseVariance

1116 wavelet-HLL_glcm_JointAverage

1117 wavelet-HLL_glcm_JointEnergy

1118 wavelet-HLL_glcm_JointEntropy

1119 wavelet-HLL_glcm_MCC

1120 wavelet-HLL_glcm_MaximumProbability

1121 wavelet-HLL_glcm_SumAverage

1122 wavelet-HLL_glcm_SumEntropy

1123 wavelet-HLL_glcm_SumSquares

1124 wavelet-HLL_gldm_DependenceEntropy

1125 wavelet-HLL_gldm_DependenceNonUniformity

1126 wavelet-HLL_gldm_DependenceNonUniformityNormalized

1127 wavelet-HLL_gldm_DependenceVariance

1128 wavelet-HLL_gldm_GrayLevelNonUniformity

1129 wavelet-HLL_gldm_GrayLevelVariance

1130 wavelet-HLL_gldm_HighGrayLevelEmphasis

1131 wavelet-HLL_gldm_LargeDependenceEmphasis

1132 wavelet-HLL_gldm_LargeDependenceHighGrayLevelEmphasis

1133 wavelet-HLL_gldm_LargeDependenceLowGrayLevelEmphasis

1134 wavelet-HLL_gldm_LowGrayLevelEmphasis

1135 wavelet-HLL_gldm_SmallDependenceEmphasis

1136 wavelet-HLL_gldm_SmallDependenceLowGrayLevelEmphasis

1137 wavelet-HLL_glszm_GrayLevelNonUniformity

1138 wavelet-HLL_glszm_LargeAreaEmphasis

1139 wavelet-HLL_glszm_LargeAreaHighGrayLevelEmphasis

1140 wavelet-HLL_glszm_LargeAreaLowGrayLevelEmphasis

1141 wavelet-HLL_glszm_ZoneEntropy

1142 wavelet-HLL_glszm_ZoneVariance

1143 wavelet-HLL_glrlm_GrayLevelNonUniformity

1144 wavelet-HLL_glrlm_GrayLevelNonUniformityNormalized

1145 wavelet-HLL_glrlm_GrayLevelVariance

1146 wavelet-HLL_glrlm_HighGrayLevelRunEmphasis

1147 wavelet-HLL_glrlm_LongRunEmphasis

1148 wavelet-HLL_glrlm_LongRunHighGrayLevelEmphasis

1149 wavelet-HLL_glrlm_LongRunLowGrayLevelEmphasis

1150 wavelet-HLL_glrlm_LowGrayLevelRunEmphasis

1151 wavelet-HLL_glrlm_RunEntropy

1152 wavelet-HLL_glrlm_RunLengthNonUniformity

1153 wavelet-HLL_glrlm_RunLengthNonUniformityNormalized

1154 wavelet-HLL_glrlm_RunPercentage

1155 wavelet-HLL_glrlm_RunVariance

1156 wavelet-HLL_glrlm_ShortRunEmphasis

1157 wavelet-HLL_glrlm_ShortRunHighGrayLevelEmphasis

1158 wavelet-HLL_glrlm_ShortRunLowGrayLevelEmphasis

1159 wavelet-HLL_ngtdm_Busyness

1160 wavelet-HLL_ngtdm_Coarseness

1161 wavelet-HLL_ngtdm_Complexity

1162 wavelet-HLL_ngtdm_Contrast

1163 wavelet-HLL_ngtdm_Strength

1164 wavelet-HLH_firstorder_10Percentile

1165 wavelet-HLH_firstorder_90Percentile

1166 wavelet-HLH_firstorder_Energy

1167 wavelet-HLH_firstorder_Entropy

1168 wavelet-HLH_firstorder_InterquartileRange

1169 wavelet-HLH_firstorder_Kurtosis

1170 wavelet-HLH_firstorder_Maximum

1171 wavelet-HLH_firstorder_MeanAbsoluteDeviation

1172 wavelet-HLH_firstorder_Mean

1173 wavelet-HLH_firstorder_Median

1174 wavelet-HLH_firstorder_Minimum

1175 wavelet-HLH_firstorder_Range

1176 wavelet-HLH_firstorder_RobustMeanAbsoluteDeviation

1177 wavelet-HLH_firstorder_RootMeanSquared

1178 wavelet-HLH_firstorder_Skewness

1179 wavelet-HLH_firstorder_TotalEnergy

1180 wavelet-HLH_firstorder_Uniformity

1181 wavelet-HLH_firstorder_Variance

1182 wavelet-HLH_glcm_Autocorrelation

1183 wavelet-HLH_glcm_ClusterProminence

1184 wavelet-HLH_glcm_ClusterShade

1185 wavelet-HLH_glcm_ClusterTendency

1186 wavelet-HLH_glcm_Contrast

1187 wavelet-HLH_glcm_Correlation

1188 wavelet-HLH_glcm_DifferenceAverage

1189 wavelet-HLH_glcm_DifferenceEntropy

1190 wavelet-HLH_glcm_DifferenceVariance

1191 wavelet-HLH_glcm_Id

1192 wavelet-HLH_glcm_Idm

1193 wavelet-HLH_glcm_Idmn

1194 wavelet-HLH_glcm_Idn

1195 wavelet-HLH_glcm_Imc1

1196 wavelet-HLH_glcm_Imc2

1197 wavelet-HLH_glcm_InverseVariance

1198 wavelet-HLH_glcm_JointAverage

1199 wavelet-HLH_glcm_JointEnergy

1200 wavelet-HLH_glcm_JointEntropy

1201 wavelet-HLH_glcm_MCC

1202 wavelet-HLH_glcm_MaximumProbability

1203 wavelet-HLH_glcm_SumAverage

1204 wavelet-HLH_glcm_SumEntropy

1205 wavelet-HLH_glcm_SumSquares

1206 wavelet-HLH_gldm_DependenceEntropy

1207 wavelet-HLH_gldm_DependenceNonUniformity

1208 wavelet-HLH_gldm_DependenceNonUniformityNormalized

1209 wavelet-HLH_gldm_DependenceVariance

1210 wavelet-HLH_gldm_GrayLevelNonUniformity

1211 wavelet-HLH_gldm_GrayLevelVariance

1212 wavelet-HLH_gldm_HighGrayLevelEmphasis

1213 wavelet-HLH_gldm_LargeDependenceEmphasis

1214 wavelet-HLH_gldm_LargeDependenceHighGrayLevelEmphasis

1215 wavelet-HLH_gldm_LargeDependenceLowGrayLevelEmphasis

1216 wavelet-HLH_gldm_LowGrayLevelEmphasis

1217 wavelet-HLH_gldm_SmallDependenceEmphasis

1218 wavelet-HLH_gldm_SmallDependenceHighGrayLevelEmphasis

1219 wavelet-HLH_gldm_SmallDependenceLowGrayLevelEmphasis

1220 wavelet-HLH_glszm_GrayLevelNonUniformity

1221 wavelet-HLH_glszm_LargeAreaEmphasis

1222 wavelet-HLH_glszm_LargeAreaHighGrayLevelEmphasis

1223 wavelet-HLH_glszm_LargeAreaLowGrayLevelEmphasis

1224 wavelet-HLH_glszm_ZoneEntropy

1225 wavelet-HLH_glszm_ZoneVariance

1226 wavelet-HLH_glrlm_GrayLevelNonUniformity

1227 wavelet-HLH_glrlm_GrayLevelNonUniformityNormalized

1228 wavelet-HLH_glrlm_GrayLevelVariance

1229 wavelet-HLH_glrlm_HighGrayLevelRunEmphasis

1230 wavelet-HLH_glrlm_LongRunEmphasis

1231 wavelet-HLH_glrlm_LongRunHighGrayLevelEmphasis

1232 wavelet-HLH_glrlm_LongRunLowGrayLevelEmphasis

1233 wavelet-HLH_glrlm_LowGrayLevelRunEmphasis

1234 wavelet-HLH_glrlm_RunEntropy

1235 wavelet-HLH_glrlm_RunLengthNonUniformity

1236 wavelet-HLH_glrlm_RunLengthNonUniformityNormalized

1237 wavelet-HLH_glrlm_RunPercentage

1238 wavelet-HLH_glrlm_RunVariance

1239 wavelet-HLH_glrlm_ShortRunEmphasis

1240 wavelet-HLH_glrlm_ShortRunHighGrayLevelEmphasis

1241 wavelet-HLH_glrlm_ShortRunLowGrayLevelEmphasis

1242 wavelet-HLH_ngtdm_Busyness

1243 wavelet-HLH_ngtdm_Coarseness

1244 wavelet-HLH_ngtdm_Complexity

1245 wavelet-HLH_ngtdm_Contrast

1246 wavelet-HLH_ngtdm_Strength

1247 wavelet-HHL_firstorder_10Percentile

1248 wavelet-HHL_firstorder_90Percentile

1249 wavelet-HHL_firstorder_Energy

1250 wavelet-HHL_firstorder_Entropy

1251 wavelet-HHL_firstorder_InterquartileRange

1252 wavelet-HHL_firstorder_Kurtosis

1253 wavelet-HHL_firstorder_Maximum

1254 wavelet-HHL_firstorder_MeanAbsoluteDeviation

1255 wavelet-HHL_firstorder_Median

1256 wavelet-HHL_firstorder_Minimum

1257 wavelet-HHL_firstorder_Range

1258 wavelet-HHL_firstorder_RobustMeanAbsoluteDeviation

1259 wavelet-HHL_firstorder_RootMeanSquared

1260 wavelet-HHL_firstorder_TotalEnergy

1261 wavelet-HHL_firstorder_Uniformity

1262 wavelet-HHL_firstorder_Variance

1263 wavelet-HHL_glcm_Autocorrelation

1264 wavelet-HHL_glcm_ClusterProminence

1265 wavelet-HHL_glcm_ClusterShade

1266 wavelet-HHL_glcm_ClusterTendency

1267 wavelet-HHL_glcm_Contrast

1268 wavelet-HHL_glcm_Correlation

1269 wavelet-HHL_glcm_DifferenceAverage

1270 wavelet-HHL_glcm_DifferenceEntropy

1271 wavelet-HHL_glcm_DifferenceVariance

1272 wavelet-HHL_glcm_Id

1273 wavelet-HHL_glcm_Idm

1274 wavelet-HHL_glcm_Idmn

1275 wavelet-HHL_glcm_Idn

1276 wavelet-HHL_glcm_Imc1

1277 wavelet-HHL_glcm_Imc2

1278 wavelet-HHL_glcm_InverseVariance

1279 wavelet-HHL_glcm_JointAverage

1280 wavelet-HHL_glcm_JointEnergy

1281 wavelet-HHL_glcm_JointEntropy

1282 wavelet-HHL_glcm_MCC

1283 wavelet-HHL_glcm_MaximumProbability

1284 wavelet-HHL_glcm_SumAverage

1285 wavelet-HHL_glcm_SumEntropy

1286 wavelet-HHL_glcm_SumSquares

1287 wavelet-HHL_gldm_DependenceEntropy

1288 wavelet-HHL_gldm_DependenceNonUniformity

1289 wavelet-HHL_gldm_DependenceNonUniformityNormalized

1290 wavelet-HHL_gldm_DependenceVariance

1291 wavelet-HHL_gldm_GrayLevelNonUniformity

1292 wavelet-HHL_gldm_GrayLevelVariance

1293 wavelet-HHL_gldm_HighGrayLevelEmphasis

1294 wavelet-HHL_gldm_LargeDependenceEmphasis

1295 wavelet-HHL_gldm_LargeDependenceHighGrayLevelEmphasis

1296 wavelet-HHL_gldm_LargeDependenceLowGrayLevelEmphasis

1297 wavelet-HHL_gldm_LowGrayLevelEmphasis

1298 wavelet-HHL_gldm_SmallDependenceEmphasis

1299 wavelet-HHL_gldm_SmallDependenceHighGrayLevelEmphasis

1300 wavelet-HHL_gldm_SmallDependenceLowGrayLevelEmphasis

1301 wavelet-HHL_glszm_GrayLevelNonUniformity

1302 wavelet-HHL_glszm_LargeAreaEmphasis

1303 wavelet-HHL_glszm_LargeAreaHighGrayLevelEmphasis

1304 wavelet-HHL_glszm_LargeAreaLowGrayLevelEmphasis

1305 wavelet-HHL_glszm_SizeZoneNonUniformity

1306 wavelet-HHL_glszm_SizeZoneNonUniformityNormalized

1307 wavelet-HHL_glszm_ZonePercentage

1308 wavelet-HHL_glszm_ZoneVariance

1309 wavelet-HHL_glrlm_GrayLevelNonUniformity

1310 wavelet-HHL_glrlm_GrayLevelNonUniformityNormalized

1311 wavelet-HHL_glrlm_GrayLevelVariance

1312 wavelet-HHL_glrlm_HighGrayLevelRunEmphasis

1313 wavelet-HHL_glrlm_LongRunEmphasis

1314 wavelet-HHL_glrlm_LongRunHighGrayLevelEmphasis

1315 wavelet-HHL_glrlm_LongRunLowGrayLevelEmphasis

1316 wavelet-HHL_glrlm_LowGrayLevelRunEmphasis

1317 wavelet-HHL_glrlm_RunEntropy

1318 wavelet-HHL_glrlm_RunLengthNonUniformity

1319 wavelet-HHL_glrlm_RunLengthNonUniformityNormalized

1320 wavelet-HHL_glrlm_RunPercentage

1321 wavelet-HHL_glrlm_RunVariance

1322 wavelet-HHL_glrlm_ShortRunEmphasis

1323 wavelet-HHL_glrlm_ShortRunHighGrayLevelEmphasis

1324 wavelet-HHL_glrlm_ShortRunLowGrayLevelEmphasis

1325 wavelet-HHL_ngtdm_Busyness

1326 wavelet-HHL_ngtdm_Coarseness

1327 wavelet-HHL_ngtdm_Complexity

1328 wavelet-HHL_ngtdm_Contrast

1329 wavelet-HHL_ngtdm_Strength

1330 wavelet-HHH_firstorder_10Percentile

1331 wavelet-HHH_firstorder_90Percentile

1332 wavelet-HHH_firstorder_Energy

1333 wavelet-HHH_firstorder_Entropy

1334 wavelet-HHH_firstorder_InterquartileRange

1335 wavelet-HHH_firstorder_Kurtosis

1336 wavelet-HHH_firstorder_Maximum

1337 wavelet-HHH_firstorder_MeanAbsoluteDeviation

1338 wavelet-HHH_firstorder_Median

1339 wavelet-HHH_firstorder_Minimum

1340 wavelet-HHH_firstorder_Range

1341 wavelet-HHH_firstorder_RobustMeanAbsoluteDeviation

1342 wavelet-HHH_firstorder_RootMeanSquared

1343 wavelet-HHH_firstorder_Skewness

1344 wavelet-HHH_firstorder_TotalEnergy

1345 wavelet-HHH_firstorder_Uniformity

1346 wavelet-HHH_firstorder_Variance

1347 wavelet-HHH_glcm_Autocorrelation

1348 wavelet-HHH_glcm_ClusterProminence

1349 wavelet-HHH_glcm_ClusterShade

1350 wavelet-HHH_glcm_ClusterTendency

1351 wavelet-HHH_glcm_Contrast

1352 wavelet-HHH_glcm_Correlation

1353 wavelet-HHH_glcm_DifferenceAverage

1354 wavelet-HHH_glcm_DifferenceEntropy

1355 wavelet-HHH_glcm_DifferenceVariance

1356 wavelet-HHH_glcm_Id

1357 wavelet-HHH_glcm_Idm

1358 wavelet-HHH_glcm_Idmn

1359 wavelet-HHH_glcm_Idn

1360 wavelet-HHH_glcm_Imc1

1361 wavelet-HHH_glcm_Imc2

1362 wavelet-HHH_glcm_InverseVariance

1363 wavelet-HHH_glcm_JointAverage

1364 wavelet-HHH_glcm_JointEnergy

1365 wavelet-HHH_glcm_JointEntropy

1366 wavelet-HHH_glcm_MCC

1367 wavelet-HHH_glcm_MaximumProbability

1368 wavelet-HHH_glcm_SumAverage

1369 wavelet-HHH_glcm_SumEntropy

1370 wavelet-HHH_glcm_SumSquares

1371 wavelet-HHH_gldm_DependenceEntropy

1372 wavelet-HHH_gldm_DependenceNonUniformity

1373 wavelet-HHH_gldm_DependenceNonUniformityNormalized

1374 wavelet-HHH_gldm_DependenceVariance

1375 wavelet-HHH_gldm_GrayLevelNonUniformity

1376 wavelet-HHH_gldm_GrayLevelVariance

1377 wavelet-HHH_gldm_HighGrayLevelEmphasis

1378 wavelet-HHH_gldm_LargeDependenceEmphasis

1379 wavelet-HHH_gldm_LargeDependenceHighGrayLevelEmphasis

1380 wavelet-HHH_gldm_LargeDependenceLowGrayLevelEmphasis

1381 wavelet-HHH_gldm_LowGrayLevelEmphasis

1382 wavelet-HHH_gldm_SmallDependenceEmphasis

1383 wavelet-HHH_gldm_SmallDependenceHighGrayLevelEmphasis

1384 wavelet-HHH_gldm_SmallDependenceLowGrayLevelEmphasis

1385 wavelet-HHH_glszm_GrayLevelNonUniformity

1386 wavelet-HHH_glszm_LargeAreaEmphasis

1387 wavelet-HHH_glszm_LargeAreaHighGrayLevelEmphasis

1388 wavelet-HHH_glszm_LargeAreaLowGrayLevelEmphasis

1389 wavelet-HHH_glszm_SizeZoneNonUniformity

1390 wavelet-HHH_glszm_ZoneVariance

1391 wavelet-HHH_glrlm_GrayLevelNonUniformity

1392 wavelet-HHH_glrlm_HighGrayLevelRunEmphasis

1393 wavelet-HHH_glrlm_LongRunEmphasis

1394 wavelet-HHH_glrlm_LongRunHighGrayLevelEmphasis

1395 wavelet-HHH_glrlm_LongRunLowGrayLevelEmphasis

1396 wavelet-HHH_glrlm_LowGrayLevelRunEmphasis

1397 wavelet-HHH_glrlm_RunEntropy

1398 wavelet-HHH_glrlm_RunLengthNonUniformity

1399 wavelet-HHH_glrlm_RunLengthNonUniformityNormalized

1400 wavelet-HHH_glrlm_RunPercentage

1401 wavelet-HHH_glrlm_RunVariance

1402 wavelet-HHH_glrlm_ShortRunEmphasis

1403 wavelet-HHH_glrlm_ShortRunHighGrayLevelEmphasis

1404 wavelet-HHH_glrlm_ShortRunLowGrayLevelEmphasis

1405 wavelet-HHH_ngtdm_Busyness

1406 wavelet-HHH_ngtdm_Coarseness

1407 wavelet-HHH_ngtdm_Complexity

1408 wavelet-HHH_ngtdm_Contrast

1409 wavelet-HHH_ngtdm_Strength

1410 wavelet-LLL_firstorder_10Percentile

1411 wavelet-LLL_firstorder_90Percentile

1412 wavelet-LLL_firstorder_Energy

1413 wavelet-LLL_firstorder_Entropy

1414 wavelet-LLL_firstorder_InterquartileRange

1415 wavelet-LLL_firstorder_Kurtosis

1416 wavelet-LLL_firstorder_Maximum

1417 wavelet-LLL_firstorder_MeanAbsoluteDeviation

1418 wavelet-LLL_firstorder_Mean

1419 wavelet-LLL_firstorder_Median

1420 wavelet-LLL_firstorder_Minimum

1421 wavelet-LLL_firstorder_Range

1422 wavelet-LLL_firstorder_RobustMeanAbsoluteDeviation

1423 wavelet-LLL_firstorder_RootMeanSquared

1424 wavelet-LLL_firstorder_Skewness

1425 wavelet-LLL_firstorder_TotalEnergy

1426 wavelet-LLL_firstorder_Uniformity

1427 wavelet-LLL_firstorder_Variance

1428 wavelet-LLL_glcm_Autocorrelation

1429 wavelet-LLL_glcm_ClusterProminence

1430 wavelet-LLL_glcm_ClusterShade

1431 wavelet-LLL_glcm_ClusterTendency

1432 wavelet-LLL_glcm_Contrast

1433 wavelet-LLL_glcm_Correlation

1434 wavelet-LLL_glcm_DifferenceAverage

1435 wavelet-LLL_glcm_DifferenceEntropy

1436 wavelet-LLL_glcm_DifferenceVariance

1437 wavelet-LLL_glcm_Id

1438 wavelet-LLL_glcm_Idm

1439 wavelet-LLL_glcm_Idmn

1440 wavelet-LLL_glcm_Idn

1441 wavelet-LLL_glcm_Imc1

1442 wavelet-LLL_glcm_Imc2

1443 wavelet-LLL_glcm_InverseVariance

1444 wavelet-LLL_glcm_JointAverage

1445 wavelet-LLL_glcm_JointEnergy

1446 wavelet-LLL_glcm_JointEntropy

1447 wavelet-LLL_glcm_MCC

1448 wavelet-LLL_glcm_MaximumProbability

1449 wavelet-LLL_glcm_SumAverage

1450 wavelet-LLL_glcm_SumEntropy

1451 wavelet-LLL_glcm_SumSquares

1452 wavelet-LLL_gldm_DependenceEntropy

1453 wavelet-LLL_gldm_DependenceNonUniformity

1454 wavelet-LLL_gldm_DependenceNonUniformityNormalized

1455 wavelet-LLL_gldm_DependenceVariance

1456 wavelet-LLL_gldm_GrayLevelNonUniformity

1457 wavelet-LLL_gldm_GrayLevelVariance

1458 wavelet-LLL_gldm_HighGrayLevelEmphasis

1459 wavelet-LLL_gldm_LargeDependenceEmphasis

1460 wavelet-LLL_gldm_LargeDependenceHighGrayLevelEmphasis

1461 wavelet-LLL_gldm_LargeDependenceLowGrayLevelEmphasis

1462 wavelet-LLL_gldm_LowGrayLevelEmphasis

1463 wavelet-LLL_gldm_SmallDependenceEmphasis

1464 wavelet-LLL_gldm_SmallDependenceHighGrayLevelEmphasis

1465 wavelet-LLL_gldm_SmallDependenceLowGrayLevelEmphasis

1466 wavelet-LLL_glszm_GrayLevelNonUniformity

1467 wavelet-LLL_glszm_GrayLevelVariance

1468 wavelet-LLL_glszm_HighGrayLevelZoneEmphasis

1469 wavelet-LLL_glszm_LargeAreaHighGrayLevelEmphasis

1470 wavelet-LLL_glszm_SizeZoneNonUniformity

1471 wavelet-LLL_glszm_SizeZoneNonUniformityNormalized

1472 wavelet-LLL_glszm_SmallAreaHighGrayLevelEmphasis

1473 wavelet-LLL_glszm_ZoneEntropy

1474 wavelet-LLL_glszm_ZoneVariance

1475 wavelet-LLL_glrlm_GrayLevelNonUniformity

1476 wavelet-LLL_glrlm_GrayLevelNonUniformityNormalized

1477 wavelet-LLL_glrlm_GrayLevelVariance

1478 wavelet-LLL_glrlm_HighGrayLevelRunEmphasis

1479 wavelet-LLL_glrlm_LongRunEmphasis

1480 wavelet-LLL_glrlm_LongRunHighGrayLevelEmphasis

1481 wavelet-LLL_glrlm_LongRunLowGrayLevelEmphasis

1482 wavelet-LLL_glrlm_LowGrayLevelRunEmphasis

1483 wavelet-LLL_glrlm_RunEntropy

1484 wavelet-LLL_glrlm_RunLengthNonUniformity

1485 wavelet-LLL_glrlm_RunLengthNonUniformityNormalized

1486 wavelet-LLL_glrlm_RunPercentage

1487 wavelet-LLL_glrlm_RunVariance

1488 wavelet-LLL_glrlm_ShortRunEmphasis

1489 wavelet-LLL_glrlm_ShortRunHighGrayLevelEmphasis

1490 wavelet-LLL_glrlm_ShortRunLowGrayLevelEmphasis

1491 wavelet-LLL_ngtdm_Busyness

1492 wavelet-LLL_ngtdm_Coarseness

1493 wavelet-LLL_ngtdm_Complexity

1494 wavelet-LLL_ngtdm_Contrast

1495 wavelet-LLL_ngtdm_Strength
